# Development and Biological Assessment of Thiazole-Based Pyridines for Targeted Therapy in Lung Cancer

**DOI:** 10.1021/acsomega.4c11252

**Published:** 2025-04-23

**Authors:** Demokrat Nuha, Sam Dawbaa, Asaf Evrim Evren, Zennure Şevval Çi̇yanci, Halide Edip Temel, Gülşen Akalin Çiftçi, Leyla Yurttaş

**Affiliations:** † Anadolu University, Faculty of Pharmacy, Department of Pharmaceutical Chemistry, Eskişehir 26470, Turkey; ‡ University for Business and Technology, Faculty of Pharmacy, Lagjja Kalabria, Prishtina 10000, Kosovo; § Al-Hikma University, Faculty of Medical Sciences, Department of Pharmacy, Dhamar Yemen; ∥ Thamar University, Faculty of Medical Sciences, Department of Doctor of Pharmacy (PharmD), Dhamar 87246, Yemen; ⊥ Bilecik Seyh Edebali University, Vocational School of Health Services, Pharmacy Services, Bilecik 11230, Turkey; # Anadolu University, Faculty of Pharmacy, Department of Biochemistry, Eskişehir 26470, Turkey

## Abstract

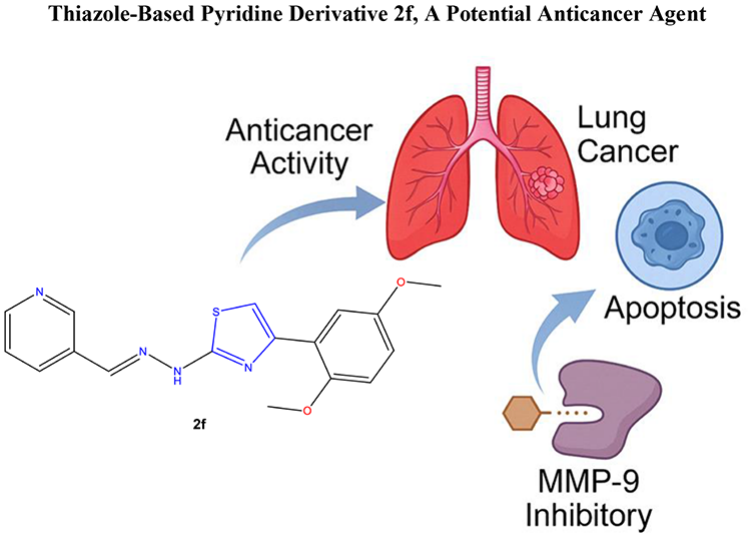

The study aims to synthesize, characterize, and evaluate a series of novel compounds for their potential anticancer activity targeting the A549 lung cancer cell line. The hydrazonothiazole-based pyridine compounds (**2a**–**2o**) were characterized through melting point analysis, ^1^H NMR, ^13^C NMR, and high-resolution mass spectrometry (HRMS). Their physicochemical properties were evaluated using *in silico* tools, and all compounds were found to comply with Lipinski’s drug-likeness rule, suggesting favorable drug-like characteristics. Biological activity studies revealed that all synthesized compounds exhibited potent cytotoxicity against the A549 cell line, with several compounds showing greater efficacy than the standard drug, cisplatin. Selectivity indices were also calculated, revealing that compounds **2b**, **2c**, **2f**, and **2m** exhibited enhanced selectivity for cancer cells relative to healthy cells. Mechanistic studies using flow cytometry demonstrated that these compounds induced apoptosis, with compound **2m** demonstrating the highest apoptotic activity. Mitochondrial membrane potential assay and caspase-3 activation confirmed the involvement of mitochondrial pathways in apoptosis induction. Furthermore, MMP-9 enzyme inhibition assays identified compound **2f** as the most effective inhibitor, with molecular docking and dynamics simulation studies confirming its strong binding interactions with key residues in the enzyme’s active site. Overall, this study suggests that the synthesized compounds, particularly **2b**, **2c**, **2f**, and **2m**, hold promise as potential anticancer agents for further development and optimization in the treatment of lung cancer.

## Introduction

Cancer represents a persistent and significant global health challenge, driving the continuous pursuit of innovative therapeutic strategies. In recent years, there has been a significant shift toward the exploration of small-molecule inhibitors as a promising strategy for effective anticancer therapies.^1,2^ These inhibitors are designed to selectively target specific molecular pathways closely linked to cancer progression, offering a focused approach to drug discovery in the oncology domain.^3−5^

Thiazole derivatives are a promising class of compounds that have garnered significant attention in this context.^6−8^ Characterized by their broad pharmacological diversity, thiazole derivatives exhibit structural and functional versatility that holds substantial potential for the development of novel anticancer agents. Their ability to modulate critical cellular processes involved in tumorigenesis positions them as promising candidates for further investigation in anticancer drug development endeavors.^9,10^

Thiazole derivatives exert their pharmacological effects through multifactorial mechanisms of action, allowing them to disrupt key molecular pathways critical for cancer cell survival, proliferation, and metastasis. By targeting specific cellular components or signaling cascades involved in cancer progression, these compounds have the potential of exerting potent anticancer activity while minimizing off-target effects and adverse reactions.^11−13^

The structural adaptability of thiazole derivatives further enhances their attractiveness as candidates for anticancer drug development. This structural versatility allows for fine-tuning of compound properties to optimize their efficacy, pharmacokinetics, and safety profiles. Through strategic modifications and rational design approaches, researchers can tailor thiazole derivatives to selectively interact with molecular targets implicated in specific cancer types or subtypes, thereby enhancing their therapeutic potential and clinical utility.^14−16^

Moreover, the diverse pharmacological activities exhibited by thiazole derivatives contribute to their appeal as valuable candidates in the therapeutic landscape against cancer. These compounds have demonstrated a spectrum of biological effects, ranging from cytotoxicity and apoptosis induction to antiangiogenic and antimetastatic properties. Such multifaceted pharmacological profiles offer opportunities for the development of combination therapies or personalized treatment approaches tailored to individual patient needs and tumor characteristics.^17,18^ Additionally, thiazole and pyridine are present in many structures of anticancer agents, such as dasatinib, alpelisib, dabrafenib, tiazofurin, epothilone, and patellamide A, either separately or together.^19^

In conclusion, thiazole derivatives represent a promising class of compounds with significant potential for anticancer drug discovery. Their structural versatility, multifaceted pharmacological activities, and targeted mechanisms of action position them as attractive candidates for further investigation and development as novel anticancer agents. By harnessing the unique properties of thiazole derivatives, researchers aim to advance the frontier of cancer therapy and improve outcomes for patients worldwide.^20,21^

This study aimed to synthesize a series of novel 4-phenyl-2-(pyridyl methylene)hydrazinyl) thiazole derivatives and evaluate their potential anticancer activities. The rational design of these compounds was guided by the structural features known to confer anticancer properties, with a focus on incorporating pyridyl methylene hydrazine moieties to enhance target specificity and therapeutic efficacy.

## Results and Discussion

### Chemistry

All of the compounds were synthesized according to the described methods. The yield of the compounds was determined to be between 72% and 85%. Purity determination and structure elucidation were achieved through melting point analysis, proton nuclear magnetic resonance (^1^H NMR) spectrometry, ^13^C nuclear magnetic resonance (^13^C NMR) spectrometry, elemental analysis, and high-resolution mass spectrometry (HRMS).

^1^H NMR spectra were obtained in a 300 MHz NMR spectrometer. Compounds **2a**, **2b**, **2f**, **2g**, **2k**, and **3l** showed a resonance for protons in the aliphatic region. Compounds **2a, 2f,** and **2k** have 2,3-dimethoxyphenyl moiety; hence, protons of the methoxy groups showed singlet signals at around 3.74 and 3.86 ppm. On the other hand, compounds **2b**, **2g**, and **2l** have a 4-methylsulfonylphenyl group, the methyl group of which shows a chemical shifts for its protons at around 3.25 ppm. The hydroxyl groups of compounds **2d**, **2i**, and **2n** were expected to show broad singlet peaks at around 5.34 ppm, but they could not be recognized by the spectrometer. It is thought that a stronger 400 or 500 MHz NMR spectrometer might be effective in showing the signals of the hydroxyl groups. The chemical identity of compounds **3d**, **3i**, and **3n** was confirmed by HRMS. The methylene bridge between the hydrazine and pyridine in all the compounds showed a singlet for its proton within the range of 7.53 to 8.20 ppm. The proton of the phenyl group at position 6 of compounds **2a**, **2f,** and **2k** is shifted to 7.60 ppm, more downfield than the protons at positions 3 and 4, which have chemical shifts at 6.88 and 7.05 ppm, respectively. The protons of the 4-methylsulfonylphenyl group of compounds **2b**, **2g**, and **2l** have signals at around 7.96 ppm for those at positions 3 and 5, and at 8.11 ppm for those at positions 2 and 6. The aromatic protons of the 3,4-dihydroxyphenyl group in compounds **2d**, **2i**, and **2n** appeared at around 6.76, 7.14, and 7.26 ppm for protons at positions 5, 6, and 2, respectively, where a meta coupling was recognized between protons 2 and 6, as shown by the coupling constant *J* in the analysis monographs. A similar sequence of the chemical shifts was recognized for the protons of the 3,4-dichlorophenyl group in compounds **2e**, **2j**, and **2o**, where protons at positions 5, 6, and 2 showed signals at around 7.67, 7.83, and 8.07 ppm, respectively. The protons of the phenyl group bonded to the thiazole ring of compounds **2c**, **2h**, and **2m** are more downfield-shifted than the protons of its 4-phenyl group. This is explained by the efficacy of the thiazole on the directly bonded phenyl group. Finally, the arrangement of the signals from the protons of pyridine varied. Different patterns of shifts were recognized according to the substitution on the pyridine, whether it is at the 2-, 3-, or 4-position. Every proton was assigned to its corresponding signal in the analytical monograph.

In the ^13^C NMR spectra, all of the aliphatic and aromatic carbons showed signals in the predicted chemical shift regions. The methoxy group carbons of compounds **2a**, **2f**, and **2k** showed signals between 55 and 57 ppm. The carbons of methyl sulfonyl groups in compounds **2b**, **2g**, and **2l** have chemical shifts of approximately 44.03 ppm. The remaining carbons were recognized between 100 and 180 ppm, as most of the carbons belong to aromatic cycles or conjugated bonds.

HRMS and elemental analysis confirmed the identity of the synthesized compounds, and the results are reported in detail in the analytical monographs; the spectra are illustrated in the Supporting Information file.

### Physicochemical Parameters

To evaluate the physicochemical properties of the compounds (Table 1), the number of hydrogen bond acceptors (HBA) and donors (HBD), molecular weights (MW), topological polar surface area (TPSA), and partition coefficient (Log P) were calculated *in silico* by SwissADME software. Additionally, gastrointestinal absorption (GI abs), blood–brain barrier (BBB) permeability, and compliance with Lipinski’s drug-likeness rule of five were determined. The molecular weights of the compounds were found to range between 312 and 358 g/mol, TPSA values were between 78.41 and 120.93 Å, and log *p* values were between 2.26 and 4.35. The number of HBA in the compounds was 3 and 5, while HBD was 1 and 3. GI absorption of the compounds was found to be high, whereas BBB permeability was found to be poor. All compounds complied with Lipinski’s drug-likeness rule. According to this rule,^22^ the molecular weight (MW) of an oral drug should be ≤500 g/mol, the lipophilicity coefficient LogP ≤ 5, the number of hydrogen bond donor groups ≤5, and the number of hydrogen bond acceptor groups ≤10. All synthesized compounds fulfilled these criteria, indicating favorable drug-like properties.

**Table 1:** Some Physicochemical Properties of the Compounds Calculated via SwissADME^a^

Comp.	MW	TPSA	Log P	HBA	HBD	GI abs	BBB	Lip
**2a**	340	96.87	3.11	5	1	High	No	+
**2b**	358	120.93	2.79	5	1	High	No	+
**2c**	356	78.41	4.45	3	1	High	No	+
**2d**	312	118.87	2.35	5	3	High	No	+
**2e**	349	78.41	4.20	3	1	High	No	+
**2f**	340	96.87	2.98	5	1	High	No	+
**2g**	358	120.93	2.67	5	1	High	No	+
**2h**	356	78.41	4.36	3	1	High	No	+
**2i**	312	118.87	2.26	5	3	High	No	+
**2j**	349	78.41	4.08	3	1	High	No	+
**2k**	340	96.87	2.99	5	1	High	No	+
**2l**	358	120.93	2.65	5	1	High	No	+
**2m**	356	78.41	4.35	3	1	High	No	+
**2n**	312	118.87	2.26	5	3	High	No	+
**2o**	349	78.41	4.08	3	1	High	No	+

a MW: molecular weight, TPSA: topological polar surface area (Å), Log P: partition coefficient, HBA: hydrogen bond acceptor, HBD: hydrogen bond donor, GI abs: gastrointestinal absorption, BBB: blood–brain barrier, Lip: Lipinski rule of five (+: no violation).

### Biological Results

#### Cytotoxicity

The synthesized final compounds (**2a**–**2o**) were initially evaluated for their cytotoxic effects to assess their anticancer activity against A549 lung cancer cells. The healthy cell line L929 (mouse fibroblast cell line) was also used to determine the selective antiproliferative properties of the compounds. Cisplatin was used as a standard drug, and the results are presented in μg/mL in Table 2. All compounds exhibited significant cytotoxic activity, demonstrating higher potency and lower IC_50_ values compared to cisplatin (IC_50_: 12.65 μg/mL) against the A549 cell line. Compound **2a** exhibited a lower inhibition concentration against the L929 healthy cell line (IC_50_: 4.36 μg/mL) than against the A549 cancer cell line (IC_50_: 7.30 μg/mL) and was evaluated as a toxic compound. Since the IC_50_ doses of the other compounds were found to be lower for the cancer cells, they can be considered partially selective. Detailed analysis revealed that the selectivity indices of compounds **2b**, **2c**, **2f**, **2g**, **2i**, **2m**, and **2o** exceeded 2.59, indicating selective cytotoxicity toward lung cancer cells.

**Table 2:** IC_50_ Values of the Compounds **2a**–**2o** (μg/mL)^a^

Compounds	A549	L929	SI
**2a**	7.30 ± 0.33	4.36 ± 0.32	0.60
**2b**	9.93 ± 0.37	31.25 ± 0.56	3.15
**2c**	10.12 ± 0.38	125.0 ± 0.64	12.35
**2d**	4.70 ± 0.60	9.20 ± 0.36	1.96
**2e**	4.56 ± 0.31	4.87 ± 0.30	1.07
**2f**	4.04 ± 0.29	13.97 ± 0.57	3.46
**2g**	8.40 ± 0.34	56.40 ± 0.59	6.71
**2h**	9.93 ± 0.42	15.22±0.53	1.53
**2i**	7.20 ± 0.95	>250.0	>34.72
**2j**	6.58 ± 0.35	9.76 ± 0.45	1.48
**2k**	6.07 ± 0.31	8.59 ± 0.32	1.42
**2l**	4.70 ± 0.30	6.74 ± 0.41	1.43
**2m**	12.05±0.43	31.25 ± 0.55	2.59
**2n**	4.23 ± 0.54	4.48 ± 0.68	1.06
**2o**	5.04 ± 0.31	51.88 ± 0.58	10.29
**Cisplatin**	12.65 ± 0.38	-	

a -: Not tested; SI: selectivity index

When the chemical structures of the compounds are analyzed, they can be divided into three main groups: those containing 2-pyridyl (**2a**–**2e**), 3-pyridyl (**2f**–**2j**), and 4-pyridyl (**2k**–**2o**). These compounds differ due to the substituents at the fourth position of the thiazole ring, with the corresponding compound was formed in each group. While derivatives containing 2-pyridyl and 4-pyridyl generally exhibited similar cytotoxic activity, enhanced activity was observed in derivatives with 2,5-dimethoxyphenyl (2f) and 4,4’-biphenyl (2h) substituents at the fourth position of the thiazole ring and with 3-pyridyl groups. In contrast, derivatives with 2-pyridyl and 4-pyridyl substituents remained prominent among other substituents. When each main group was evaluated within itself, it was determined that the derivatives containing 4,4’-biphenyl (**2c**, **2h**, and **2m**) were the least cytotoxic compounds in the group. This may be related to the low hydrogen bonding capacity compared to the other compounds. These compounds have 3 hydrogen bond acceptors and 1 hydrogen bond donor group. Although the numbers of these groups were similar in the compounds containing 3,4-dichlorophenyl, they did not cause a significant difference in the biological activity. Accordingly, in addition to the HBA and HBD numbers, the highest log *p* values in biphenyl-containing derivatives caused a decrease in activity.

#### Apoptosis Detection by Flow Cytometry

**Figure 1:**
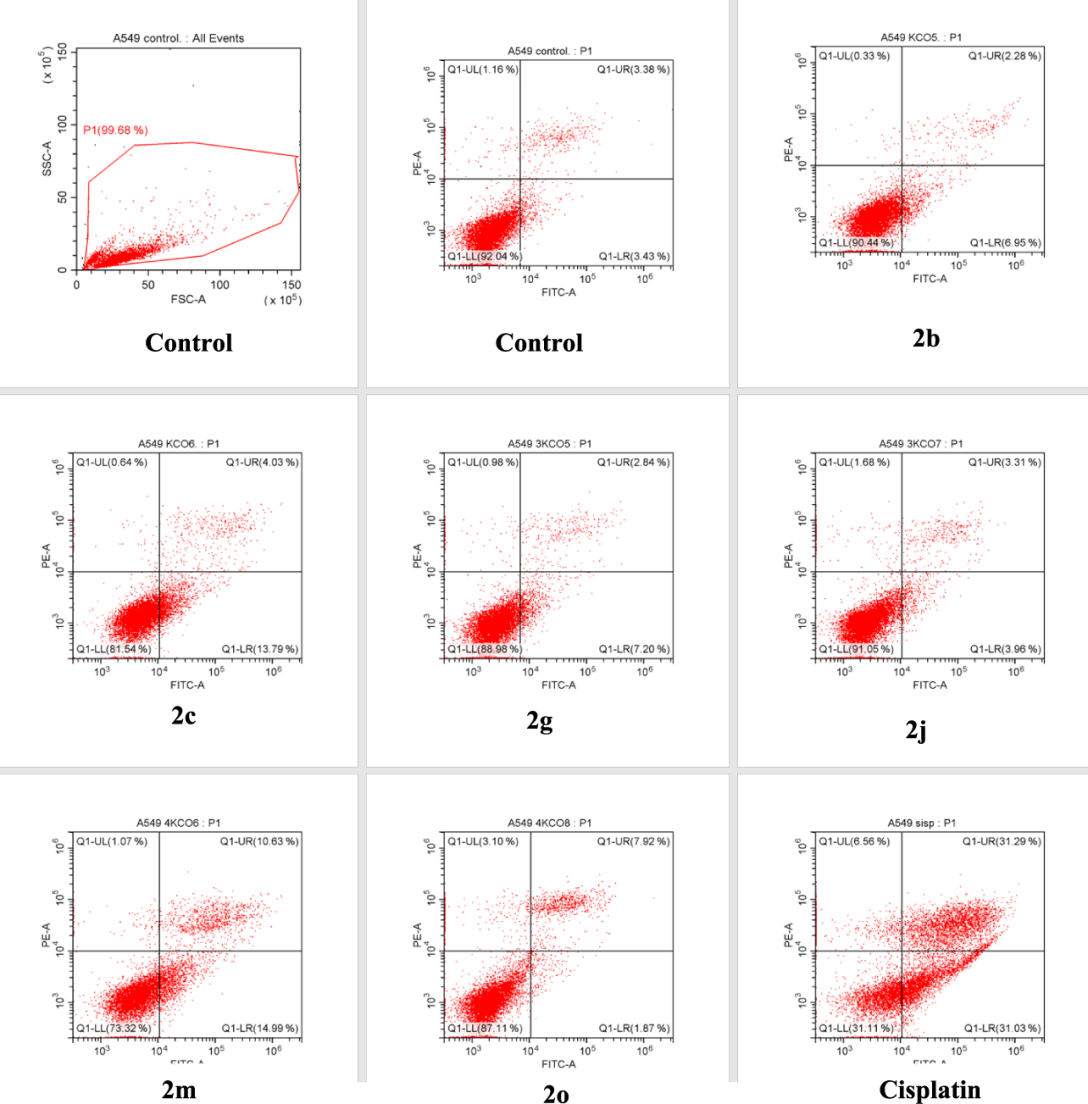
Quadrants of flow cytometric analysis.

To determine whether the antiproliferative effects of the compounds on the A549 cell line proceed through the apoptotic or necrotic pathway, the assay was performed with Annexin V-FITC/propidium iodide dyes in a flow cytometry device. A549 cells were incubated with IC_50_ values of the compounds and cisplatin for 24 h. At least 10,000 cells were analyzed, and quadrant analysis was performed. The results are presented as early apoptotic cells, late apoptotic cells, viable cells, and necrotic cells in Table 3, and as a diagram in Figure 1. In control cells, the number of viable cells was 92.04%, necrotic cells were 1.16%, early apoptotic cells were 3.43%, and late apoptotic cells were 3.28%. The standard drug cisplatin caused a total of 62.37% apoptosis in A549 cells, with 31.08% early apoptotic and 31.29% late apoptotic cell percentages. Among the compounds, **2m** was the most apoptosis-inducing compound, with 14.99% early apoptotic and 10.63% late apoptotic cell percentages. Compound **2m** induced necrosis at a rate of 1.07%. This compound was followed by compound **2c** with a 13.79% early apoptotic cells and 4.03% late apoptotic cells. Among the compounds, compound **2o** induced early apoptosis the least (1.87%) and necrosis the most (3.10%). As cells treated with compound 2j exhibited rates of apoptosis and necrosis similar to those of the control group, and compound 2b maintained a viable cell percentage of 90.44%, it was concluded that these compounds did not significantly induce apoptosis. Nonapoptotic cell death methods, such as necroptosis and pyroptosis, can be considered for the aforementioned compounds.

**Table 3:** Annexin V-FITC/Propidium Iodide Flow Cytometry Quadrant Analysis Percentages on A549 Cells Treated with Compounds and Cisplatin

	Q1-LR early apoptotic	Q1-UR late apoptotic	Q1-LL viable	Q1-UL necrotic
**Control**	3.43	3.28	92.04	1.16
**2b**	6.95	2.28	90.44	0.33
**2c**	13.79	4.03	81.54	0.64
**2g**	7.20	2.84	88.98	0.98
**2j**	3.96	3.31	91.05	1.68
**2m**	14.99	10.63	73.32	1.07
**2o**	1.87	7.92	87.11	3.10
**Cisplatin**	31.08	31.29	31.11	6.56

#### Evaluation of Mitochondrial Membrane Potential

**Figure 2:**
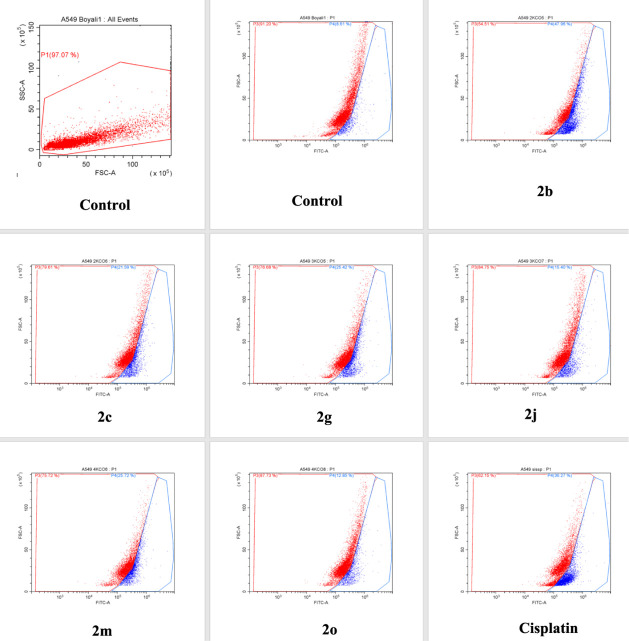
Mitochondrial membrane potential determining analysis.

The mitochondrial membrane potential provides information about the state of electrons during ATP production through oxidative phosphorylation, which is essential for cellular viability. Mitochondrial dysfunction occurs when this electrical regulation is disturbed and can occur in various diseases and the process of apoptosis.^23^ The results obtained for mitochondrial membrane potential are presented as percentages of polarization and depolarization for the compounds, cisplatin, and the control group in Table 4, and graphically in Figure 2. The tested compounds caused mitochondrial membrane depolarization ranging from 47.96% to 12.85%, while cisplatin caused depolarization of 36.27%. All of the compounds caused more depolarization than the control group. Among them, compound **2b** caused significantly higher mitochondrial membrane depolarization (47.96%), approximately 1.5 times higher than that exhibited by cisplatin. Compounds **2m**, **2g**, and **2c** followed this, with depolarization levels exceeding 21%. The mitochondrial dysfunction in compound **2m** can be considered a contributing factor to apoptosis.

**Table 4:** Percentages of Mitochondrial Membrane Polarized and Depolarized Cells on A549 Cells of Compounds

	% Mitochondrial membrane polarized cells	% Mitochondrial membrane depolarized cells
**Control**	91.20	8.61
**2b**	54.51	47.96
**2c**	79.61	21.59
**2g**	76.68	25.42
**2j**	84.75	15.40
**2m**	75.72	25.72
**2o**	87.73	12.85
**Cisplatin**	62.15	36.27

#### Caspase-3 Activation

Caspases can take part in the cell death mechanism through both intrinsic and extrinsic pathways, with caspase-3 being the first enzyme activated in the apoptosis process.^24^ Activation of the caspase-3 enzyme by the compounds, indicated as caspase-3 positive, and inhibition, indicated as caspase-3 negative, are presented in percentage terms in Table 5. It was found that **2b** (7.73%) and **2c** (5.95%) caused greater caspase-3 activation compared to all other compounds, although these effects were lower than that of cisplatin (11.65%).

**Table 5:** Percentages of Flow Cytometry Quadrant Analysis of Caspase-3 Activity on A549 Cells Treated with Compounds and Cisplatin

	% Caspase-3 positive cells	% Caspase-3 negative cells
**Control**	64.63	23.62
**2b**	7.73	84.70
**2c**	5.95	91.76
**2g**	0.38	73.16
**2j**	0.96	97.47
**2m**	0.86	69.82
**2o**	0.00	25.00
**Cisplatin**	11.65	75.56

#### Matrix Metalloproteinase-9 (MMP-9) Inhibition

Compounds (**2a**–**2o**) were tested to determine their MMP-9 enzyme inhibition potential. The compounds were tested at a concentration of 100 μM, and the standard molecule NNGH at a concentration of 1.3 μM. The results are presented in terms of percentage inhibition in Table 6. Among the compounds, **2f** exhibited 70.79% MMP-9 inhibition, while the other compounds were found to inhibit in the range of 40.59–9.40%. Since compound **2f** did not exhibit inhibition above 90% like the standard drug, no further study was carried out to determine IC_50_.

**Table 6:** MMP-9 Inhibition of the Compounds **2a**–**2o** at 100 Μm^a^

Compounds (100 μM)	MMP-9 inhibition %
**2a**	----
**2b**	----
**2c**	23.09 ± 2.08
**2d**	----
**2e**	30.29 ± 2.64
**2f**	70.79 ± 2.20
**2g**	8.63 ± 1.32
**2h**	----
**2i**	40.59 ± 3.01
**2j**	----
**2k**	----
**2l**	---
**2m**	9.40 ± 1.22
**2n**	18.99 ± 3.63
**2o**	----
NNGH (1.3 μM)	93.56 ± 1.78

a Not determined.

### *In Silico* Calculations

#### Molecular Docking Studies

**Figure 3:**
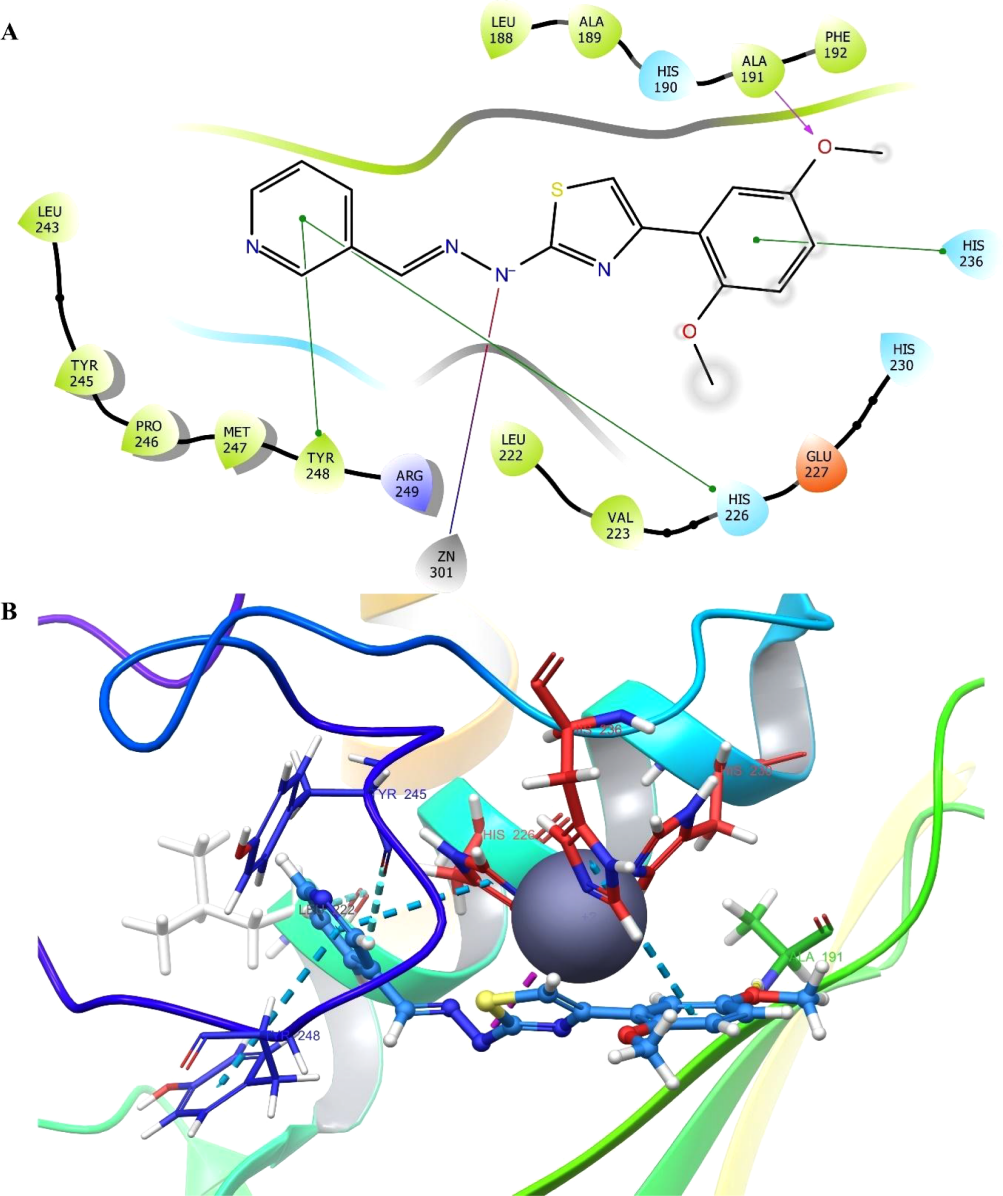
Molecular docking study of compound **2f** in drug-binding side of MMP-9. (A) 2D interaction diagram showing key binding interactions and (B) 3D binding pose of compound 2f in the MMP-9 active site.

According to the docking poses (Figure 3), compound **2f** interacted with Ala91 (H-bond), Leu222 (aromatic H-bond), His226 (π–π stacking), His236 (π–π stacking), Tyr245 (aromatic H-bond), Tyr248 (π–π stacking), and Zn301 (salt bridge). The residues, Zn and Histamines (sequence positions 226, 230, and 236), are identified as pivotal players in enzyme activity,^25−27^ thus, connections with these residues have a major impact on inhibitory activity. In addition to that, it seemed that **2f** also fitted well into the hydrophobic pocket, as it interacted with Leu222, Tyr245, and Tyr248 residues. The variable group, pyridine, showed affinity to the enzyme in two ways: making hydrophobic contacts and forming an H-bond in the hydrophobic cavity. As a result, all these interactions provided insight into the binding mode of the ligand-enzyme complex. Moreover, regarding its inhibition strength on the MMP-9 enzyme, there is a correlation between *in silico* and *in vitro* enzyme studies.

#### Molecular Dynamics Simulation (MDS) Studies

**Figure 4:**
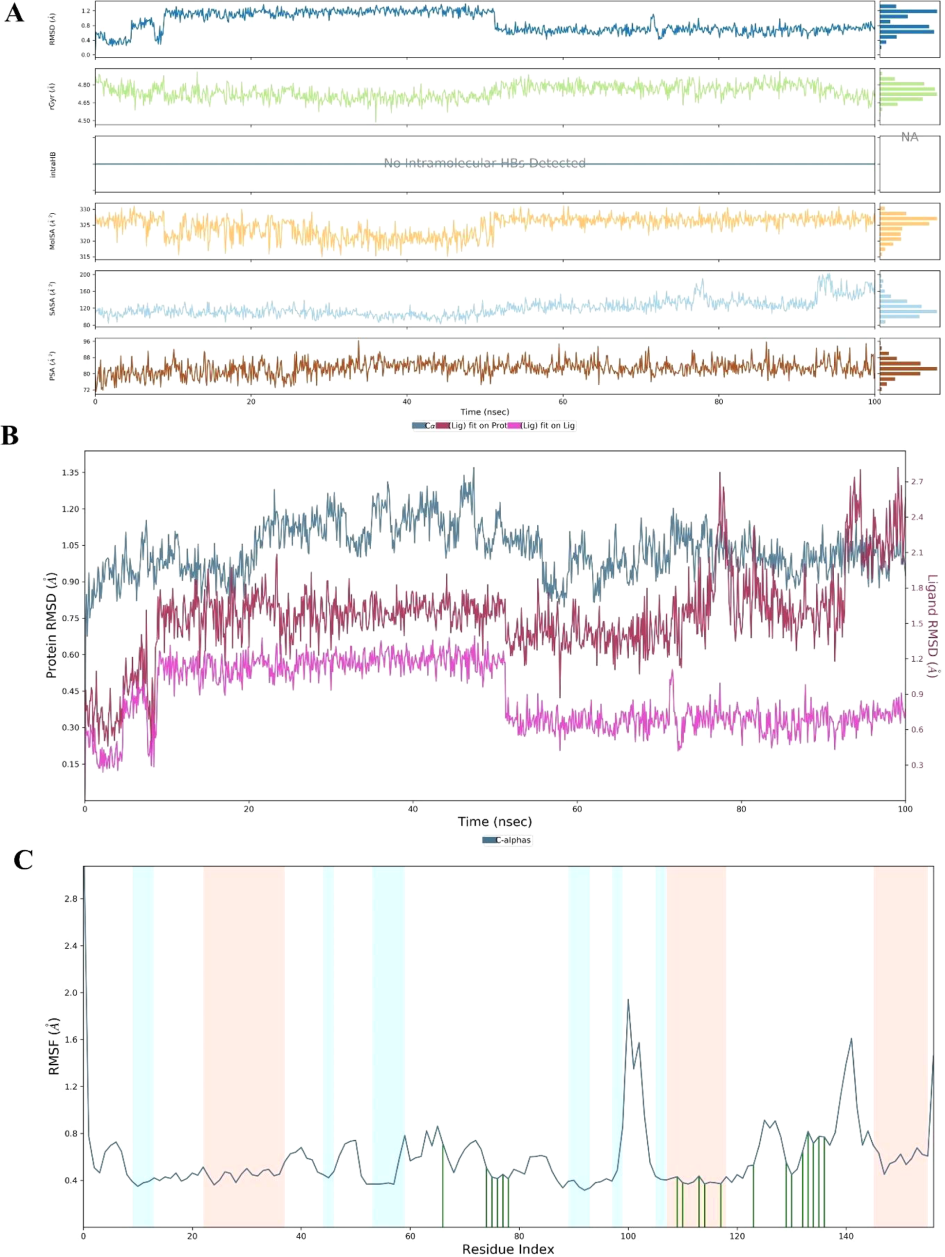
Stability plots of MDS. (A) Ligand properties; (B) RMSD values of protein (blue line), ligand fit protein (red line), and ligand fit ligand (purple line); (C) RMSF plot (red area represents helix amino acids, blue area represents ß-strand amino acids, and white area represents loop amino acids).

**Figure 5:**
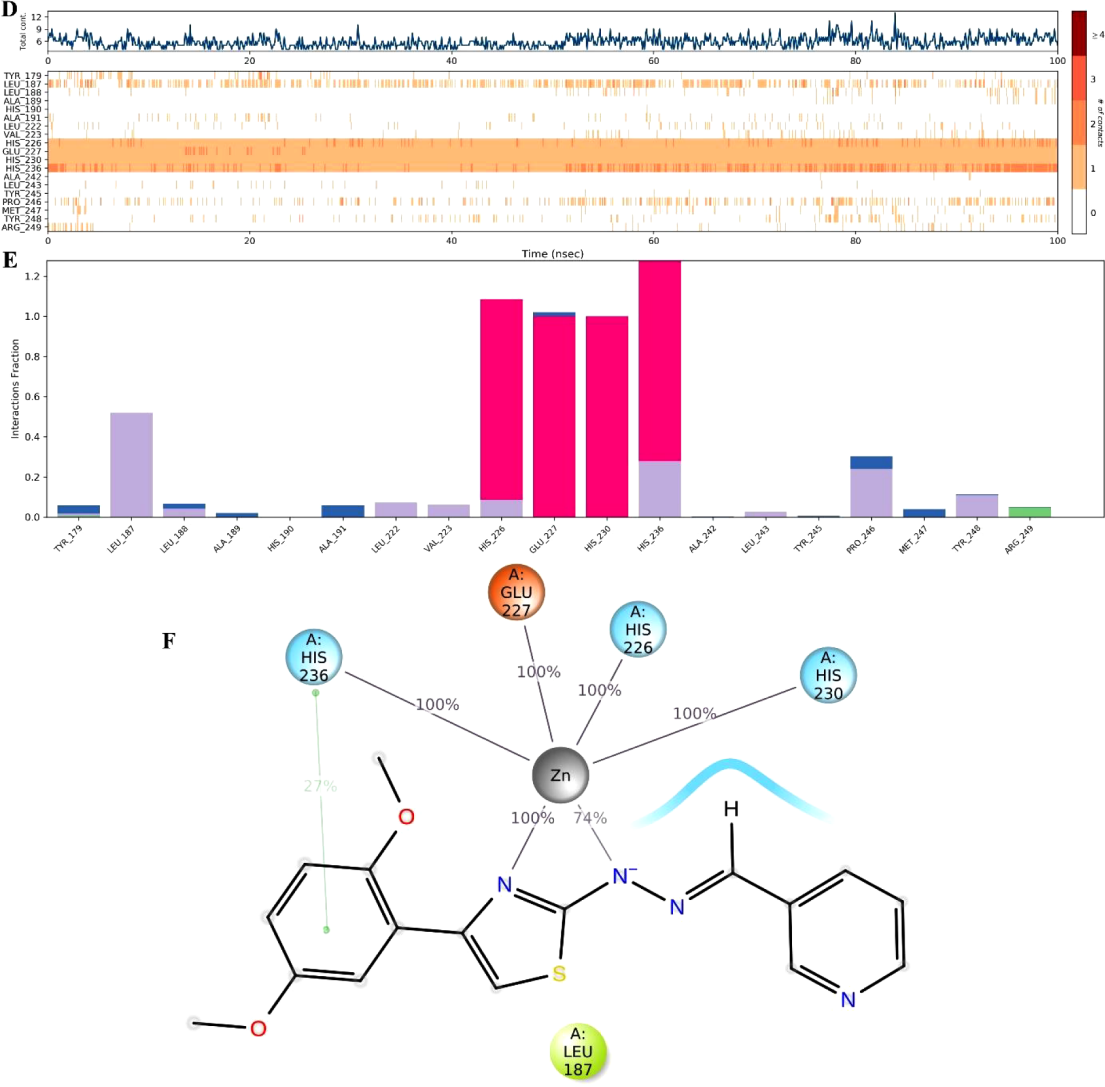
MDS interaction plots of the **2f**-MMP-9 system. (A) Total interaction number-residue-time plot, (B) interaction type-interaction fractions-residue index, (C) 2D plot of contact strength (cut off = 0.2).

The best docking pose was chosen for the MDS study to analyze the stability of interactions and the binding mode between **2f** and the MMP-9 enzyme. The stability results of the MDS are shown in Figure 4. The fluctuation of RMSD values of **2f** did not show drastic changes (±0.4 Å); however, there are three loops observed at around 5, 8, and 53 ns. Meanwhile, the rGyr values of **2f** showed small fluctuations, indicating that the whole mass of **2f** was protected during the interactions. In addition to that, the RMSD values were obtained as expected according to the literature.^28−30^ The RMSF values of the loop amino acids (white area) were calculated to be under 1.00 Å, indicating that the ligand-protein system’s stability was maintained. All these properties proved that the system’s stability was protected during the simulation time; thus, the interactions were reliable. The profile of these interactions is shown in Figure 5.

According to Figure 5 and **video**, MDS results are obtained similarly to docking results. The findings indicated that the interactions with Leu187, His226, Glu227, His230, His236, Pro246, and Zn played key roles in protecting the stability of the complex. Except for Leu187 and Pro246, these binding residues are observed continuously from the beginning of the simulation. As reported in previous studies,^31^ three histidine residues (sequence positions 226, 230, and 236), essential for inhibitory activity, interacted with compound **2f**. Therefore, it was suggested that the inhibitory potential of 3-pyridinyl and thiazolohydrazone moieties against MMP-9 enzyme was related to their bonding ability. There is no meaningful difference between the 2-, 3-, and 4-pyridinyl moieties considering IC_50_ values on A549 cancer cells or MMP-9 inhibition; however, the variable group, the pyridine ring, showed affinity to the enzyme pocket due to its ability of making hydrophobic contacts and forming an H-bond. As a result, all these interactions clarified the binding mode of the ligand-enzyme complex during environmental changes and time.

In addition to the above findings, 3,4-dihydroxyphenyl (**2d**, **2i**, **2n**) and 3,4-dichlorophenyl (**2e**, **2j**, **2o**) moieties were two or three times more cytotoxic than 4,4’-biphenyl ring system (**2c**, **2h**, **2m**) against A549 cells. Also, as **2f**’s analogs (**2a**, **2k**) were less active than **2f** (2,5-dimethoxypehnyl) or inactive against the MMP-9 enzyme, the substituent effects of thiazole ring have a major impact in biological activity.

Generally, pyridine-based thiazole derivatives show cytotoxic activity; nevertheless, the mode of action depends on the substituent of the thiazole. When these findings were compared with 4-pyridine thiazole (analog 1),^32^ 2/4-pyridinohydrazonethiazole (analog 2),^33^ and 2-pyridinaminothiazole (analog 3).^34^ Compounds **2a**–**2o** showed significant cytotoxic effects compared to analog 1 but not analog 2 or 3. This comparison suggests that at least one nitrogen atom should be involved in linking pyridine and thiazole rings, which increases the cytotoxicity against A549 cells and induces apoptotic effects.

## Conclusions

**Scheme 1:**
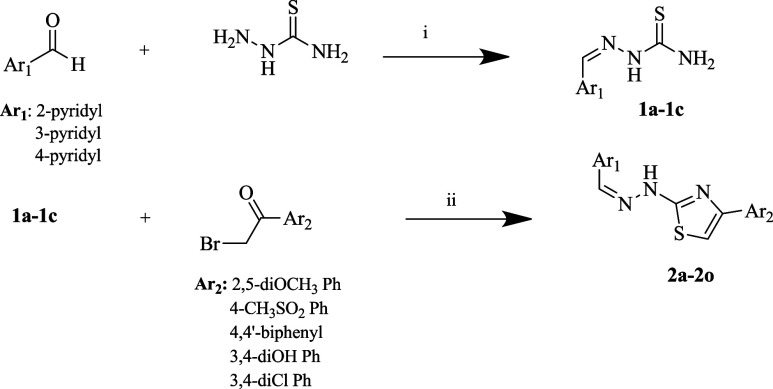
Synthesis Method of Compounds **2a**–**2o**, **i:** EtOH, Reflux 4 h, **ii:** EtOH, Reflux 3 h

In conclusion, this research successfully demonstrated the synthesis and characterization of novel compounds exhibiting pronounced cytotoxic properties and significant anticancer potential. The synthesis was conducted following the procedures outlined in Scheme 1. The structural integrity and purity of the synthesized compounds were confirmed through various spectroscopic techniques, including ^1^H NMR, ^13^C NMR, and HRMS. Physicochemical properties such as molecular weight, TPSA, and log P were determined using SwissADME software, and all compounds adhered to Lipinski’s rule of five, thereby indicating favorable drug-likeness and potential oral bioavailability.

The biological evaluation revealed that the synthesized compounds exhibited potent cytotoxic effects against the A549 lung cancer cell line, with several compounds demonstrating higher efficacy than the reference drug, cisplatin. Compounds **2b**, **2c**, **2f**, **2g**, **2i**, **2m**, and **2o** exhibited high selectivity indices, indicating selective cytotoxicity toward cancer cells compared to healthy cells. Further mechanistic flow cytometry studies revealed that compounds **2m** and **2c** induced apoptosis, which was characterized by significant increases in early and late apoptotic cell populations. Notably, compound 2m demonstrated the highest apoptosis induction, underscoring its potential as a lead compound for further therapeutic development.

Additionally, mitochondrial membrane potential assays indicated that most of the compounds caused mitochondrial depolarization, which is a hallmark of mitochondrial dysfunction and a precursor to apoptosis. Compound **2b** displayed the highest degree of mitochondrial depolarization, further supporting its strong pro-apoptotic activity. Caspase-3 activation assays demonstrated that compounds **2b** and **2c** activated the caspase-3 enzyme, confirming their role in triggering apoptosis through caspase-dependent pathways.

MMP-9 inhibition studies revealed that compound **2f** exhibited the highest inhibition potential among the series, suggesting its utility in targeting MMP-9-associated metastatic pathways. Molecular docking and molecular dynamics simulations provided insights into the binding interactions of compound **2f** with the MMP-9 enzyme, highlighting critical interactions with key residues and confirming the stability of the ligand-enzyme complex throughout the simulation period.

Overall, the study provides a comprehensive evaluation of the synthesized compounds, emphasizing their potential as promising anticancer agents targeting various biological pathways. Further *in vivo* studies and optimization of these compounds could pave the way for the development of new therapeutic candidates for the treatment of lung cancer.

## Materials and Methods

All chemicals were obtained from Sigma-Aldrich Chemical Co. (Sigma-Aldrich Corp., St. Louis, MO, USA) and Merck Chemicals (Merck KGaA, Darmstadt, Germany). Melting points (mp) were determined using an MP90 digital melting point apparatus (Mettler Toledo, Ohio, USA) and were uncorrected. Reactions were monitored by thin-layer chromatography (TLC) using Silica Gel 60 F_254_ TLC plates (Merck KGaA, Darmstadt, Germany). ^1^H NMR and ^13^C NMR spectra were recorded using a Bruker digital FT-NMR spectrometer (Bruker Bioscience, Billerica, MA, USA) operating at 300 and 75 MHz, respectively. Samples were prepared in DMSO-d_6_ for those NMR analyses. Mass analysis was achieved using a Shimadzu 8040 LC/MS/MS system (Shimadzu, Tokyo, Japan) and an Advion Expression Compact Mass Spectrometer (Advion Interchim Scientific, NY, USA).

### Chemistry

#### Synthesis of 2-(Pyridylmethylene)hydrazinecarbothioamide (**1a–1c**)

Yield: 70–85%. Pyridine-2-carbaldehyde, pyridine-3-carbaldehyde, and pyridine-4-carbaldehyde (3 g, 28.009 mmol each) were reacted separately with thiosemicarbazide (2.553 g, 28.009 mmol) in ethanol under reflux for 4 h to obtain 2-(pyridin-2-ylmethylene), 2-(pyridin-3-ylmethylene), and 2-(pyridin-4-ylmethylene) hydrazinecarbothioamide, respectively, as illustrated in Scheme 1. The formed products were precipitated and collected via filtration while the reaction mixtures were still hot. The products were obtained in pure form without the need for further recrystallization.

#### General Synthesis of 4-Phenyl-2-(2-(pyridylmethylene)hydrazinyl)thiazole Derivatives (**2a–2o**)

Compounds **1a, 1b**, and **1c** (0.3 g, 1.665 mmol each) were separately reacted with different 2-bromoacetophenone derivatives (1 equiv) in ethanol under reflux for 3 h. The precipitate formed from each reaction was obtained via filtration while the mixture was hot. The pure products required no further purification.

#### 4-(2,5-Dimethoxyphenyl)-2-[2-(pyridin-2 ylmethylene)hydrazinyl]thiazole (**2a**)

Yield: 81%, mp 214–215 °C, ^1^H NMR (300 MHz, DMSO-d_6_, ppm) δ 3.77 (s, 3H, OCH_3_), 3.86 (s, 3H, OCH_3_), 6.88 (dd, *J* = 8.95, 3.21 Hz, 1H, phenyl-4), 7.05 (d, *J* = 8.97 Hz, 1H, phenyl-3), 7.53 (s, 2H, thiazole-5 and **H–C**=N), 7.56 (ddd, *J* = 7.61, 5.02, 1.06 Hz, 1H, pyridine-5), 7.60 (d, *J* = 3.21 Hz, 1H, phenyl-6), 7.77 (d, *J* = 7.92 Hz, 1H, pyridine-3), 8.09 (td, *J* = 7.76, 1.76 Hz, 1H, pyridine-4), 8.85 (d, *J* = 4.11 Hz, 1H, pyridine-6), and 14.75 (brs, 1H, C=N–**NH**−). ^13^C NMR (300 MHz, DMSO-d_6_, ppm) δ 56.04, 56.44, 109.79, 113.18, 113.99, 114.89, 124.68, 126.31, 133.86, 138.90, and 148.53. Compact mass spectrometry (CMS) (*m*/*z*): [M + 1]^+^ calculated: 341.1; found: 341.3. HRMS (*m*/*z*): [M + 1]^+^ calculated: 341.1067; found: 341.1083.

#### 4-(4-(Methylsulfonyl)phenyl)-2-(2-(pyridin-2-ylmethylene)hydrazinyl)thiazole (**2b**)

Yield: 70%, mp 245–246 °C, ^1^H NMR (300 MHz, DMSO-d_6_, ppm) δ 3.25 (s, 3H, SO_2_–**CH**_**3**_), 7.77 (t, *J* = 6.56 Hz, 1H, pyridine-5), 7.80 (s, 1H, thiazole-5), 7.96 (d, *J* = 8.61 Hz, 2H, phenyl-3,5), 8.11 (d, *J* = 8.53 Hz, 2H, phenyl-2,4), 8.16 (d, *J* = 8.92 Hz, 1H, pyridine-3), 8.20 (s, H, −**HC**=N−), 8.35 (td, *J* = 7.88, 1.16 Hz, 1H, pyridine-4), 8.76 (d, *J* = 4.77 Hz, 1H, pyridine-6), and 13.18 (brs, 1H, C=N–**NH**−). ^13^C NMR (300 MHz, DMSO-d_6_, ppm) δ 44.04 (SO_2_–**CH**_**3**_), 109.48, 122.55, 125.56, 126.62, 127.69, 128.07, 129.00, 135.13, 139.19, 139.90, 143.69, 144.57, 149.11, 149.51, and 167.88. Compact mass spectrometry (CMS) (*m*/*z*): [M + 1]^+^ calculated: 359.6; found: 359.3. HRMS (*m*/*z*): [M + 1]^+^ calculated: 359.0631; found: 359.0645.

#### 4-([1,1’-Biphenyl]-4-yl)-2-(2-(pyridin-2-ylmethylene)hydrazinyl)thiazole (**2c**)

Yield: 75%, mp 180–181 °C, ^1^H NMR (300 MHz, DMSO-d_6_, ppm) δ 7.37 (t, *J* = 7.28 Hz, 1H, phenyl-4), 7.47 (t, *J* = 7.50 Hz, 2H, phenyl-3, 5), 7.53 (s, 1H, thiazole-5 −**HC**=N−), 7.64 (t, *J* = 6.72 Hz, pyridine-5), 7.71 (d, *J* = 7.14 Hz, 2H, phenyl-2, 6), 7.73 (d, *J* = 8.44 Hz, 2H, thiazole-phenyl-3,5), 7.96 (d, *J* = 8.44 Hz, 2H, thiazole-phenyl-2, 6), 8.08 (d, *J* = 8.01 Hz, 1H, pyridine-3), 8.17 (s, 1H, **H–C**=N), 8.20 (t, *J* = 8.01 Hz, 1H, pyridine-4), and 8.70 (d, *J* = 5.94 Hz, 1H, pyridine-6). ^13^C NMR (300 MHz, DMSO-d_6_, ppm) δ 105.76, 121.66, 124.68, 125.05, 126.59, 126.96, 127.38, 128.00, 129.45, 133.87, 136.90, 139.72, 140.03, 141.77, 146.12, 148.58, 150.55, 167.83, and 168.48. Compact mass spectrometry (CMS) (*m*/*z*): [M + 1]^+^ calculated: 356.11; found: 357.4. HRMS (*m*/*z*): [M + 1]^+^ calculated: 357.1168; found: 357.1178.

#### 4-(2-(2-(Pyridin-2-ylmethylene)hydrazinyl)thiazol-4-yl)benzene-1,2-diol (**2d**)

Yield: 84%, mp 197–198 °C, ^1^H NMR (300 MHz, DMSO-d_6_, ppm) δ 6.76 (d, *J* = 8.22 Hz, 1H, phenyl-5), 7.11 (s, 1H, thiazole-5), 7.14 (dd, *J* = 8.19, 2.10 Hz, 1H, phenyl-6), 7.26 (d, *J* = 2.10 Hz, 1H, phenyl-2), 7.66 (t, *J* = 6.38 Hz, 1H, pyridine-5), 8.10 (d, *J* = 7.96 Hz, 1H, pyridine-3), 8.21 (s, 1H, H–C=N), 8.26 (t, *J* = 8.11, 1H, pyridine-4), and 8.69 (d, *J* = 5.27, 1H, pyridine-6). ^13^C NMR (300 MHz, DMSO-d_6_, ppm) δ 102.64, 113.82, 116.17, 117.12, 117.50, 121.93, 125.17, 126.43, 135.22, 142.82, 144.89, 145.68, 145.92, 149.91, and 167.36. Compact mass spectrometry (CMS) (*m*/*z*): [M + 1]^+^ calculated: 312.7; found: 313.3. HRMS (*m*/*z*): [M + 1]^+^ calculated: 313.0754; found: 313.0760.

#### 4-(3,4-Dichlorophenyl)-2-(2-(pyridin-2-ylmethylene)hydrazinyl)thiazole (2e)

Yield: 73%, mp 251–252 °C, ^1^H NMR (300 MHz, DMSO-d_6_, ppm) δ 7.67 (d, *J* = 8.67 Hz, 1H, phenyl-5), 7.70 (s, 1H, thiazol-5), 7.73 (t, *J* = 6.60 Hz, 1H, pyridine-5), 7.83 (dd, *J* = 8.45, 2.06 Hz, 1H, phenyl-6), 8.01 (d, *J* = 2.04 Hz, 1H, phenyl-2), 8.13 (d, *J* = 7 = 8.25, 1H, pyridine-3), 8.18 (s, 1H, **H–C**=N), 8.31 (td, *J* = 7.89, 1.32 Hz, 1H, pyridine-4), 8.75 (d, *J* = 5.49 Hz, 1H, pyridine-6), and 13.05 (brs, 1H, C=N–**NH**−). ^13^C NMR (300 MHz, DMSO-d_6_, ppm) δ 108.06, 122.32, 125.44, 126.07, 127.62, 130.42, 131.42, 131.96, 135.26, 135.59, 143.21, 144.95, 148.59, 149.44, and 167.80. Compact mass spectrometry (CMS) (*m*/*z*): [M + 1]^+^ calculated: 348.0; found: 349.2. HRMS (*m*/*z*): [M + 1]^+^ calculated: 349.0076; found: 349.0078.

#### 4-(2,5-Dimethoxyphenyl)-2-(2-(pyridin-3-ylmethylene)hydrazinyl)thiazole (**2f**)

Yield: 79%, mp 216–217 °C, ^1^H NMR (300 MHz, DMSO-d_6_, ppm) δ 3.74 (s, 3H, OCH_3_), 3.86 (s, 3H, OCH_3_), 6.87 (dt, *J* = 8.93, 2.98 Hz, 1H, phenyl-3), 7.05 (dd, *J* = 9.00, 2.69 Hz, 1H, phenyl-4), 7.51 (s, 1H, thiazol-5), 7.61 (d, *J* = 2.95, 1H, phenyl-6), 7.93 (tt, *J* = 5.52, 2.53 Hz, 1H, pyridine-5), 8.15 (s, 1H, H–C=N−), 8.60 (d, *J* = 7.19 Hz, 1H, pyridine-4), 8.78 (dd, *J* = 5.39, 2.79 Hz, 1H, pyridine-6), 9.05 (s, 1H, pyridine-2). ^13^C NMR (300 MHz, DMSO-d_6_, ppm) δ 55.81, 56.37, 109.44, 113.28, 114.20, 123.64, 126.86, 133.62, 135.87, 139.27, 142.56, 143.59, 151.39, 153.95, and 166.48. Compact mass spectrometry (CMS) (*m*/*z*): [M + 1]^+^ calculated: 341.1; found: 341.3. HRMS (*m*/*z*): [M + 1]^+^ calculated: 341.1067; found: 341.1073.

#### 4-(4-(Methylsulfonyl)phenyl)-2-(2-(pyridin-3-ylmethylene)hydrazinyl)thiazole (**2g**)

Yield: 78%, mp 280–281 °C, ^1^H NMR (300 MHz, DMSO-d_6_, ppm) δ 3.24 (s, 3H, SO_2_–**CH**_**3**_), 7.74 (s, 1H, thiazole-5), 7.91–7.93 (m, 1H, pyridine-5), 7.96 (d, *J* = 8.55 Hz, 2H, phenyl-3,5), 8.12 (d, *J* = 8.58 Hz, 2H, phenyl-2,4), 8.17 (s, H, −**HC**=N−), 8.60 (dt, *J* = 8.18, 1.55 Hz, 1H, pyridine-4), 8.78 (dd, *J* = 5.43, 1.32 Hz, 1H, pyridine-6), 9.06 (d, *J* = 1.74 Hz, 1H, pyridine-2), and 12.82 (brs, 1H, C=N–**NH**−). ^13^C NMR (300 MHz, DMSO-d_6_, ppm) δ 44.03 (SO_2_–**CH**_**3**_), 108.61, 126.58, 126.85, 128.05, 133.39, 136.52, 139.32, 139.82, 142.77, 144.22, 149.46, and 168.46. Compact mass spectrometry (CMS): [M + 1]^+^ calculated: 359.6; found: 359.3. HRMS (*m*/*z*): [M + 1]^+^ calculated: 359.0631; found: 359.0641.

#### 4-([1,1’-Biphenyl]-4-yl)-2-(2-(pyridin-3-ylmethylene)hydrazinyl)thiazole (**2h**)

Yield: 74%, mp 243–244 °C, ^1^H NMR (300 MHz, DMSO-d_6_, ppm) δ 7.37–7.40 (m, 1H, Ar–H), 7.45–7.50 (m, 3H, Ar–H), 7.71–7.76 (m, 4H, Ar–H), 7.90–7.92 (m, 1H, pyridine-5), 7.96 (dd, *J*1 = 8.33 Hz, *J*2 = 2.66 Hz, 2H, Ar–H), 8.16 (d, *J* = 2.58 Hz, 1H, Ar–H), 8.59 (d, *J* = 6.94 Hz, 1H, pyridine-4), 8.79 (d, *J* = 5.39 Hz, 1H, pyridine-6), and 9.06 (s, 1H, pyridine-2). ^13^C NMR (300 MHz, DMSO-d_6_, ppm) δ 105.42, 126.59, 126.95, 127.35, 127.99, 129.46, 130.39, 133.93, 134.03, 135.66, 139.66, 140.03, 140.39, 141.54, 142.86, 150.65, and 168.09. Compact mass spectrometry (CMS) (*m*/*z*): [M + 1]^+^ calculated: 357.11; found: 357.4. HRMS (*m*/*z*): [M + 1]^+^ calculated: 357.1168; found: 357.1177.

#### 4-(2-(2-(Pyridin-3-ylmethylene)hydrazinyl)thiazol-4-yl)benzene-1,2-diol (**2i**)

Yield: 85%, mp 248–249 °C, ^1^H NMR (300 MHz, DMSO-d_6_, ppm) δ 6.76 (d, *J* = 8.22 Hz, 1H, phenyl-5), 7.05 (s, 1H, thiazole-5), 7.13 (dd, *J* = 8.18, 1.97 Hz, 1H, phenyl-6), 7.26 (d, *J* = 2.07 Hz, 1H, phenyl-2), 7.98 (dd, *J* = 8.14, 5.57 Hz, 1H, pyridine-5), 8.19 (s, 1H, H–C=N), 8.66 (d, *J* = 8.22, 1H, pyridine-4), 8.80 (dd, *J* = 5.52, 1.05 Hz, 1H, pyridine-6), and 9.06 (d, *J* = 1.65 Hz, 1H, pyridine-2). ^13^C NMR (300 MHz, DMSO-d_6_, ppm) δ 101.99, 113.86, 116.19, 117.48, 126.52, 127.31, 134.18, 135.49, 140.19, 141.10, 142.40, 145.66, 145.87, 151.26, and 167.69. Compact mass spectrometry (CMS) (*m*/*z*): [M + 1]^+^ calculated: 313.1; found: 313.3.

#### 4-(3,4-Dichlorophenyl)-2-(2-(pyridin-3-ylmethylene)hydrazinyl)thiazole (**2j**)

Yield: 83%, mp 271–272 °C, ^1^H NMR (300 MHz, DMSO-d_6_, ppm) δ 7.65 (s, 1H, thiazole-5), 7.68 (d, *J* = 8.49 Hz, 1H, phenyl-5), 7.84 (dd, *J* = 8.44, 2.04 Hz, 1H, phenyl-6), 7.97 (dd, *J* = 8.16, 5.49 Hz, 1H, pydidine-5), 8.08 (d, *J* = 2.041 Hz, 1H, phenyl-2), 8.17 (s, 1H, H–C=N), 8.64 (dt, *J* = 8.18, 1.54, 1H, pyridine-4), 8.82 (dd, *J* = 5.49, 1.20 Hz, 1H, pyridine-6), 9.08 (d, *J* = 1.68 Hz, 1H, pyridine-2), and 12.78 (brs, 1H, C=N–**NH**−). ^13^C NMR (300 MHz, DMSO-d_6_, ppm) δ 107.35, 126.05, 127.05, 127.62, 130.32, 131.41, 131.93, 133.63, 135.42, 136.27, 139.83, 142.24, 143.64, 148.53, and 168.31. Compact mass spectrometry (CMS) (*m*/*z*): [M + 1]^+^ calculated: 348.0; found: 349.2. HRMS (*m*/*z*): [M + 1]^+^ calculated: 349.0076; found: 349.0069.

#### 4-(2,5-Dimethoxyphenyl)-2-(2-(pyridin-4-ylmethylene)hydrazinyl)thiazole (**2k**)

Yield: 72%, mp 233–234 °C, ^1^H NMR (300 MHz, DMSO-d_6_, ppm) δ 3.74 (s, 3H, OCH_3_), 3.86 (s, 3H, OCH_3_), 6.88 (dd, *J* = 8.94, 3.21 Hz, 1H, phenyl-3), 7.05 (d, *J* = 9.03 Hz, 1H, phenyl-4), 7.59–7.60 (m, 2H, thiazole-5 and phenyl-6), 8.11 (d, *J* = 6.79 Hz, 2H, pyridine-3, 5), 8.14 (s, 1H, **H–C**=N), 8.83 (dd, *J* = 6.81 Hz, 2H, pyridine-2, 6), and 13.20 (brs, 1H, −C=**N–H**). ^13^C NMR (300 MHz, DMSO-d_6_, ppm) δ 55.86, 56.32, 110.65, 113.30, 114.22, 114.38, 122.73, 123.38, 123.99, 135.51, 142.83, 143.07, 150.51, 151.39, 153.41, and 179.44. Compact mass spectrometry (CMS) (*m*/*z*): [M + 1]^+^ calculated: 341.1; found: 341.3. HRMS (*m*/*z*): [M + 1]^+^ calculated: 341.1067; found: 341.1074.

#### 4-(4-(Methylsulfonyl)phenyl)-2-(2-(pyridin-4-ylmethylene)hydrazinyl)thiazole (**2l**)

Yield: 77%, mp 298–299 °C, ^1^H NMR (300 MHz, DMSO-d_6_, ppm) δ 3.26 (s, 3H, SO_2_–**CH**_**3**_), 7.84 (s, 1H, thiazole-5), 7.98 (d, *J* = 8.62 Hz, 2H, phenyl-3,5), 8.14 (d, *J* = 8.25 Hz, 4H, phenyl-2,6 and pyridine-3,5), 8.17 (s, H, −**HC**=N−), 8.85 (d, *J* = 6.72 Hz, 2H, pyridine-2,4), and 13.28 (brs, 1H, C=N–**NH**−). ^13^C NMR (300 MHz, DMSO-d_6_, ppm) δ 44.02 (SO_2_–**CH**_**3**_), 109.74, 122.77, 126.63, 128.09, 136.36, 139.16, 139.97, 143.42, 149.66, 149.78, and 167.97. Compact mass spectrometry (CMS): (*m*/*z*): [M + 1]^+^ calculated: 359.06; found: 359.3. HRMS (*m*/*z*): [M + 1]^+^ calculated: 359.0631; found: 359.0640.

#### 4-([1,1’-Biphenyl]-4-Yl)-2-(2-(pyridin-4-ylmethylene)hydrazinyl)thiazole (**2m**)

Yield: 75%, mp 252–253 °C, ^1^H NMR (300 MHz, DMSO-d_6_, ppm) δ 7.38 (t, *J* = 7.27 Hz, 1H, phenyl-4), 7.49 (t, *J* = 7.47 Hz, 2H, phenyl-3, 5), 7.61 (s, 1H, thiazole-5), 7.73 (d, *J* = 8.43 Hz, 2H, thiazole-**phenyl-3,5**), 7.76 (d, *J* = 8.19 Hz, 2H, thiazole-**phenyl-2,6**), 7.99 (d, *J* = 8.44 Hz, 2H, thiazole-phenyl-2,6), 8.15 (d, *J* = 6.78, 2H, pyridine-3, 5), 8.18 (s, 1H, −**HC**=N−), 8.85 (d, *J* = 6.75 Hz, 2H, pyridine-2, 6), and 13.32 (brs, 1H, C=N–**NH**). ^13^C NMR (300 MHz, DMSO-d_6_, ppm) δ 106.43, 122.76, 123.94, 126.61, 126.97, 127.41, 128.03, 129.47, 133.72, 135.91, 136.79, 139.82, 139.99, 143.03, 143.20, 150.26, 150.89, 167.68, and 179.43. Compact mass spectrometry (CMS) (*m*/*z*): [M + 1]^+^ calculated: 357.11; found: 357.4. HRMS (*m*/*z*): [M + 1]^+^ calculated: 357.1168; found: 357.1178.

#### 4-(2-(2-(Pyridin-4-ylmethylene)hydrazinyl)thiazol-4-yl)benzene-1,2-diol (**2n**)

Yield: 81%, mp 272–273 °C, ^1^H NMR (300 MHz, DMSO-d_6_, ppm) δ 6.78 (d, *J* = 8.22 Hz, 1H, phenyl-5), 7.13–7.16 (m, 2H, phenyl-6, thiazole-5), 7.27 (d, *J* = 2.10 Hz, 1H, phenyl-2), 8.11 (d, *J* = 6.78, 2H, pyridine-3,5), 8.17 (s, 1H, **H–C**=N), 8.82 (d, *J* = 6.72, 2H, pyridine-2,6), 13.16 (brs, 1H, C=**N–H**). ^13^C NMR (300 MHz, DMSO-d_6_, ppm) δ 103.05, 113.84, 116.19, 117.51, 122.67, 126.38, 135.59, 142.76, 143.18, 145.71, 145.98, 150.27, 157.43, and 167.32. Compact mass spectrometry (CMS): (*m*/*z*): [M + 1]^+^ calculated: 313.1; found: 313.3.

#### 4-(3,4-Dichlorophenyl)-2-(2-(pyridin-4-ylmethylene)hydrazinyl)thiazole (**2o**)

Yield: 85%, mp 301–302 °C, ^1^H NMR (300 MHz, DMSO-d_6_, ppm) δ 7.69 (d, *J* = 8.47 Hz, 1H, phenyl-5), 7.75 (s, 1H, thiazole-5), 7.86 (dd, *J* = 8.44, 2.07 Hz, 1H, phenyl-6), 8.10 (d, *J* = 2.04 Hz, 1H, phenyl-2), 8.12 (d, *J* = 6.81 Hz, 2H, pyridine-3,5), 8.15 (s, 1H, **H–C**=N), 8.84 (d, *J* = 6.72 Hz, 2H, pyridine-2,6), and 13.22 (brs, 1H, C=N–**NH**−). ^13^C NMR (300 MHz, DMSO-d_6_, ppm) δ 108.39, 122.78, 126.09, 127.69, 130.50, 131.47, 131.99, 135.21, 136.29, 143.30, 148.77, 149.90, and 167.85. Compact mass spectrometry (CMS) (*m*/*z*): [M + 1]^+^ calculated: 348.0; found: 349.2. HRMS (*m*/*z*): [M + 1]^+^ calculated: 349.0076; found: 349.0090.

### ADME Predictions

Key physicochemical properties related to the absorption, distribution, metabolism, and excretion (ADME) of the compounds were predicted in silico using the SwissADME tool.^35−37^

### Anticancer Activity

#### MTT Assay

The human lung cancer cell line A549 and mouse fibroblast cell line L929 were used to evaluate the cytotoxicity of the compounds. Cells propagated under the specified conditions were cultured in 96-well plates at 5 × 10^3^ cells/well. Compounds and cisplatin were applied at varying concentrations, and after incubation, 20 μL of phosphate buffer and 5 mg/mL MTT dye were added, followed by further incubation for 2 h. At the end of the incubation period, cell viability was determined by measuring the absorbance of the purple color formed by the breakdown of the MTT dye at 540 nm. Half-maximal inhibitory concentrations (IC_50_) were determined based on the tested concentrations of the compounds. The tests were triplicated.^38^ The MTT assay for cytotoxicity of compounds was determined according to previously published procedures.^39,40^

#### Apoptosis Detection

The compounds **2a**, **2b**, **2c**, **2j**, **2m,** and **2o**, identified as cytotoxically active, were tested using a FITC Annexin V apoptosis kit in a BD FACSAria flow cytometer device to determine the apoptosis and necrosis rates they induced. The compounds were treated with A549 cells at IC_50_ doses, and early and late apoptosis, necrosis, and viable cell ratios were determined as percentages. Cells were seeded at 37 °C in humidified air containing 5% CO_2_ with 10^5^ cells/plate in each well. A549 cells were collected, washed twice with ice-cold PBS, and resuspended in 100 μL of binding buffer. Five μL (5 μg/mL) of Annexin V-FITC and PI were added to the cells and incubated for 15 min in the dark at room temperature (20–25 °C). Then, 400 μL of binding buffer was added to the mixture samples and analyzed on a flow cytometer. Quadrant analyses were also presented.^41^ Compounds **2a**, **2b**, **2c**, **2j**, **2m**, and **2o**, with a satisfactory cytotoxic profile, were examined via flow cytometry to assess the apoptosis/necrosis cell ratio using previously published procedures.^42^

#### Mitochondrial Membrane Potential Measurement

Cell staining with JC-1 was performed according to the manufacturer’s recommendations for the BD , Pharmingen Flow Cytometry Kit. Alterations in mitochondrial membrane potential caused by the most active compounds—**2a**, **2b**, **2c**, **2j**, **2m**, and **2o**—were assessed in A549 cells. Compounds were applied at IC_50_ doses of no more than 1 × 10^6^/mL per well in 6-well plates. After treatment, each cell suspension was transferred to a 15 mL polystyrene centrifuge tube, the cells were centrifuged at 400*g* for 5 min, and the supernatant was removed. 0.5 mL of freshly prepared working solution was added to each pellet, and the solution was vortexed. The test cells were soaked in JC-1 working solution at 37 °C for 10–15 min, and the cells were washed twice. After centrifugation at 400*g* for 5 min, each cell pellet was suspended in 0.5 mL of 1X assay buffer and vortexed. The cells were then analyzed by flow cytometer. Cisplatin was used as a standard control.^43^ The analysis of mitochondrial membrane potential by flow cytometry for compounds **2a**, **2b**, **2c**, **2j**, **2m**, and **2o**, and cisplatin was performed according to the previously published procedures.^39,40^

#### Caspase-3 Activation

A Spectrofluorometric Caspase-3 assay kit (BD Pharmingen, Franklin Lakes, NJ) was used for determining Caspase-3 activation by measuring the caspase-3 or DEVD-cleaving activity. Initially, A549 cells at a concentration of 1 × 10^6^ cells/mL underwent a washing process using phosphate-buffered saline (PBS). Subsequently, they were suspended in a cold solution known as cell lysis buffer and kept on ice for a duration of 30 min. Following 24 h of incubation with different concentrations of test compounds and cisplatin, the cells were lysed to obtain cell lysates. In each reaction, a mixture of 5 mL of diluted Ac DEVD-AMC (a synthetic tetrapeptide fluorogenic substrate used to measure caspase-3 activity) and 0.2 mL of 1x HEPES buffer was added to a well. Then, 20 mL of cell lysate was introduced into each well/reaction. The reaction mixtures were incubated at 37 °C for 1 h. The amount of AMC released from Ac-DEVD-AMC was measured by using a microplate reader (PerkinElmer/Victor/X3) with an excitation wavelength of 380 nm and an emission wavelength of 460 nm. Apoptotic cell lysates containing active caspase-3 produced significant emissions compared to controls. In addition, the AMC emission of nonapoptotic control cell lysates was taken as 100%, and the emission of other cell lysates was measured relative to the emission of control cells. All experiments were repeated in duplicate. Active compounds, which are the most cytotoxic compounds, were evaluated for caspase-3 activation using previously published procedures.^40,44^

#### Matrix Metalloproteinase-9 (MMP-9) Inhibition

MMP-9 inhibition was assessed using a colorimetric kit from Enzo Life Sciences Inc. (Farmingdale, NY, USA), following procedures similar to those described in previous studies.^25,26^ A thiopeptide was used as a chromogenic substrate (Ac-PLG-[2-mercapto-4-methyl-pentanoyl]-LG-OC_2_H_5_). 2-Nitro-5-thiobenzoic acid, formed by the reaction of the sulfhydryl group produced as a result of hydrolysis of the thioester by the MMP-9 enzyme and then reaction with DTNB [5,5′-dithiobis(2-nitrobenzoic acid), Ellman’s reagent], gives absorbance at 412 nm in a microplate reader (BioTek, PowerWave, Gen5 software, Winooski, VT, USA). Based on these absorptions, MMP-9 inhibition concentrations were determined. The assays were performed in triplicate. NNGH (*N*-isobutyl-*N*-(4-methoxyphenylsulfonyl)glycyl hydroxamic acid) was used as a control inhibitor. Data were shown as mean ± SD. The inhibitor % remaining activity of MMPs was calculated using the following equation:

Inhibitor% activity remaining = (*V* inhibitor/*V* control) × 100.

The inhibition (percent) of MMPs was calculated using the following equation:
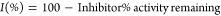


### Statistical Analyses

The concentration that inhibits 50% of the cell population was calculated as IC_50_ using GraphPad Prism 9.0 statistical software program, and standard deviation values were analyzed. Data are expressed as mean ± SD. Comparisons were made using one-way ANOVA for continuous variables with normal distribution, and Tukey’s test was used for post hoc analysis of group differences. *p* < 0.05 was considered statistically significant.

The apoptotic results (Annexin V-FITC, Caspase 3, JC-1) were evaluated by flow cytometry using FACS Diva Version 6.1.1. software, and the apoptotic cells were determined as the percentage of cells.

The cell viability value of the control group was considered 100%, and all viability values were calculated using the formula below. The experiment was performed in 3 replicates.
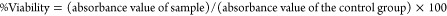


### *In Silico* Calculations

#### Molecular Docking and Molecular Dynamics Studies

Molecular docking studies were conducted to elucidate the binding modes of active compounds within the active sites of the MMP-9 enzyme complex (PDB ID: 5I12), retrieved from the Protein Data Bank (www.pdb.org, accessed April 07, 2020). Schrödinger’s Maestro interface and its applications (LigPrep,^45^ and Glide^46^ modules) were used for the molecular docking study. Using the docking pose, a molecular dynamics simulation study was performed according to the Maestro Desmond interface program.^47^ The molecular dynamics simulation (MDS) was carried out at 100 ns. The stability analysis of the identified hits was conducted. Preparing the system setup, performing molecular dynamics simulations, and computing the interaction analysis were carried out according to similar procedures.^30,31,37^ All systems were set up using “System Builder” module. Using this module, the complex structure was subjected to energy minimization (OPLS3e standard force field). The transferable intermolecular potential with the three-point water (TIP3P) model was utilized for the composition of the hydration model. The neutralization of the system was achieved by using Na^+^ and Cl^–^ ions. The molecular dynamics simulation study was performed following the completion of the system setup. The applied docking and dynamics simulation procedures were performed in the same manner as those described in previously published work.^25^

## Data Availability

Samples of the compounds **2a**–**2o** are available from the authors. The MDS video can be watched via this link (youtu.be/-n_N1qawRfA)

## Supporting Information Available

Characterization data (^1^H NMR, ^13^C NMR, MS, and APCI) for the synthesized compounds; this additional material provides further confirmation and details relevant to the experimental section of the manuscript (PDF)
