# Supplementary material for: Development and Biological Assessment of Thiazole-Based Pyridines for Targeted Therapy in Lung Cancer
Source: ACS Omega. 2025 Apr 23;10(17):17551–64. doi: 10.1021/acsomega.4c11252 (PMC12059926; doi:10.1021/acsomega.4c11252)
Supplement: Supplementary file 1 — ao4c11252_si_001.pdf [file ao4c11252_si_001.pdf]

Development and Biological Assessment of Thiazole-Based Pyridines for Targeted Therapy in Lung Cancer

Demokrat NUHA[a,b], Sam DAWBAA[a,c,d], Asaf Evrim EVREN [a,e,*], Zennure Şevval ÇİYANCI[f], Halide Edip TEMEL[f], Gülşen AKALIN ÇİFTÇİ[f], Leyla YURTTAŞ[a,*]

[a] Anadolu University, Faculty of Pharmacy, Department of Pharmaceutical Chemistry, 26470, Eskişehir, Turkey.

[b] University for Business and Technology, Faculty of Pharmacy, Lagjja Kalabria, 10000, Prishtina, Kosovo.

[c] Al-Hikma University, Faculty of Medical Sciences, Department of Pharmacy, Dhamar, Yemen.

[d] Thamar University, Faculty of Medical Sciences, Department of Doctor of Pharmacy (PharmD), Dhamar, Yemen.

[e] Bilecik Seyh Edebali University, Vocational School of Health Services, Pharmacy Services, Bilecik, Turkey.

[f] Anadolu University, Faculty of Pharmacy, Department of Biochemistry, 26470, Eskişehir, Turkey.

***Corresponding authors**

Asaf Evrim EVREN, asafevrimevren@anadolu.edu.tr, asafevrim.evren@bilecik.edu.tr

Leyla YURTTAŞ, lyurttas@anadolu.edu.tr

Supplemental Materials

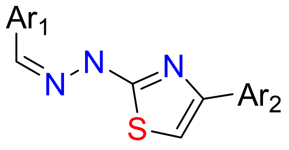

2a-2o

Figure S 1. The Structure of (pyridin-3-ylmethylene-hydrazinyl)thiazole derivatives (**2a–2o**).

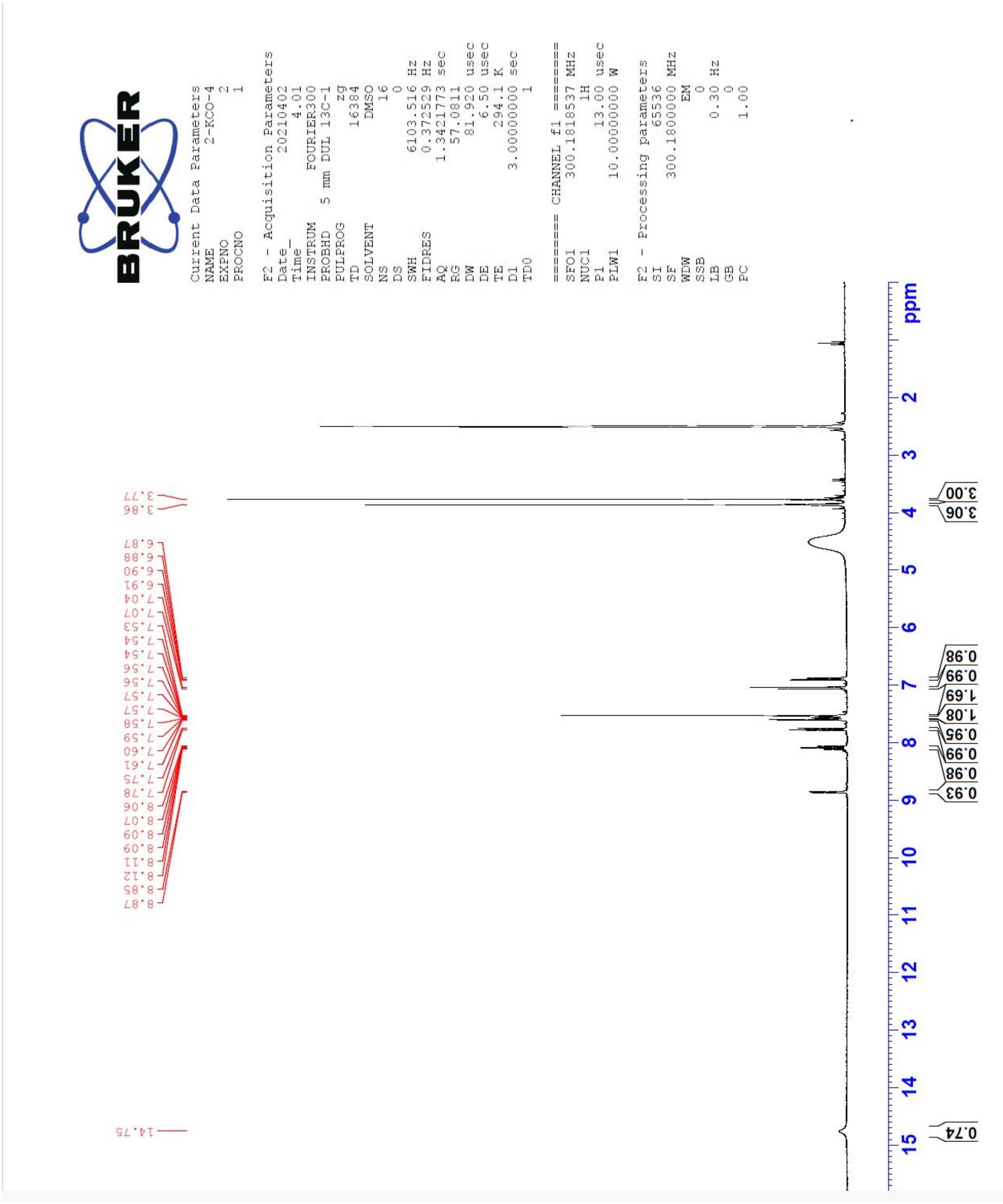

Figure S 2. 1H NMR of **2a**.

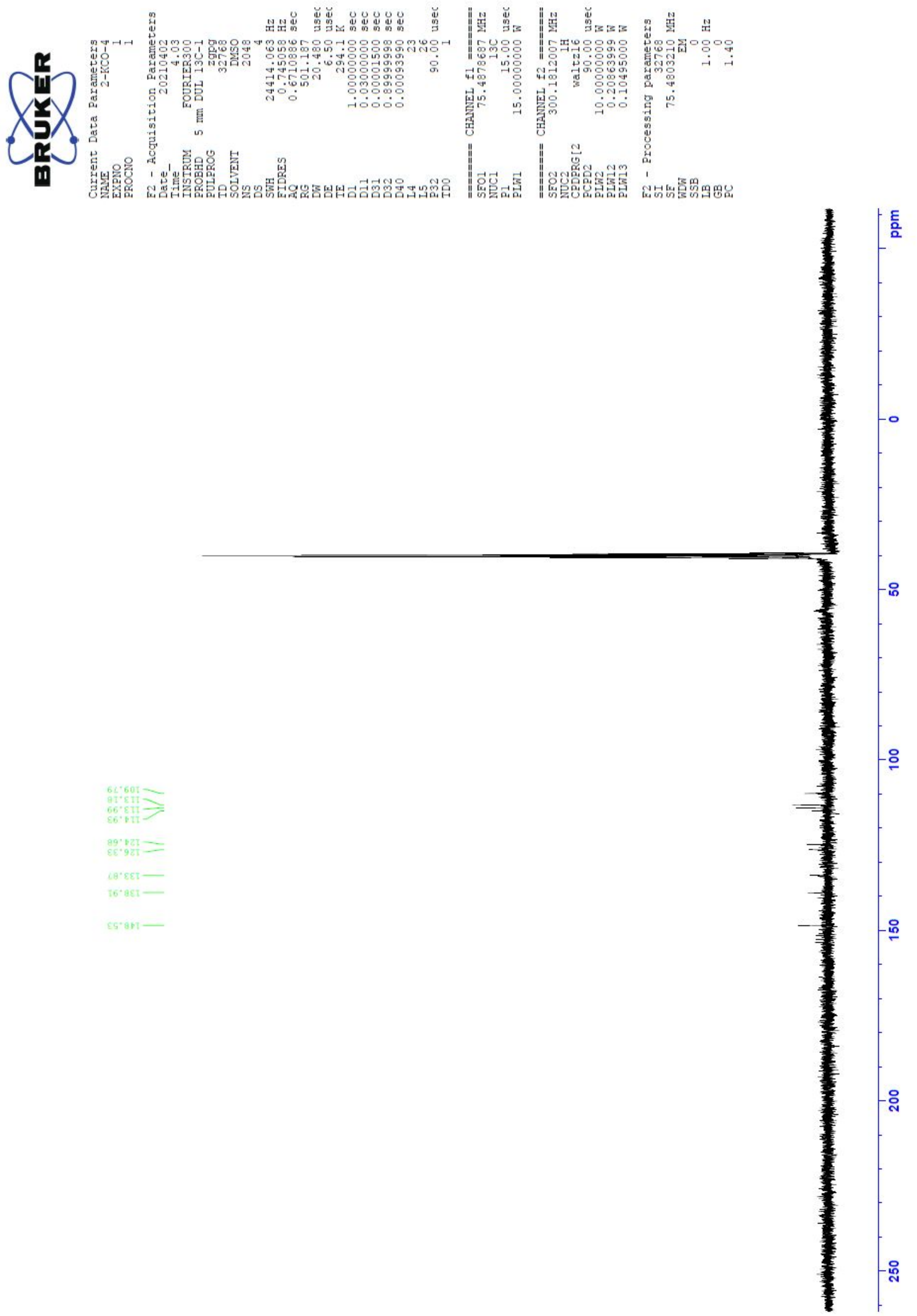

Figure S 3. 13C NMR of **2a**.

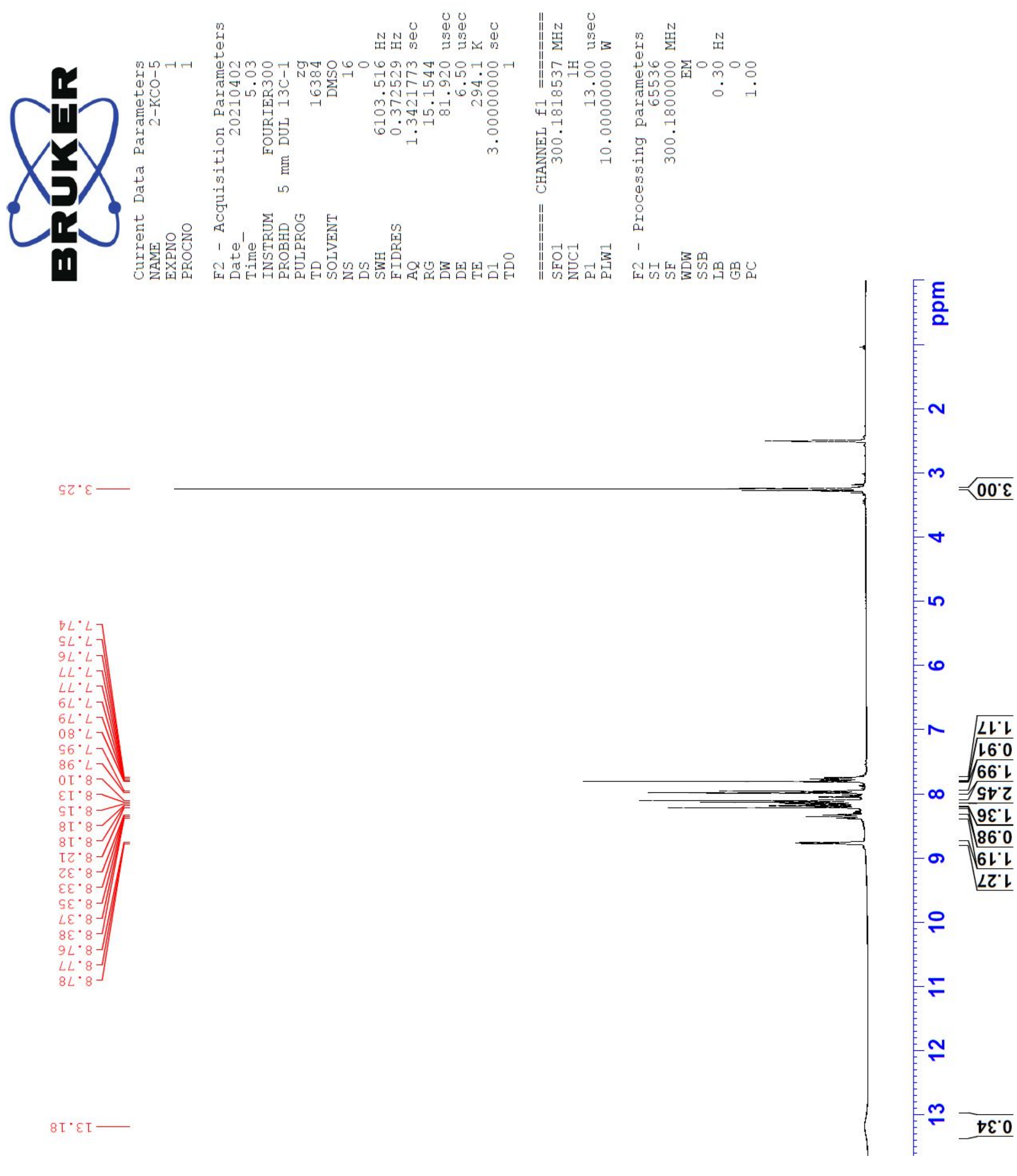

Figure S 4. 1H NMR of **2B**.

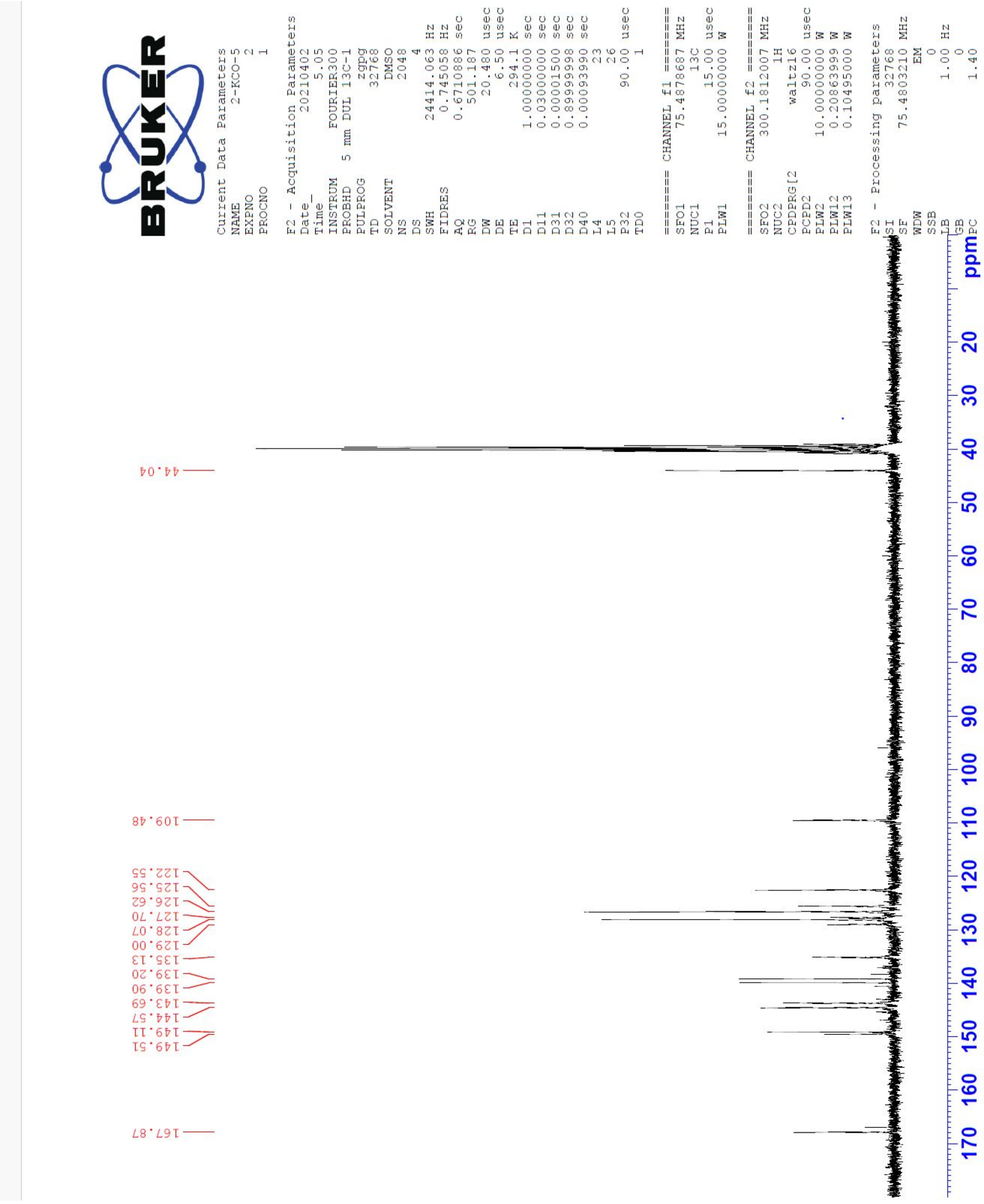

Figure S 5. 13C NMR of **2b**.

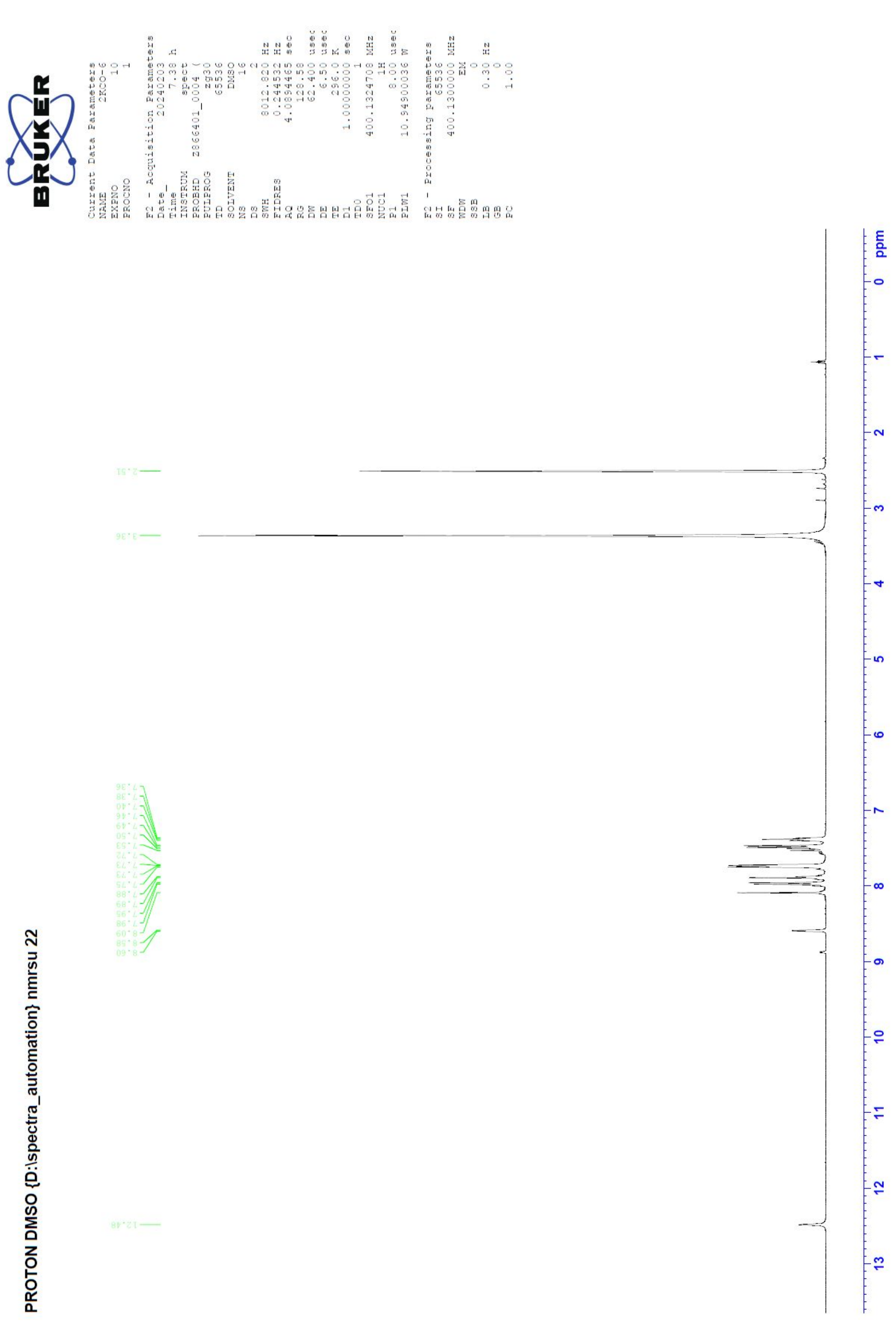

Figure S 6. 1H NMR of **2C**.

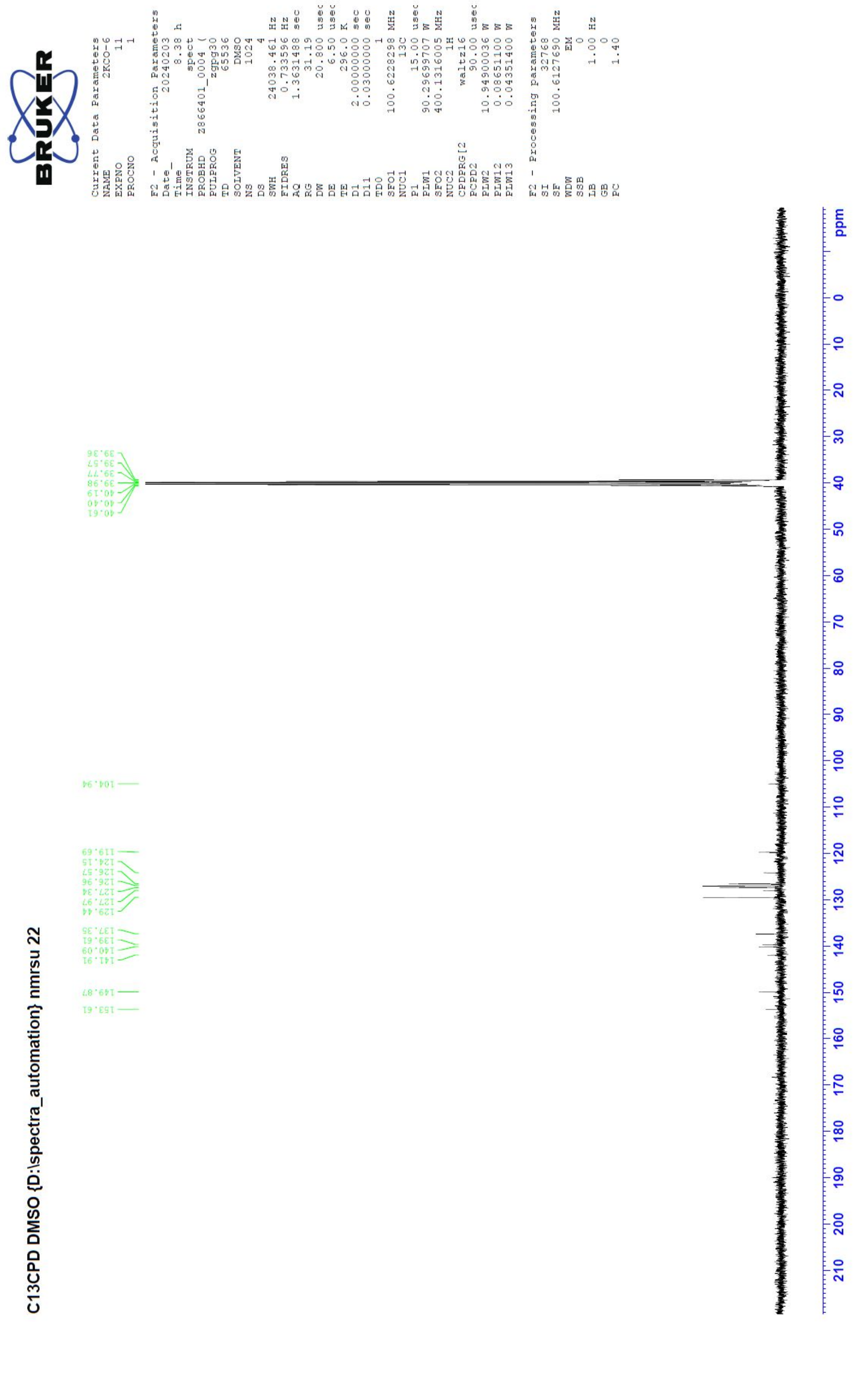

Figure S 7. 13C NMR of **2c**.

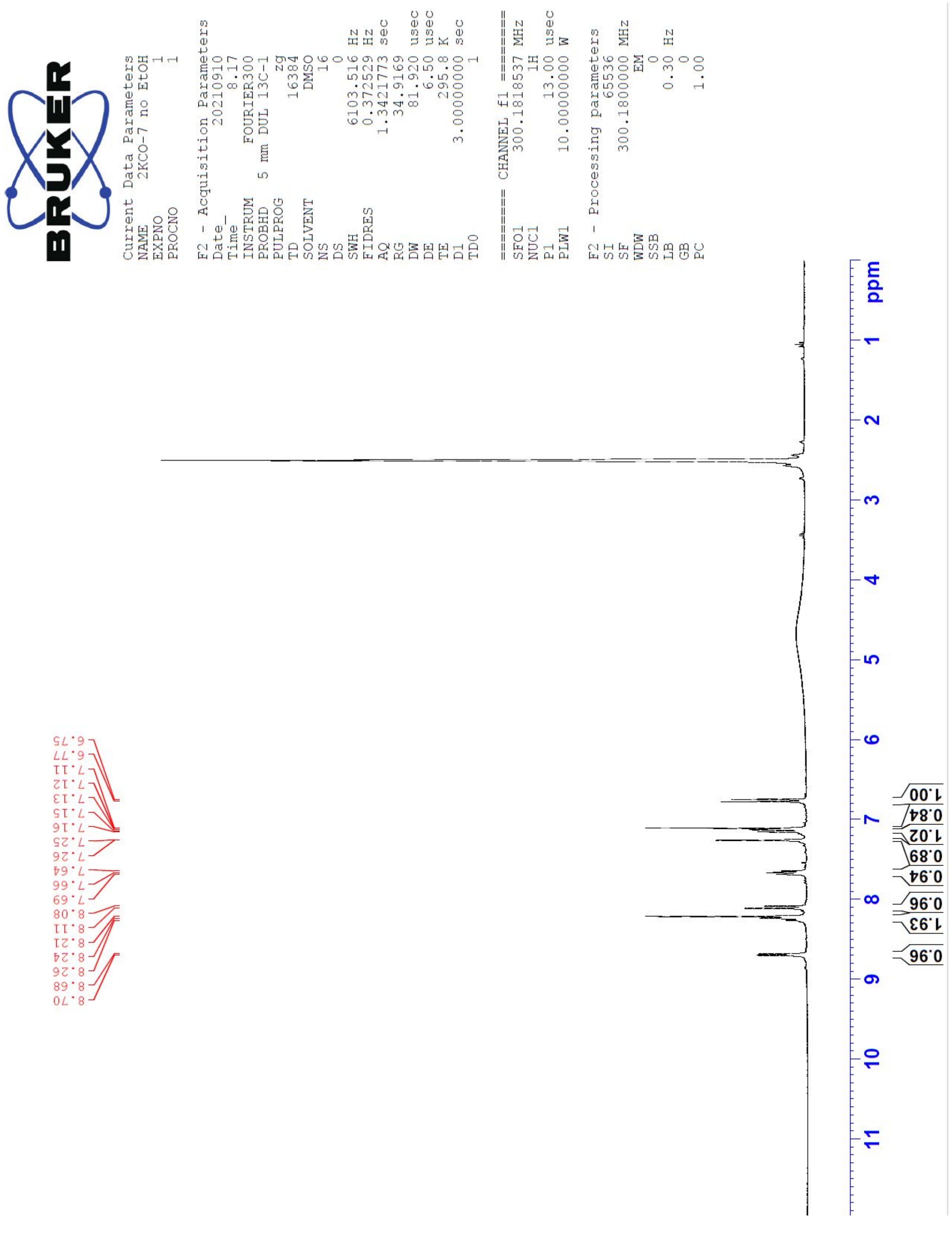

Figure S 8. 1H NMR of **2d**.

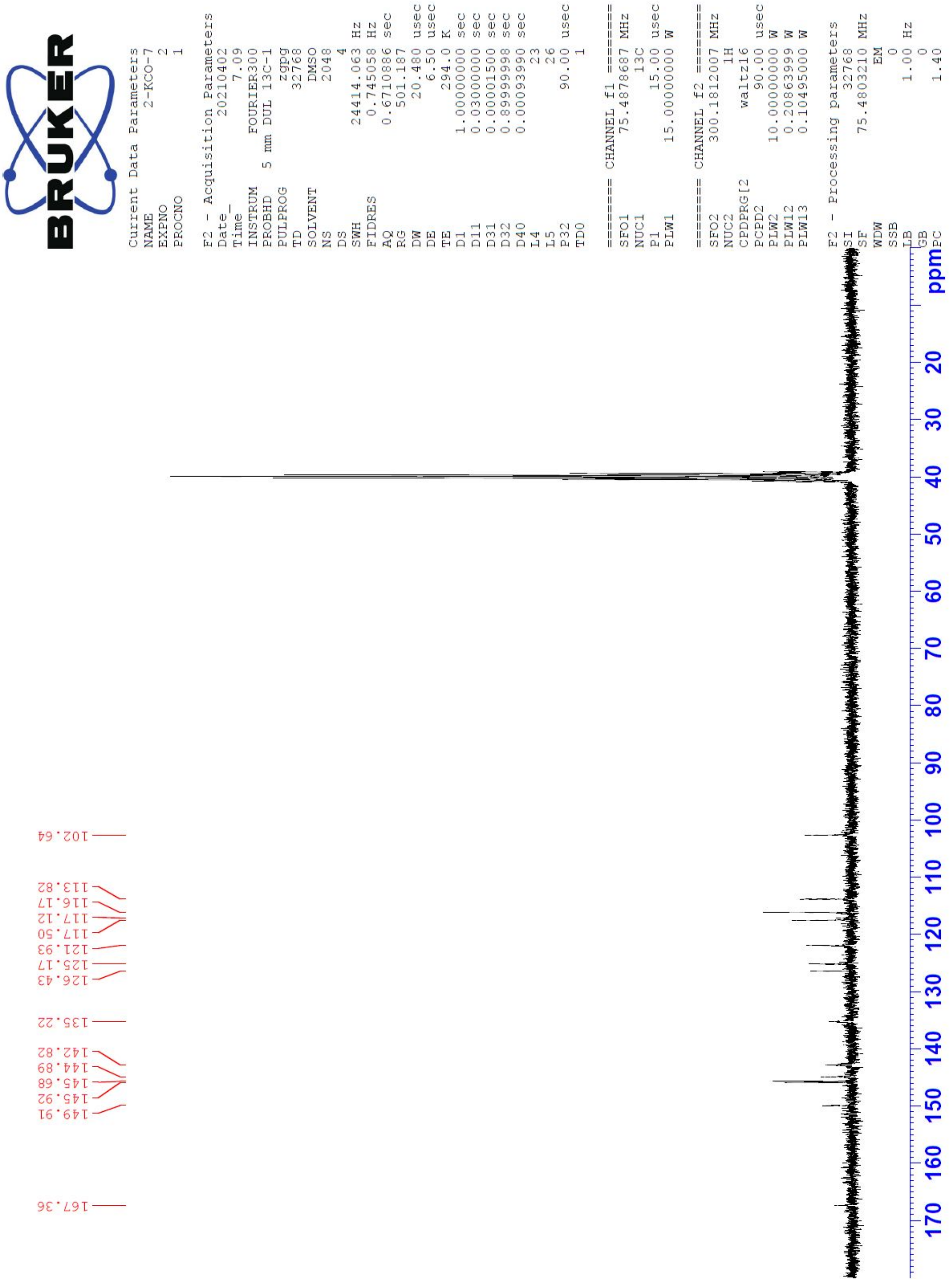

Figure S 9. 13C NMR of **2d**.

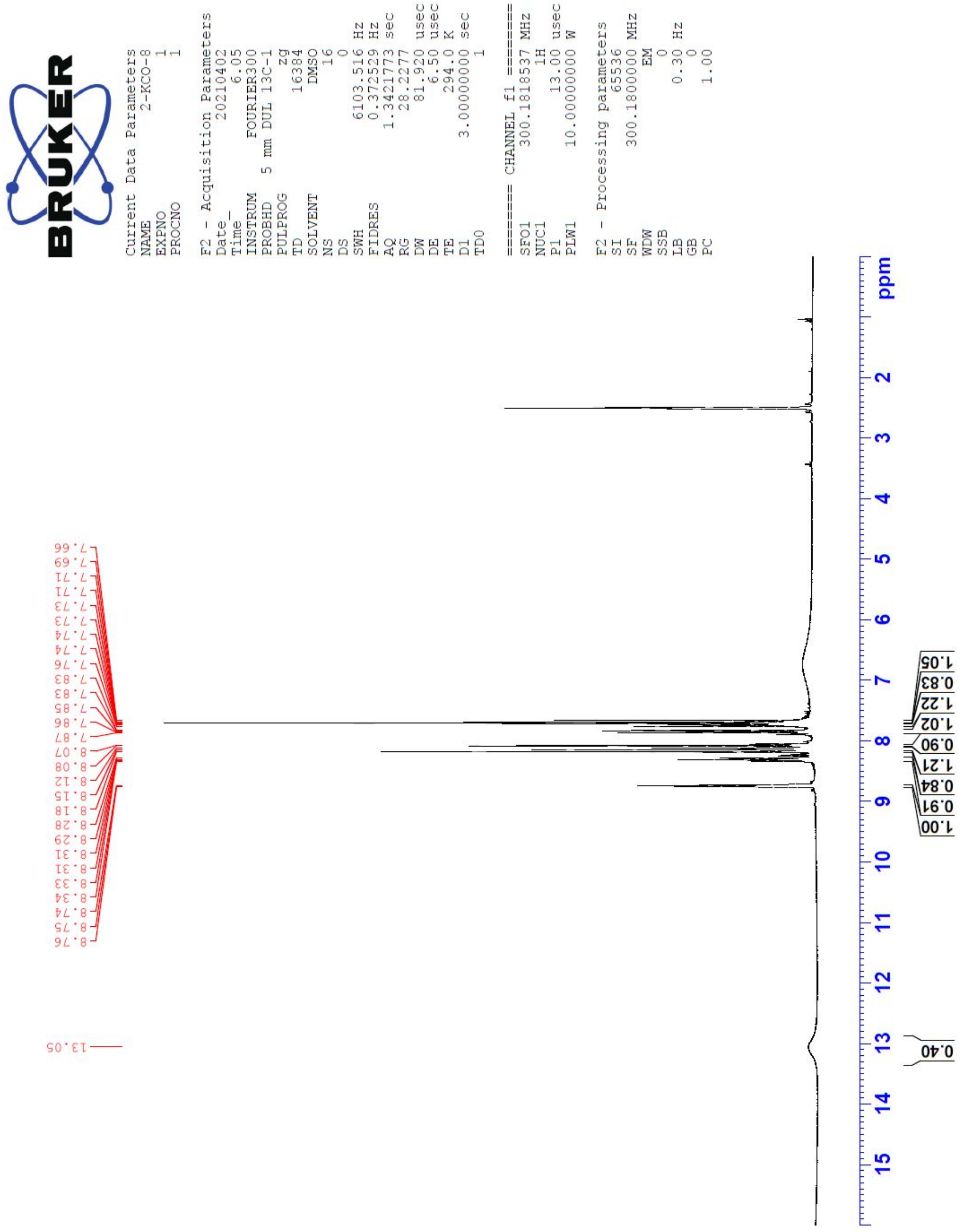

Figure S 10. 1H NMR of **2e**.

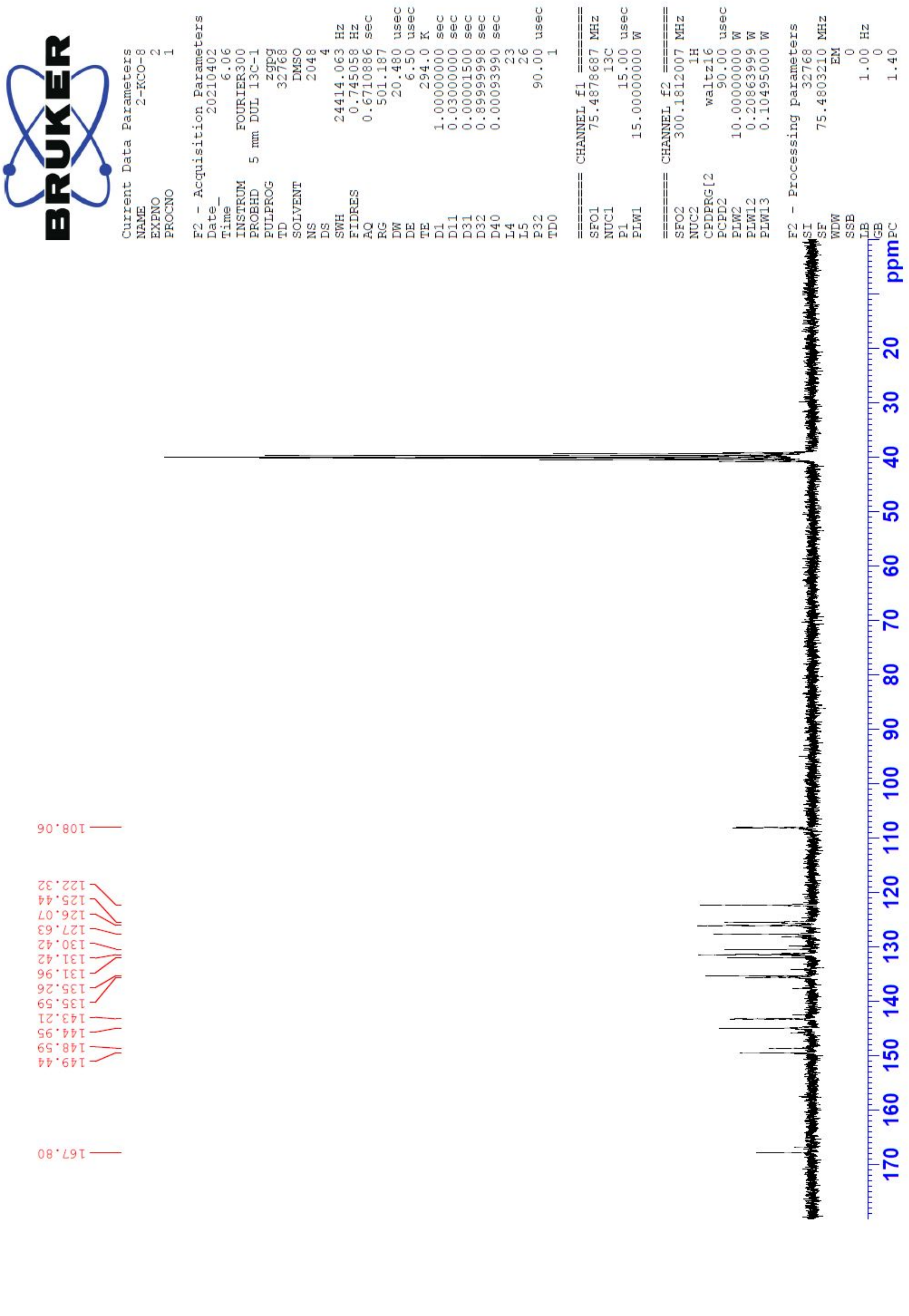

Figure S 11. 13C NMR of **2e**.

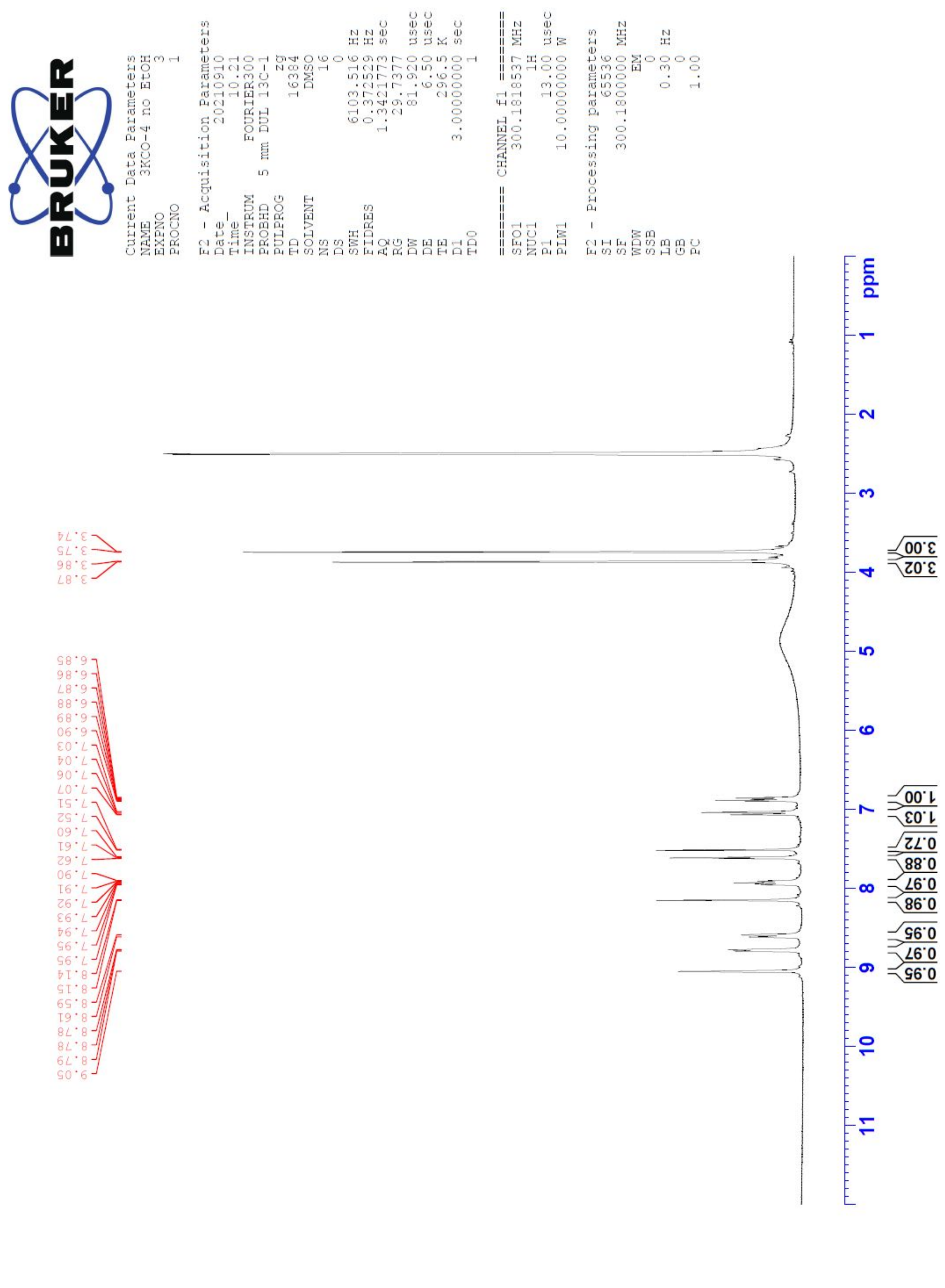

Figure S 12. 1H NMR of **2f**.

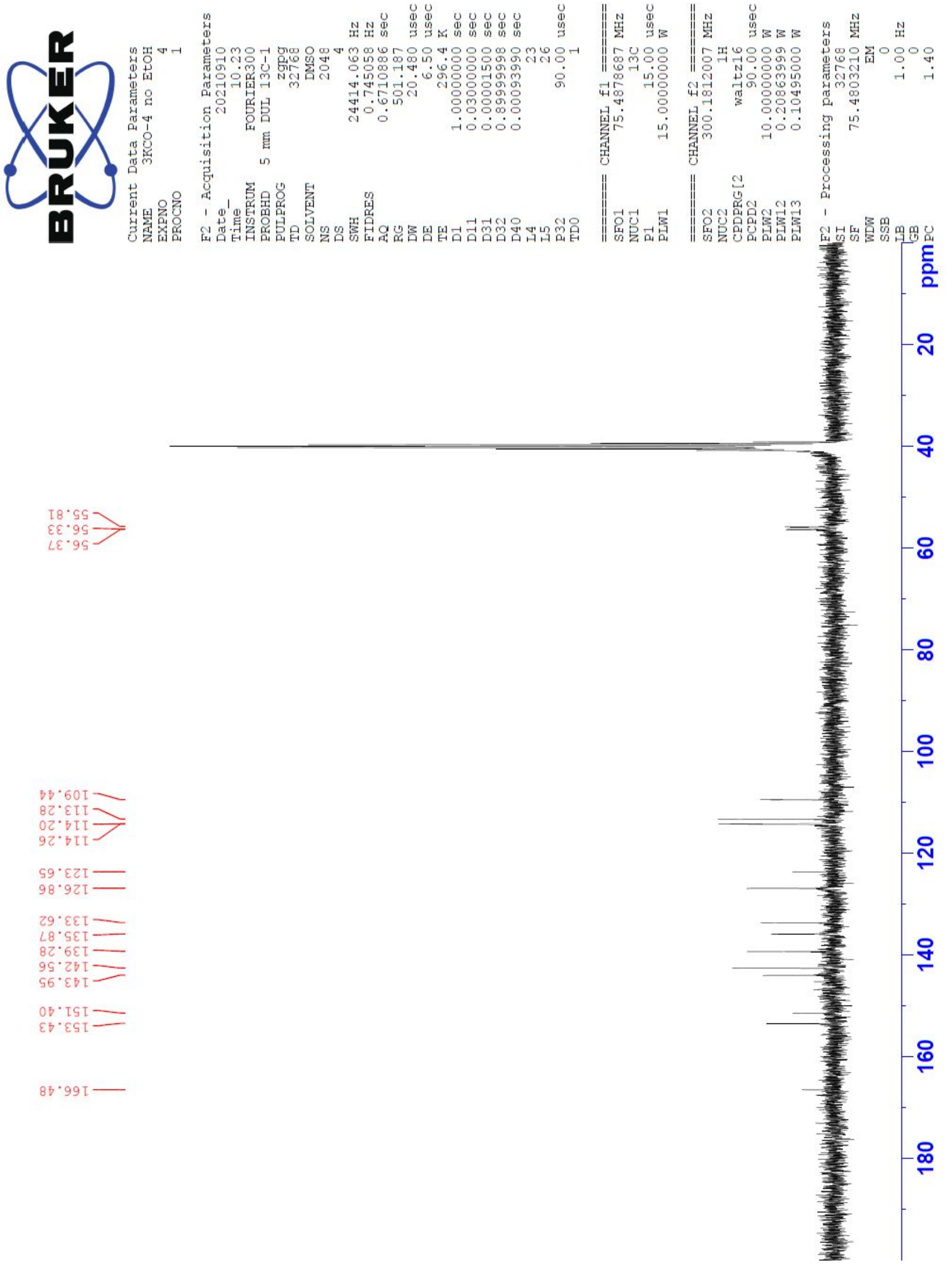

Figure S 13. 13C NMR of **2f**.

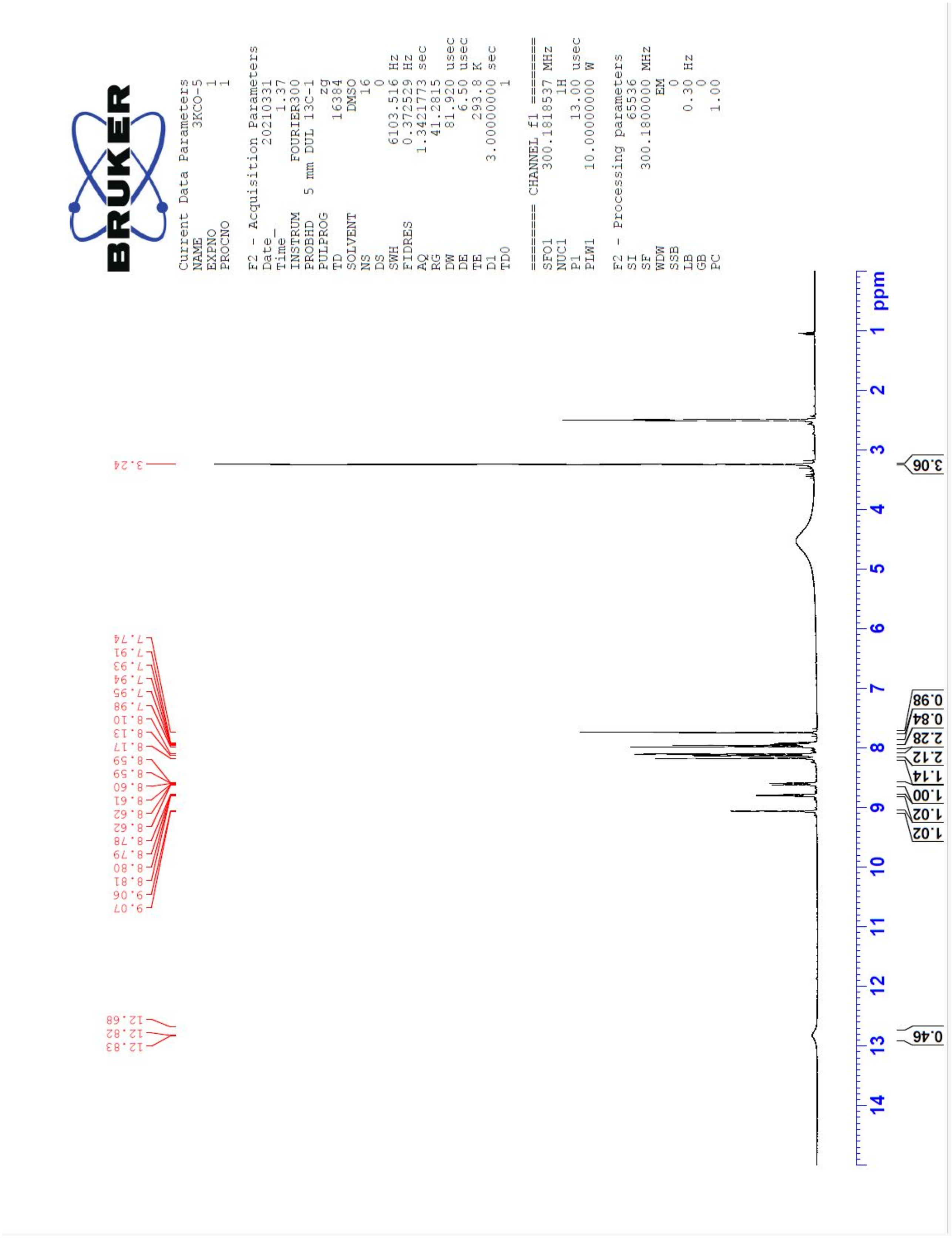

Figure S 14. 1H NMR of **2g**.

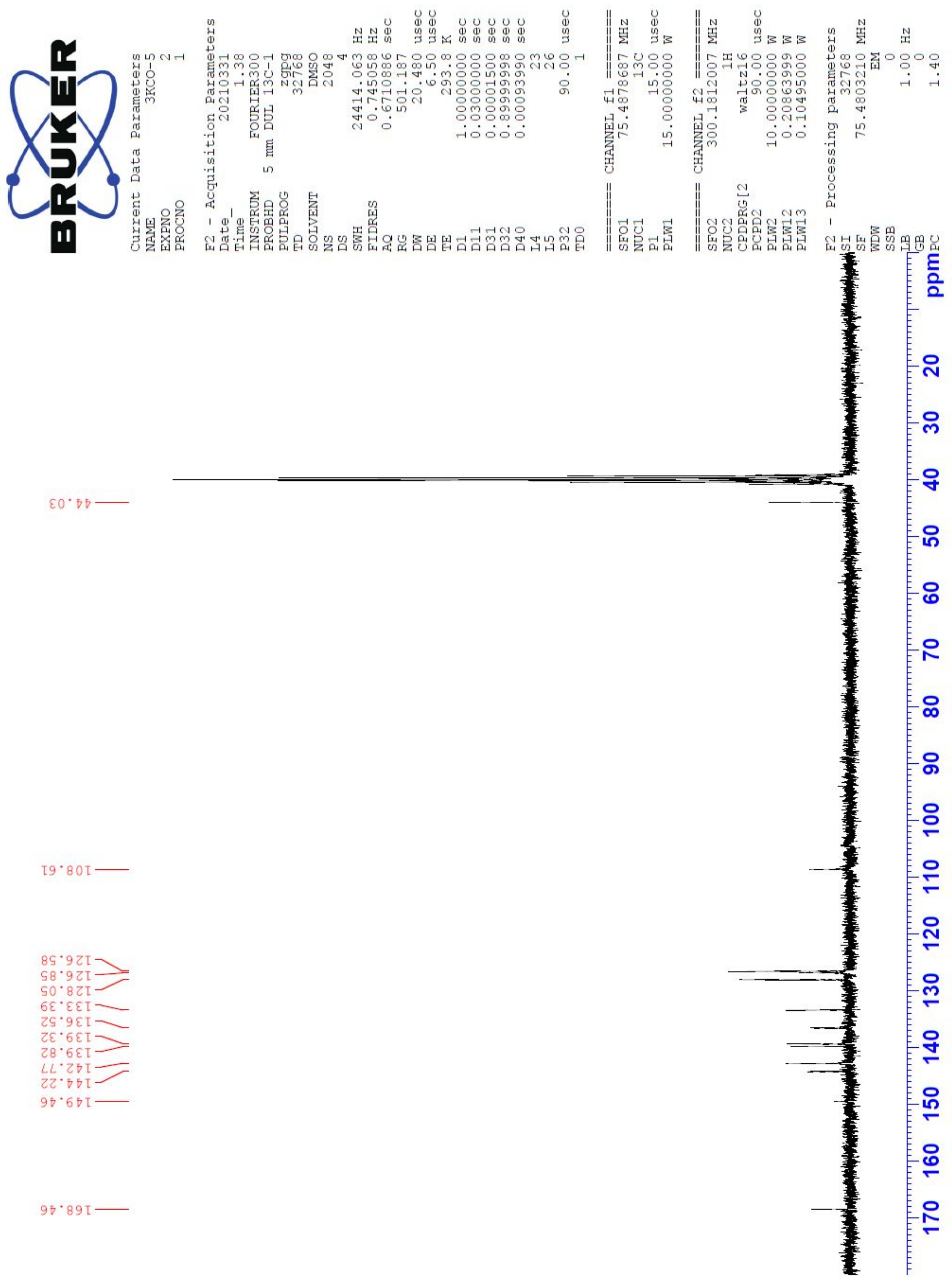

Figure S 15. 13C NMR of **2g**.

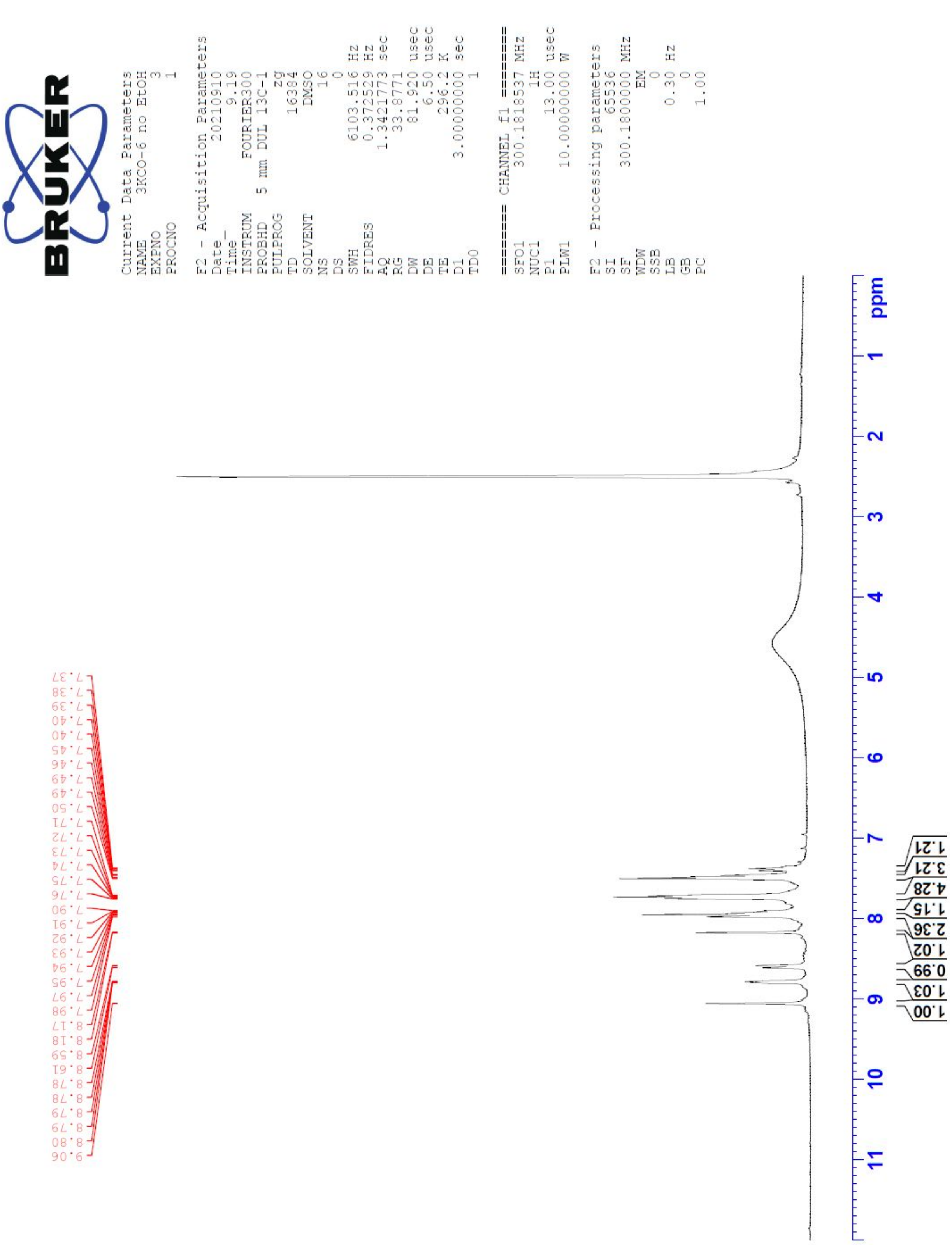

Figure S 16. 1H NMR of **2h**.

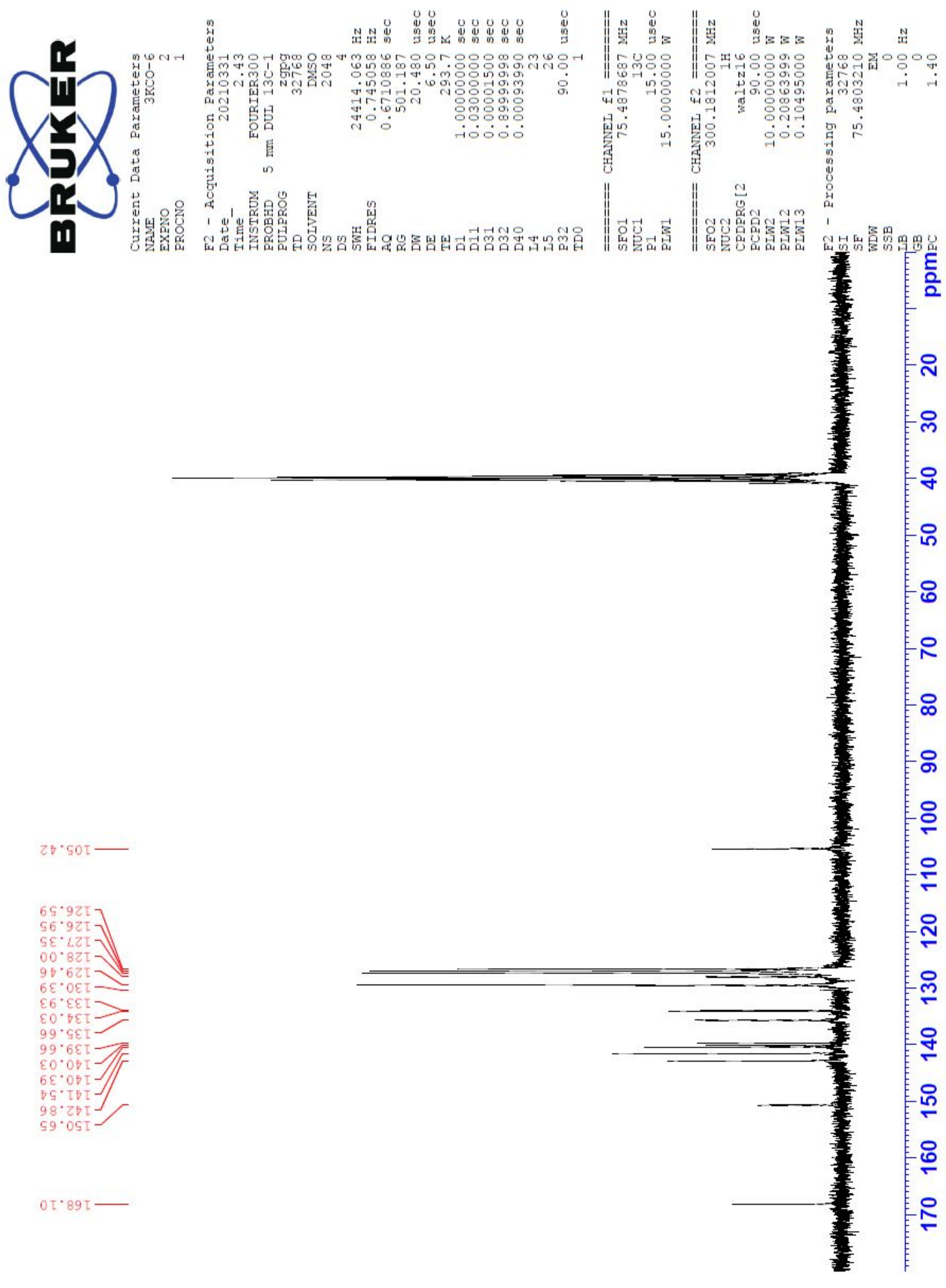

Figure S 17. 13C NMR of **2h**.

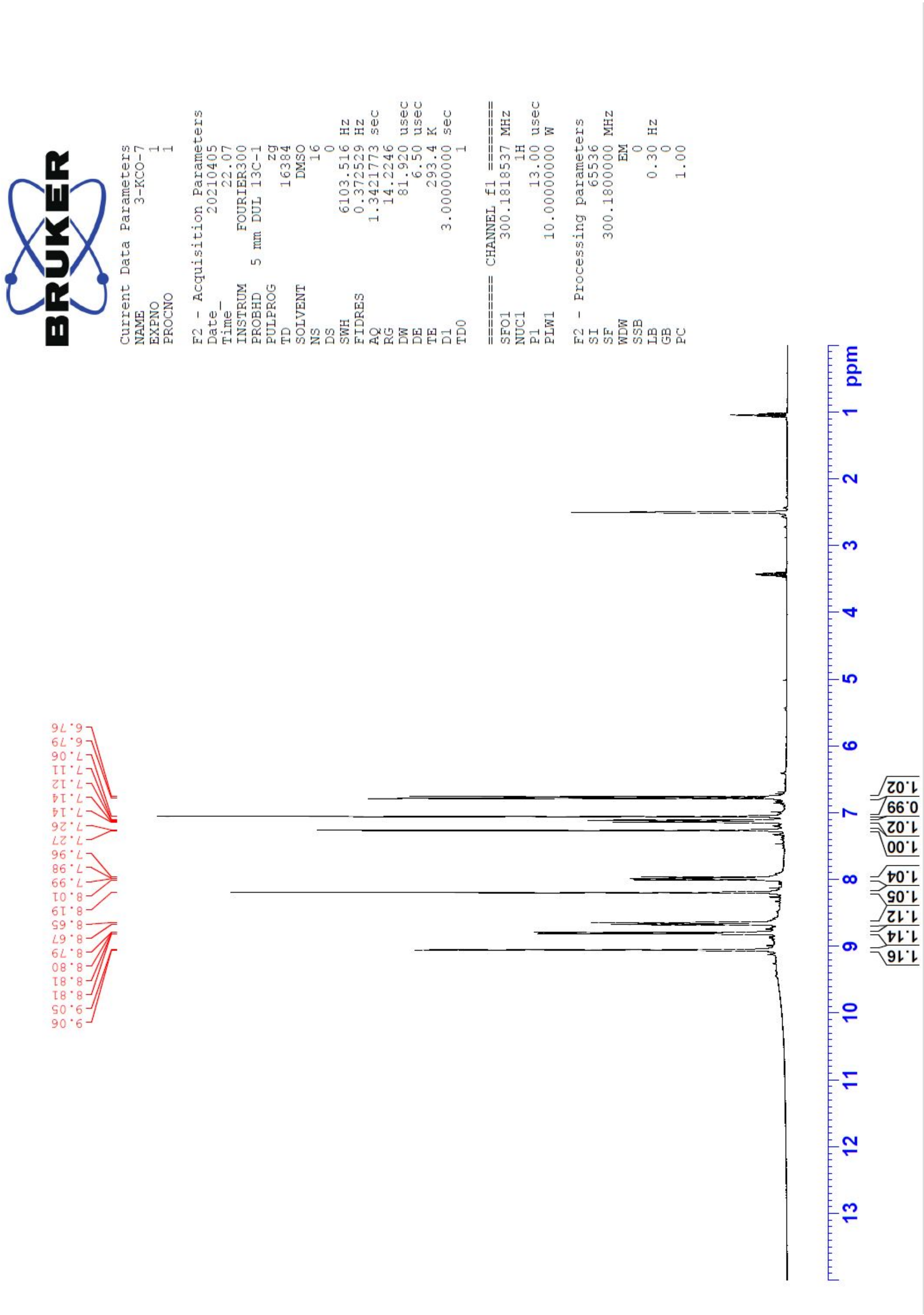

Figure S 18. 1H NMR of **2i**.

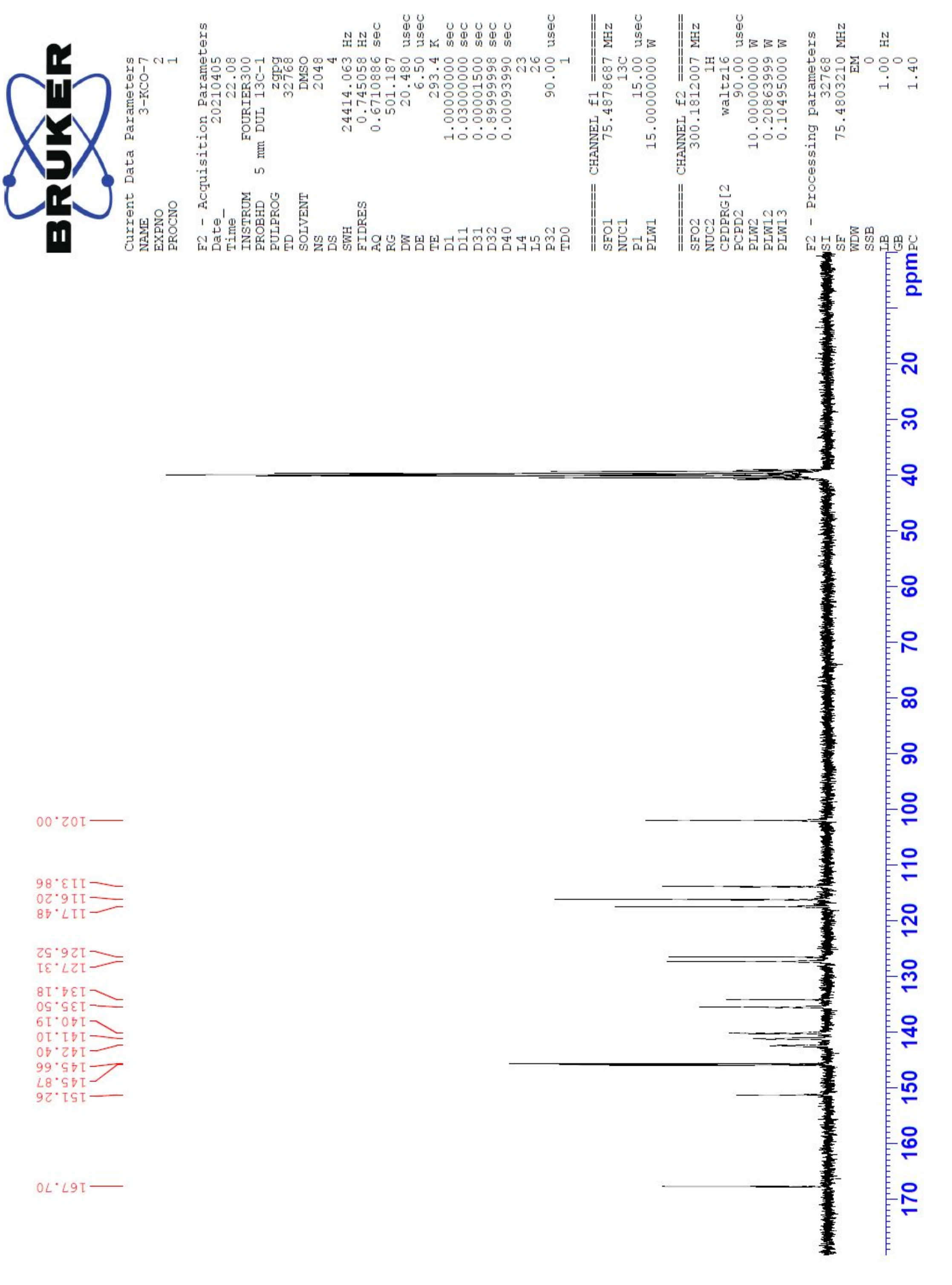

Figure S 19. 13C NMR of **2i**.

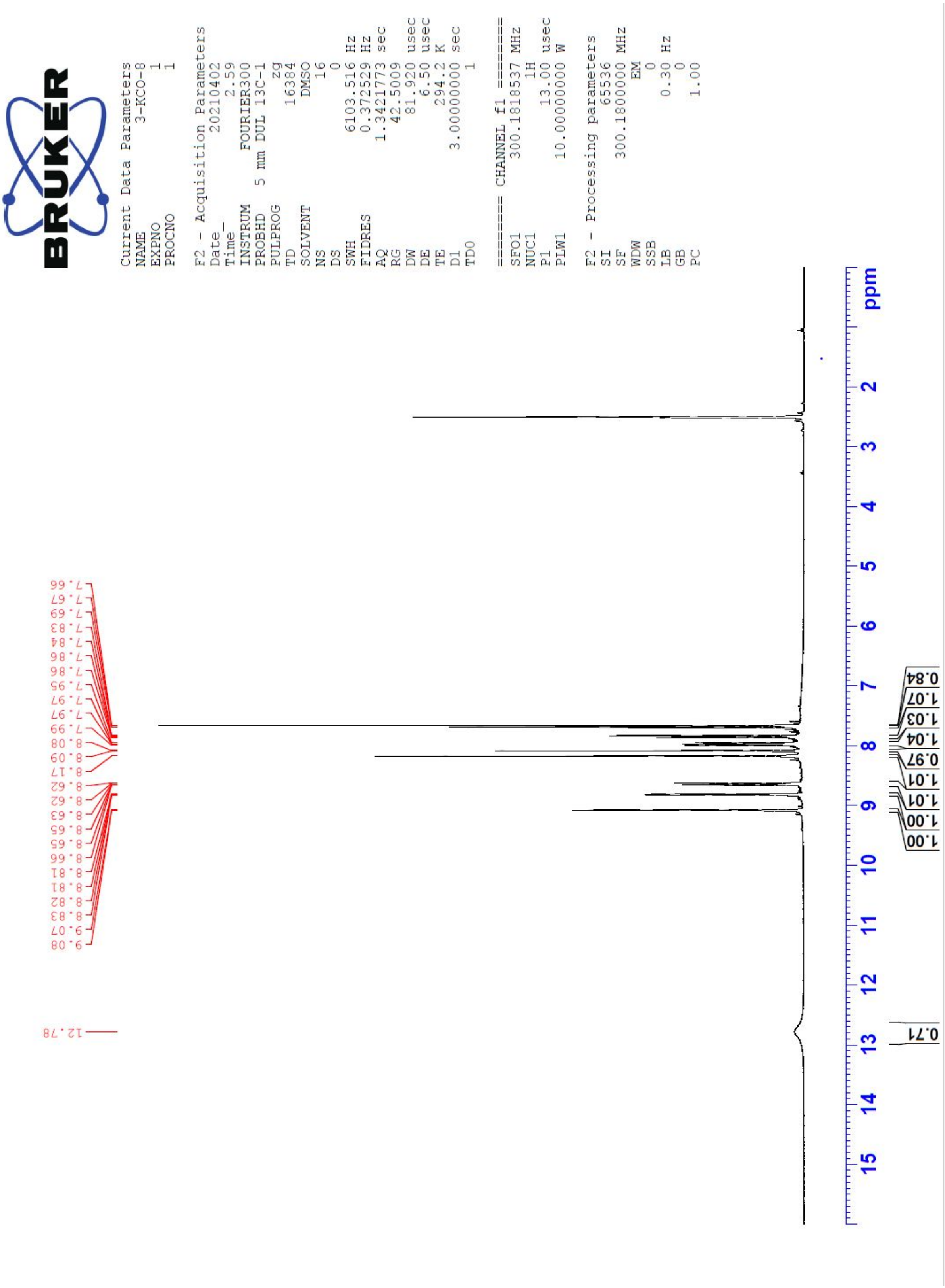

Figure S 20. 1H NMR of **2j**.

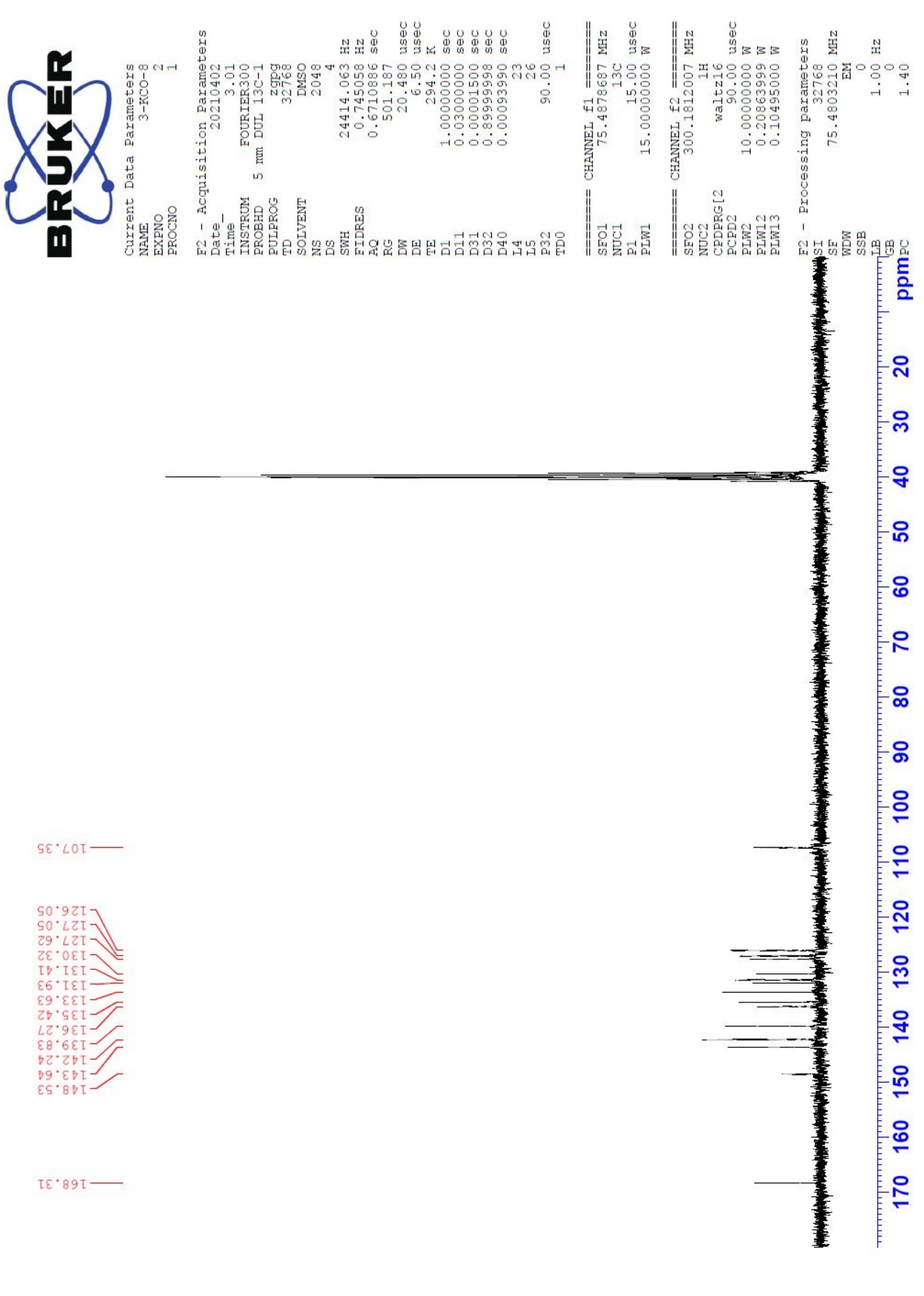

Figure S 21. 13C NMR of **2j**.

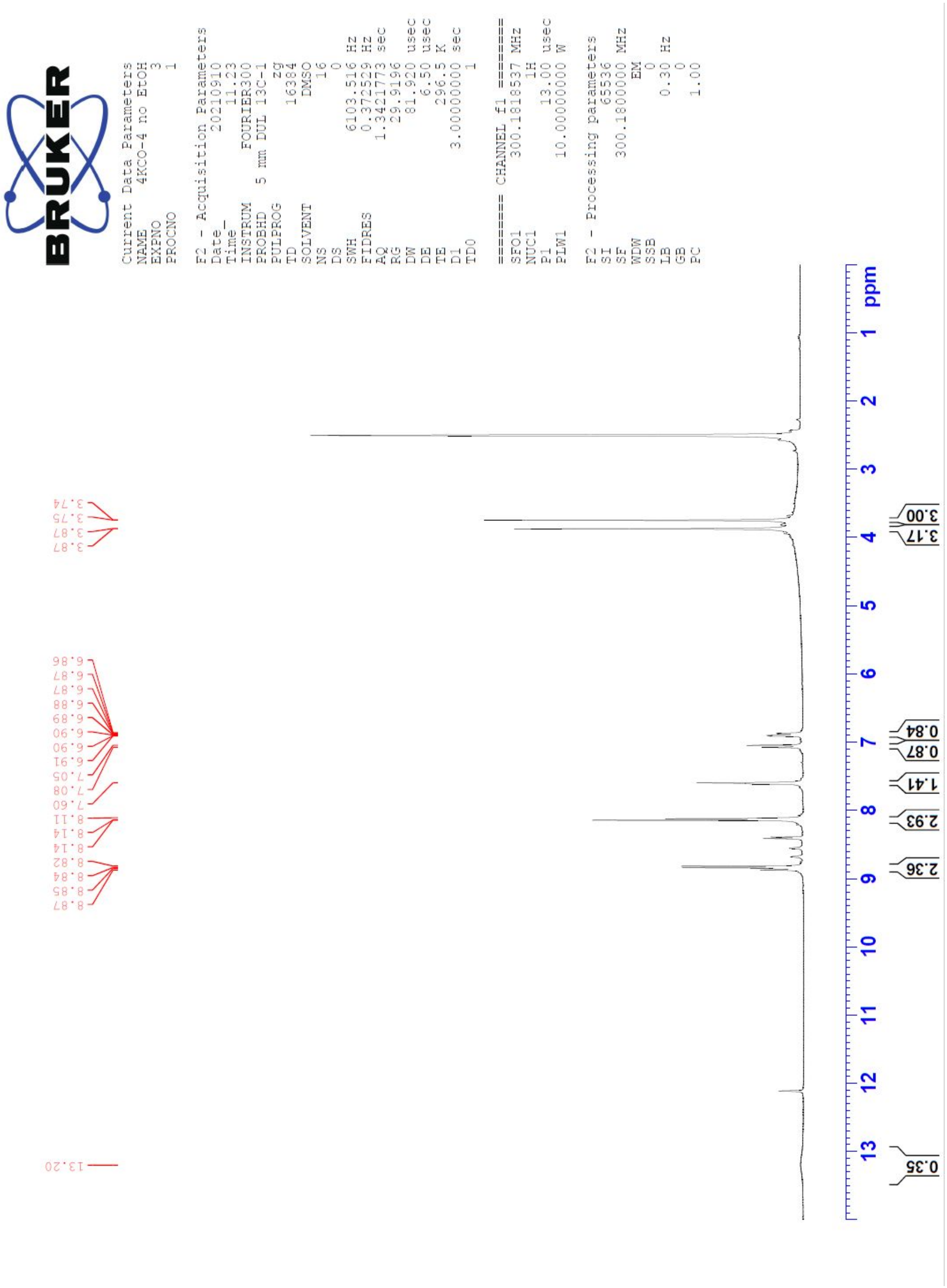

Figure S 22. 1H NMR of **2k**.

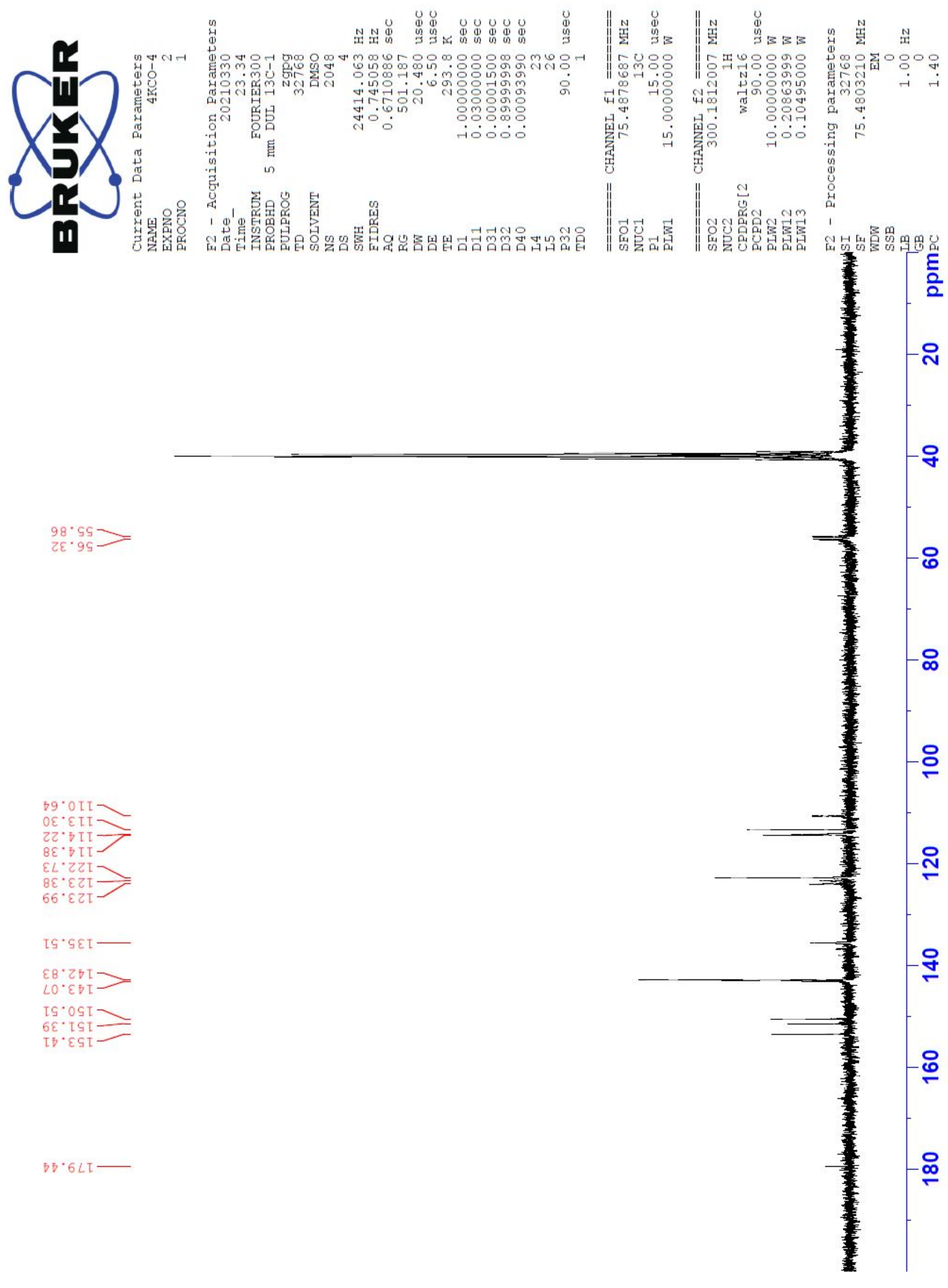

Figure S 23. 13C NMR of **2k**.

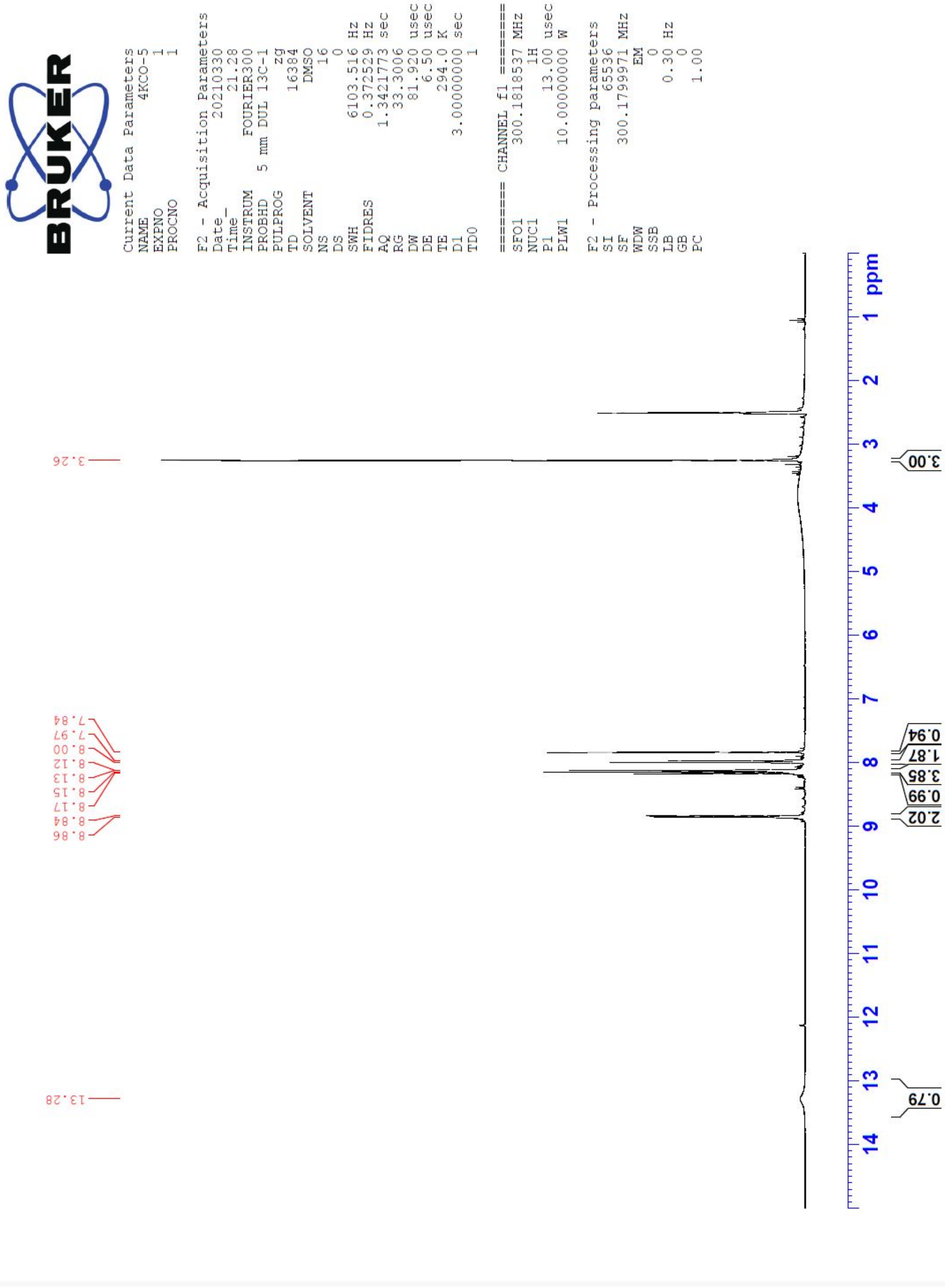

Figure S 24. 1H NMR of **2l**.

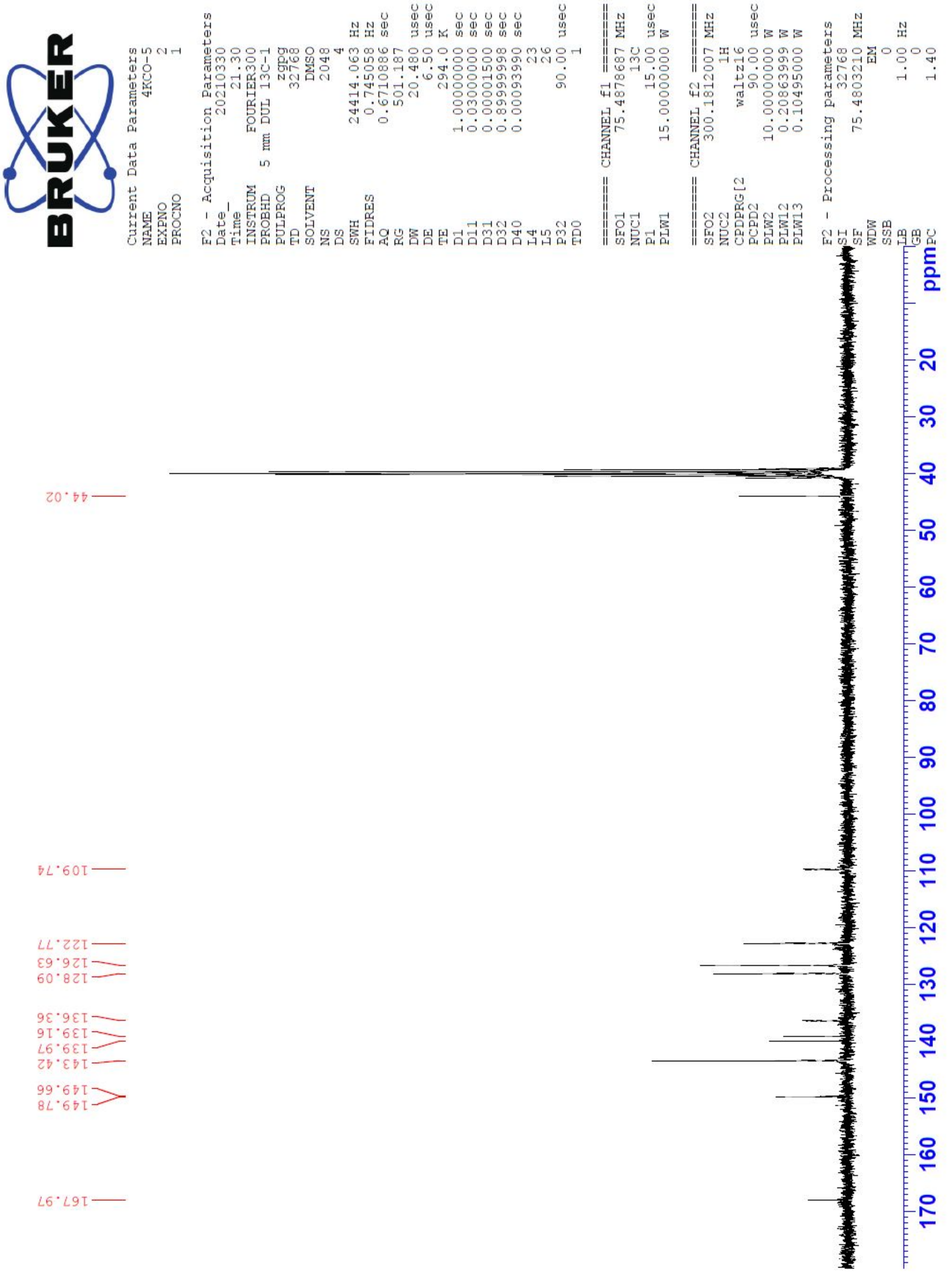

Figure S 25. 13C NMR of **2l**.

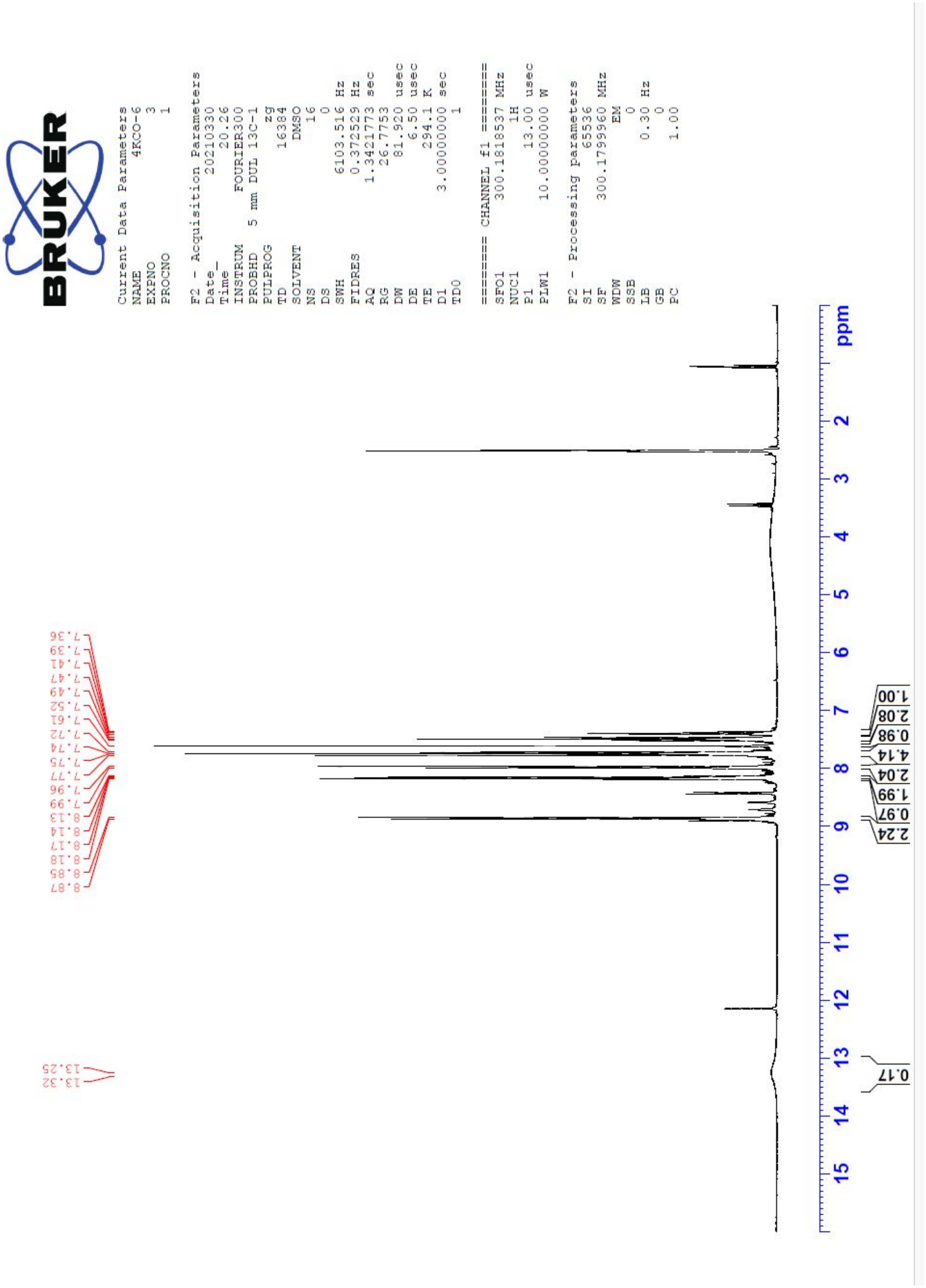

Figure S 26. 1H NMR of **2m**.

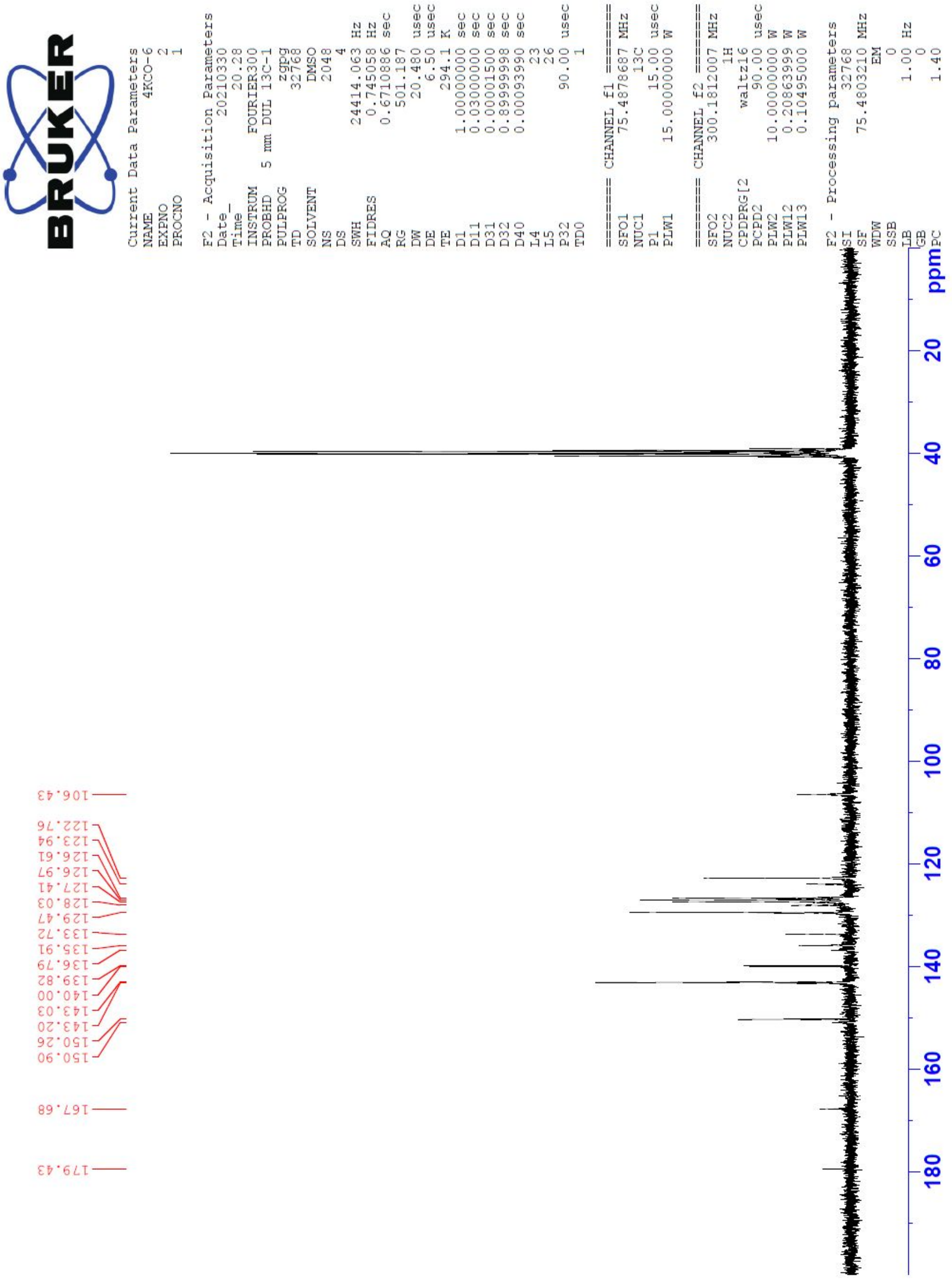

Figure S 27. 13C NMR of **2m**.

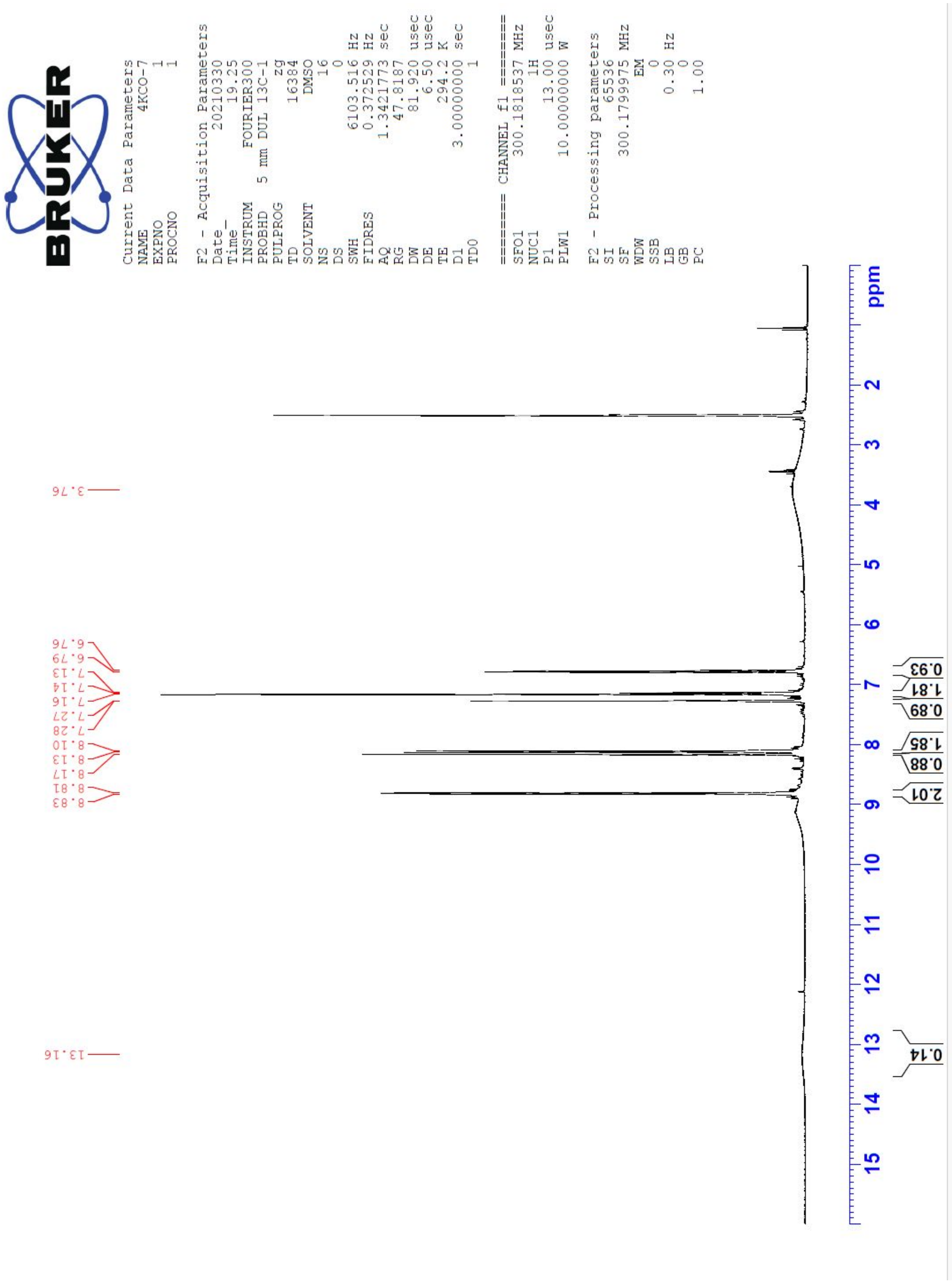

Figure S 28. 1H NMR of **2n**.

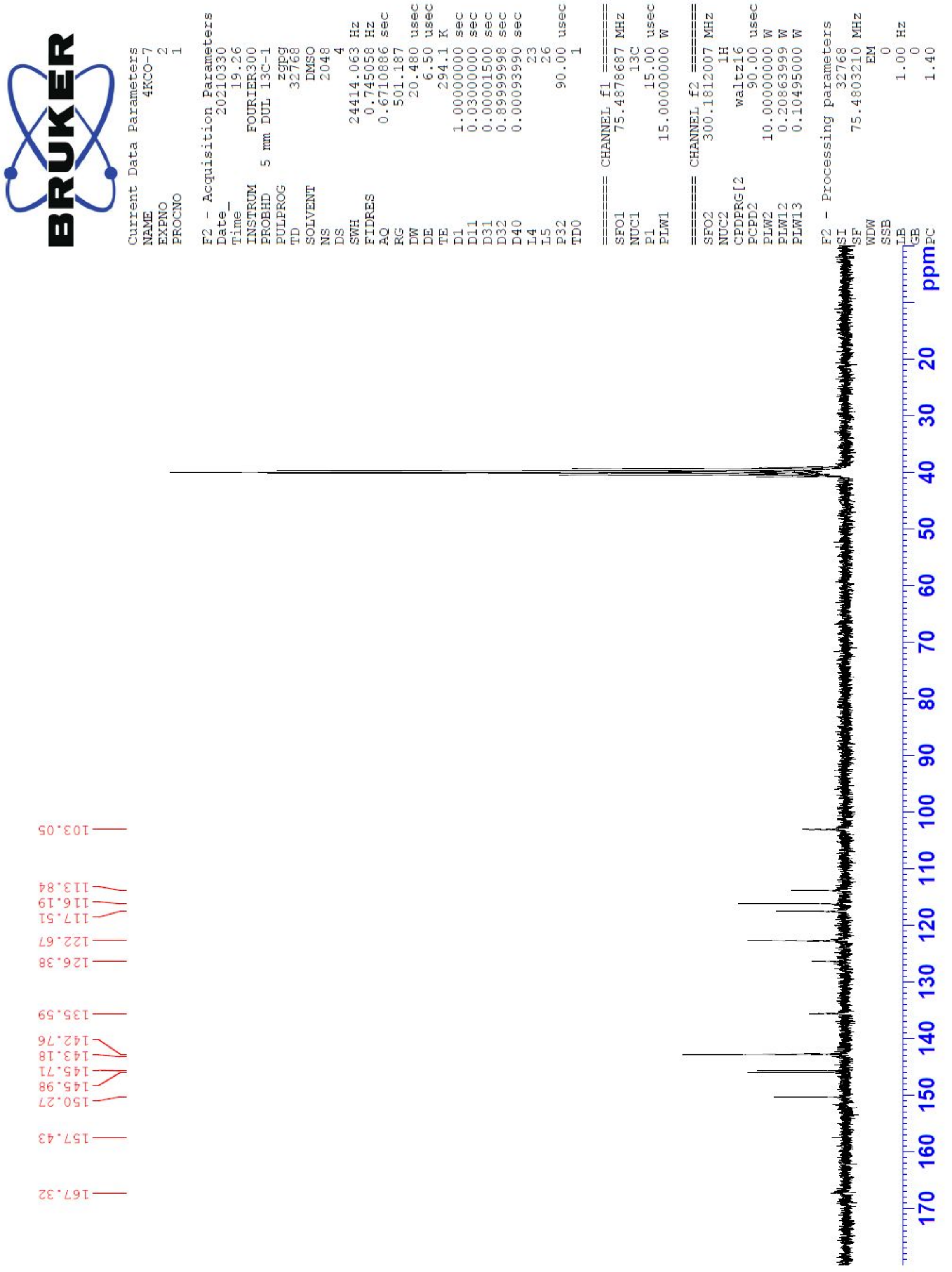

Figure S 29. 13C NMR of **2n**.

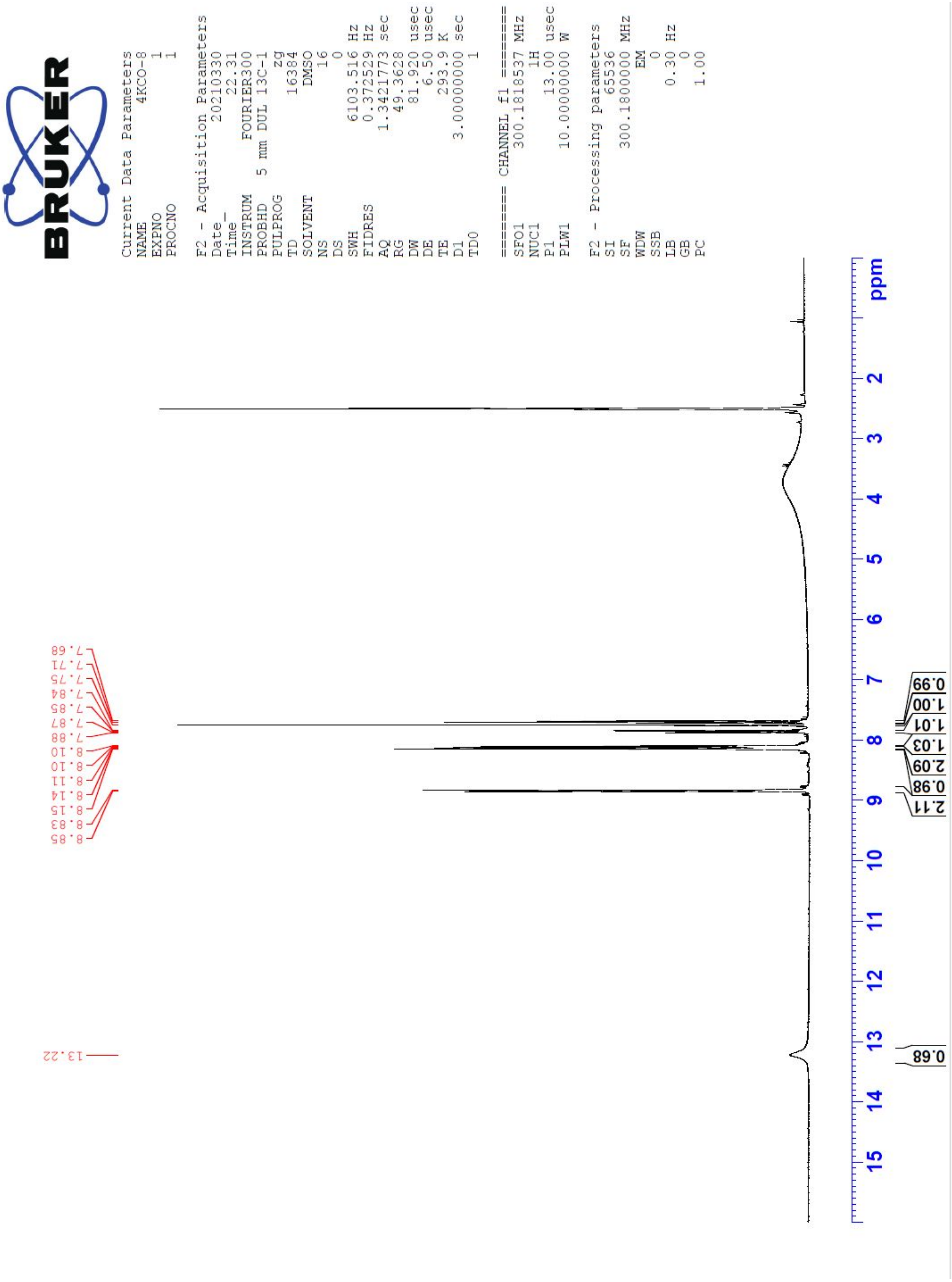

Figure S 30. 1H NMR of **2o**.

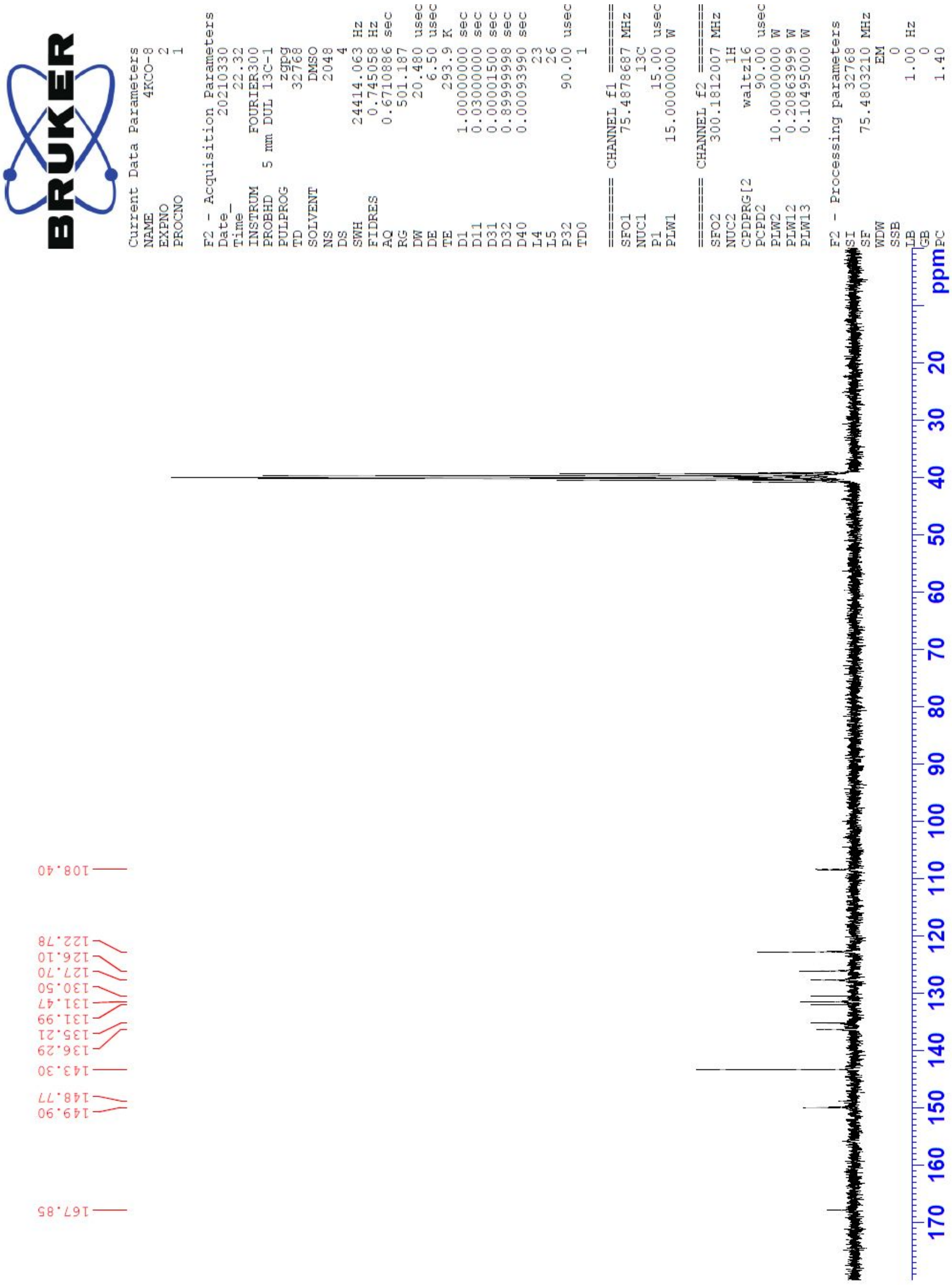

Figure S 31. 13C NMR of **2o**.

Data File: C:\LabSolutions\Data\Analiz\A.Çağrı\2KCO-4_20.lcd

Elmt	Val.	Min	Max	Elmt	Val.	Min	Max	Elmt	Val.	Min	Max	Elmt	Val.	Min	Max	Use Adduct
H	1	0	40	O	2	0	7	S	2	1	3	Ru	2	0	0	H
C	4	0	40	F	1	0	0	Cl	1	0	2	Pd	2	0	0	
N	3	4	7	P	3	0	0	Br	1	0	0	I	3	0	0	

Error Margin (ppm):	5	DBE Range:	10.0 - 25.0	Electron Ions:	both
HC Ratio:	unlimited	Apply N Rule:	yes	Use MSn Info:	yes
Max Isotopes:	3	Isotope RI (%):	1.00	Isotope Res:	9000
MSn Iso RI (%):	10.00	MSn Logic Mode:	AND	Max Results:	150

Event#: 1 MS(E+) Ret. Time : 9.013 -> 9.240 Scan# : 1353 -> 1387

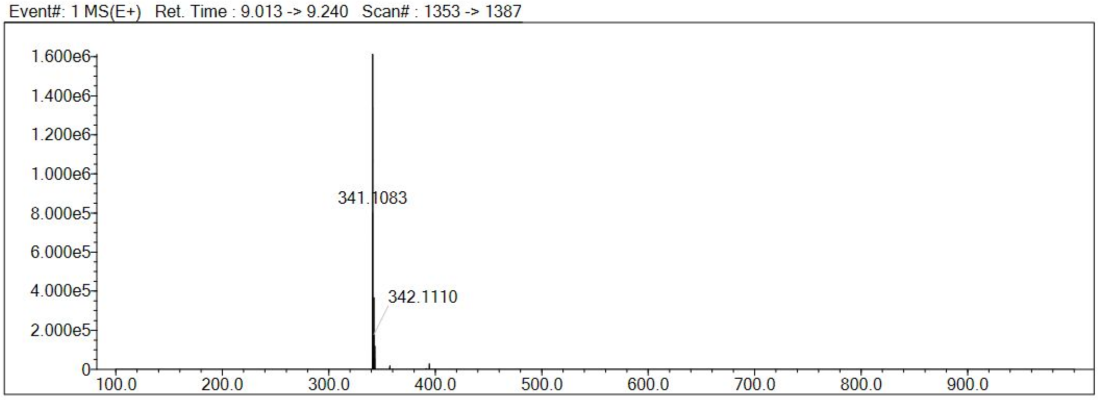

Measured region for 341.1083 m/z

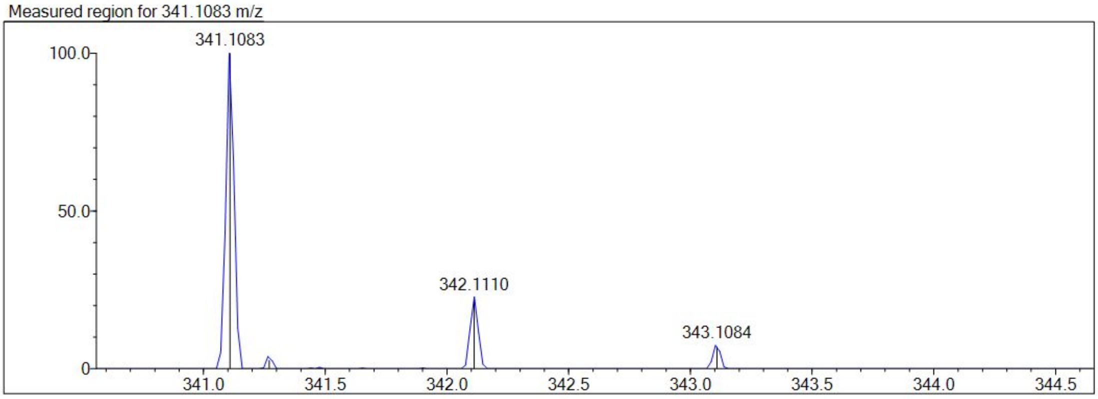

C17 H16 N4 O2 S [M+H]+ : Predicted region for 341.1067 m/z

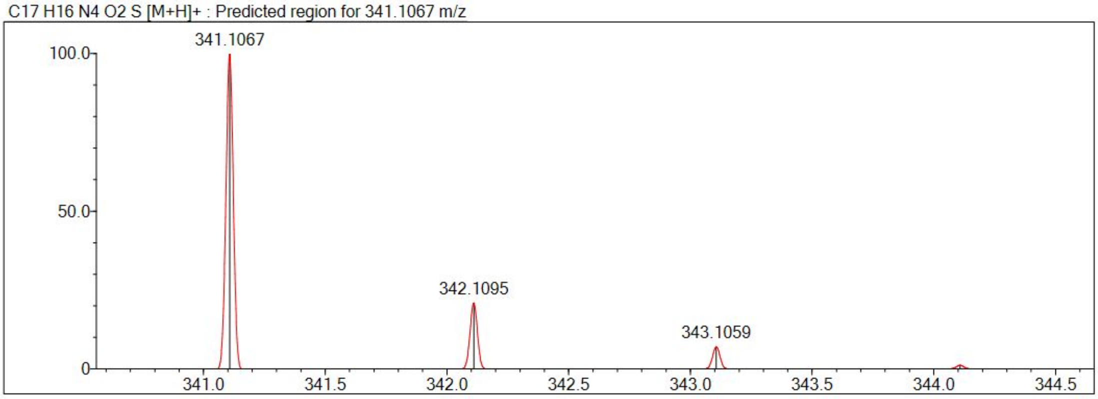

Rank	Score	Formula (M)	Ion	Meas. m/z	Pred. m/z	Df. (mDa)	Df. (ppm)	Iso	DBE
1	87.36	C17 H16 N4 O2 S	[M+H]+	341.1083	341.1067	1.6	4.69	96.24	12.0

Figure S 32. HRMS spectrum of **2a**.

Formula Predictor Report - 2KCO-5_21.lcd Page 1 of 1

Data File: C:\LabSolutions\Data\Analiz\A.Çağrı\2KCO-5_21.lcd

Elmt	Val.	Min	Max	Elmt	Val.	Min	Max	Elmt	Val.	Min	Max	Elmt	Val.	Min	Max	Use Adduct
H	1	0	40	O	2	0	7	S	2	1	3	Ru	2	0	0	H
C	4	0	40	F	1	0	0	Cl	1	0	2	Pd	2	0	0	
N	3	4	7	P	3	0	0	Br	1	0	0	I	3	0	0	

Error Margin (ppm):	5	DBE Range:	10.0 - 25.0	Electron Ions:	both
HC Ratio:	unlimited	Apply N Rule:	yes	Use MSn Info:	yes
Max Isotopes:	3	Isotope RI (%):	1.00	Isotope Res:	9000
MSn Iso RI (%):	10.00	MSn Logic Mode:	AND	Max Results:	150

Event#: 1 MS(E+) Ret. Time : 3.493 -> 3.493 - 3.133 -> 3.131 Scan# : 525 -> 525 - 471 -> 471

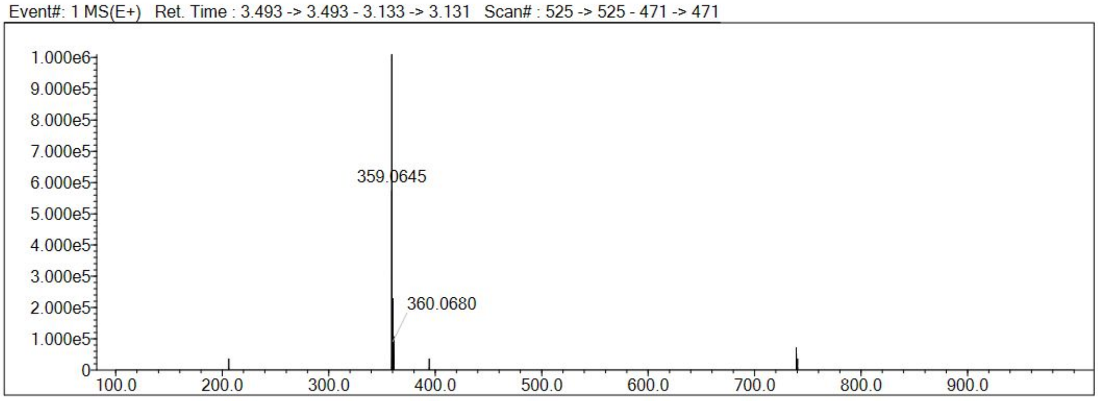

Measured region for 359.0645 m/z

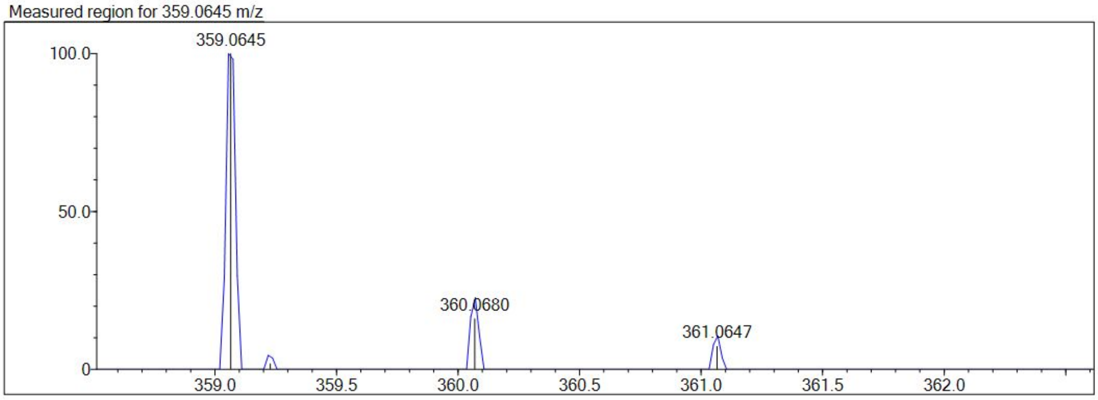

C16 H14 N4 O2 S2 [M+H]+ : Predicted region for 359.0631 m/z

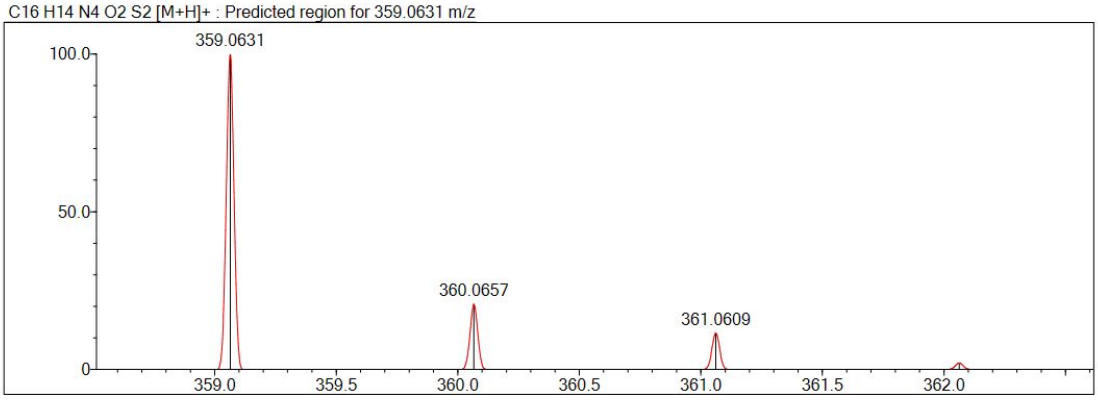

Rank	Score	Formula (M)	Ion	Meas. m/z	Pred. m/z	Df. (mDa)	Df. (ppm)	Iso	DBE
1	81.80	C16 H14 N4 O2 S2	[M+H]+	359.0645	359.0631	1.4	3.90	88.19	12.0

Figure S 33. HRMS spectrum of **2b**.

Data File: C:\LabSolutions\Data\Analiz\A.Çağrı\2KCO-6_22.lcd

Elmt	Val.	Min	Max	Elmt	Val.	Min	Max	Elmt	Val.	Min	Max	Elmt	Val.	Min	Max	Use Adduct
H	1	0	40	O	2	0	7	S	2	1	3	Ru	2	0	0	H
C	4	0	40	F	1	0	0	Cl	1	0	2	Pd	2	0	0	
N	3	4	7	P	3	0	0	Br	1	0	0	I	3	0	0	

Error Margin (ppm):	5	DBE Range:	10.0 - 25.0	Electron Ions:	both
HC Ratio:	unlimited	Apply N Rule:	yes	Use MSn Info:	yes
Max Isotopes:	3	Isotope RI (%):	1.00	Isotope Res:	9000
MSn Iso RI (%):	10.00	MSn Logic Mode:	AND	Max Results:	150

Event#: 1 MS(E+) Ret. Time : 8.547 -> 8.733 Scan# : 1283 -> 1311

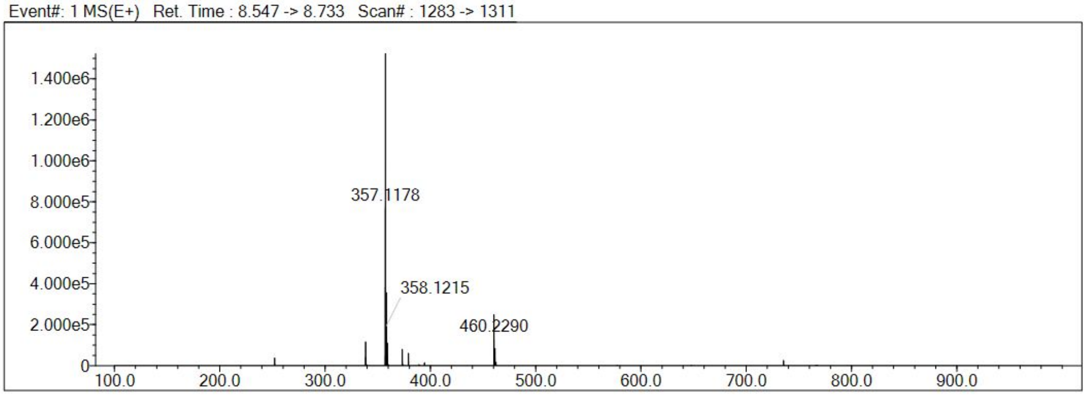

Measured region for 357.1178 m/z

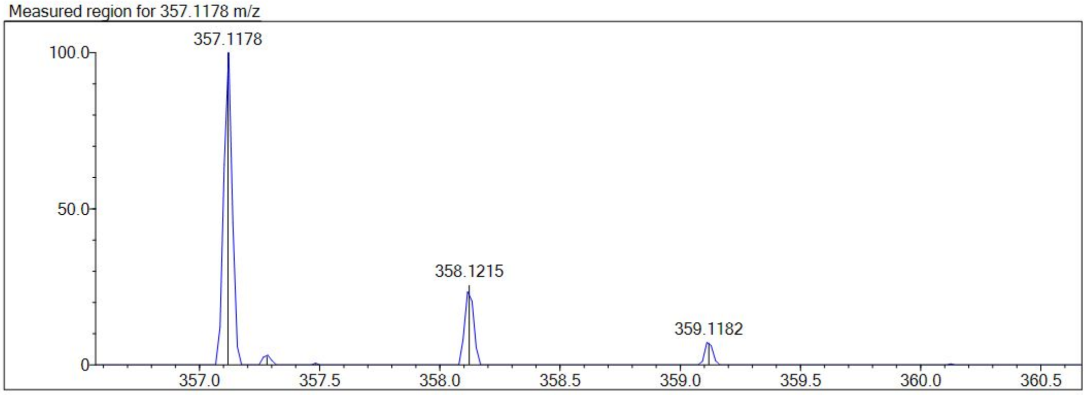

C21 H16 N4 S [M+H]+ : Predicted region for 357.1168 m/z

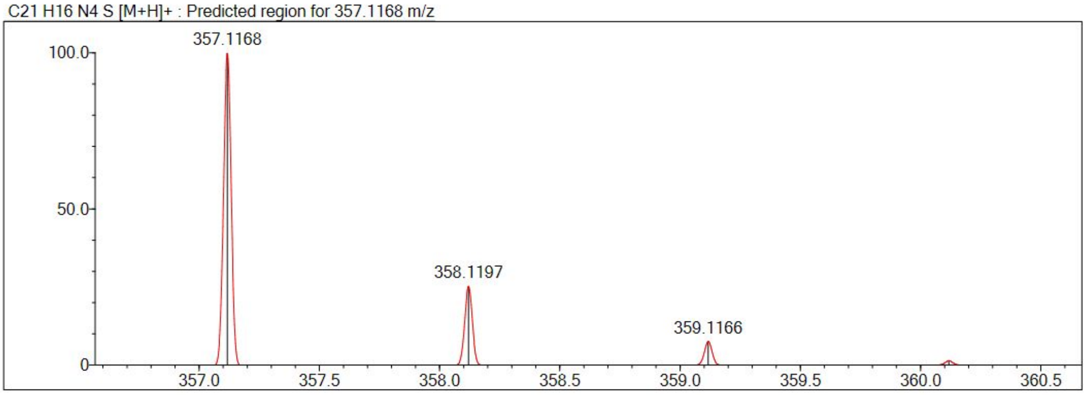

Rank	Score	Formula (M)	Ion	Meas. m/z	Pred. m/z	Df. (mDa)	Df. (ppm)	Iso	DBE
1	66.67	C21 H16 N4 S	[M+H]+	357.1178	357.1168	1.0	2.80	69.81	16.0

Figure S 34. HRMS spectrum of **2c**.

Data File: C:\LabSolutions\Data\Analiz\A.Çağrı\2KCO-7_23.lcd

Elmt	Val.	Min	Max	Elmt	Val.	Min	Max	Elmt	Val.	Min	Max	Elmt	Val.	Min	Max	Use Adduct
H	1	0	40	O	2	0	7	S	2	1	3	Ru	2	0	0	H
C	4	0	40	F	1	0	0	Cl	1	0	2	Pd	2	0	0	
N	3	4	7	P	3	0	0	Br	1	0	0	I	3	0	0	

Error Margin (ppm):	5	DBE Range:	10.0 - 25.0	Electron Ions:	both
HC Ratio:	unlimited	Apply N Rule:	yes	Use MSn Info:	yes
Max Isotopes:	3	Isotope RI (%):	1.00	Isotope Res:	9000
MSn Iso RI (%):	10.00	MSn Logic Mode:	AND	Max Results:	150

Event#: 1 MS(E+) Ret. Time : 3.080 -> 3.160 - 1.933 -> 2.879 Scan# : 463 -> 475 - 291 -> 433

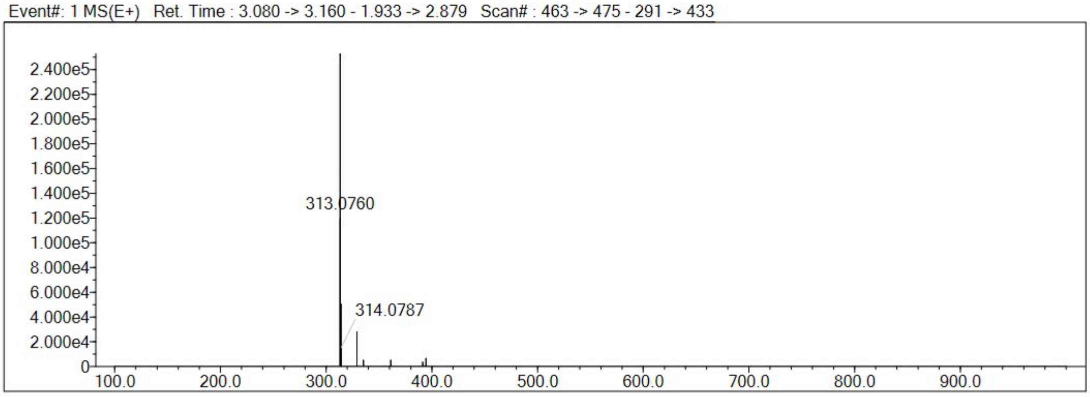

Measured region for 313.0760 m/z

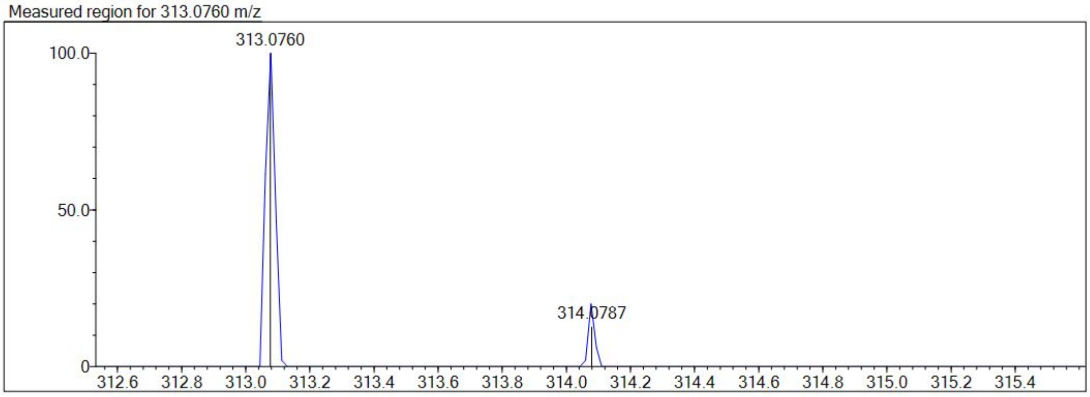

C15 H12 N4 O2 S [M+H]+ : Predicted region for 313.0754 m/z

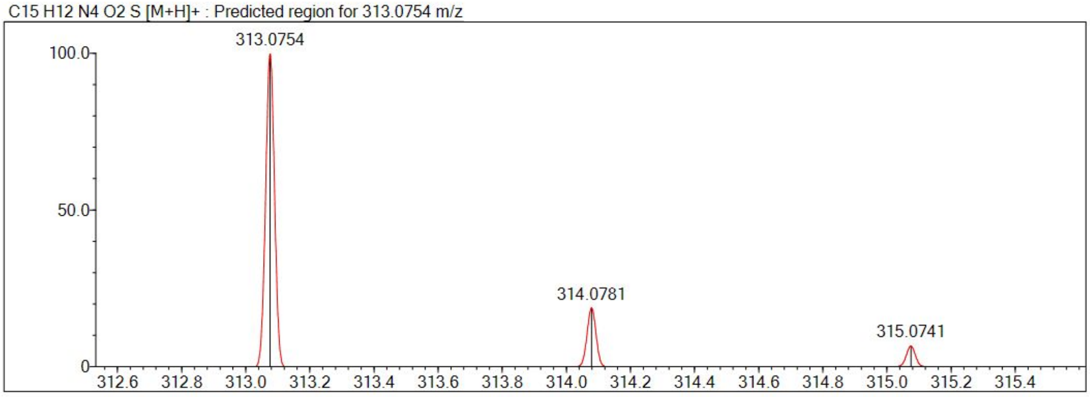

Rank	Score	Formula (M)	Ion	Meas. m/z	Pred. m/z	Df. (mDa)	Df. (ppm)	Iso	DBE
1	74.17	C15 H12 N4 O2 S	[M+H]+	313.0760	313.0754	0.6	1.92	75.91	12.0

Figure S 35. HRMS spectrum of **2d**.

Data File: C:\LabSolutions\Data\Analiz\A.Çağrı\2KCO-8_24.lcd

Elmt	Val.	Min	Max	Elmt	Val.	Min	Max	Elmt	Val.	Min	Max	Elmt	Val.	Min	Max	Use Adduct
H	1	0	40	O	2	0	7	S	2	1	3	Ru	2	0	0	H
C	4	0	40	F	1	0	0	Cl	1	0	2	Pd	2	0	0	
N	3	4	7	P	3	0	0	Br	1	0	0	I	3	0	0	

Error Margin (ppm): 5
HC Ratio: unlimited
Max Isotopes: 3
MSn Iso RI (%): 10.00

DBE Range: 10.0 - 25.0
Apply N Rule: yes
Isotope RI (%): 1.00
MSn Logic Mode: AND

Electron Ions: both
Use MSn Info: yes
Isotope Res: 9000
Max Results: 150

Event#: 1 MS(E+) Ret. Time : 9.027 -> 9.160 - 9.400 -> 9.902 Scan# : 1355 -> 1375 - 1411 -> 1487

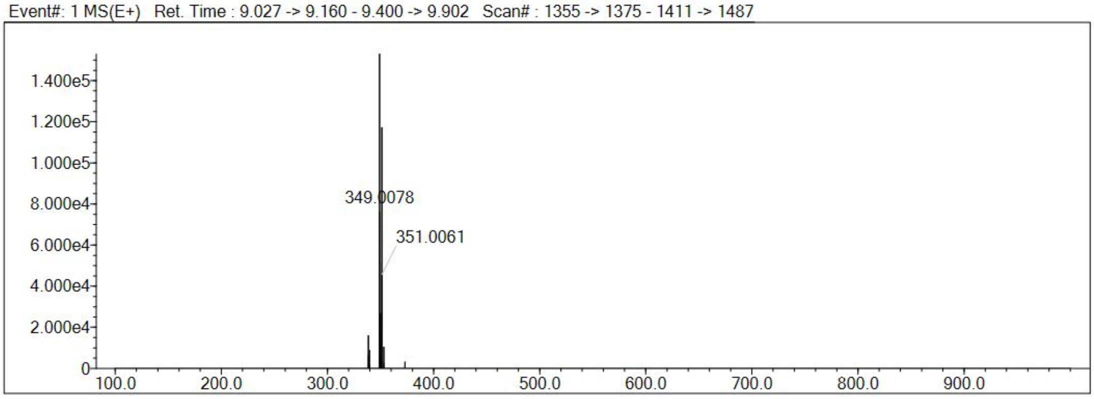

Measured region for 349.0078 m/z

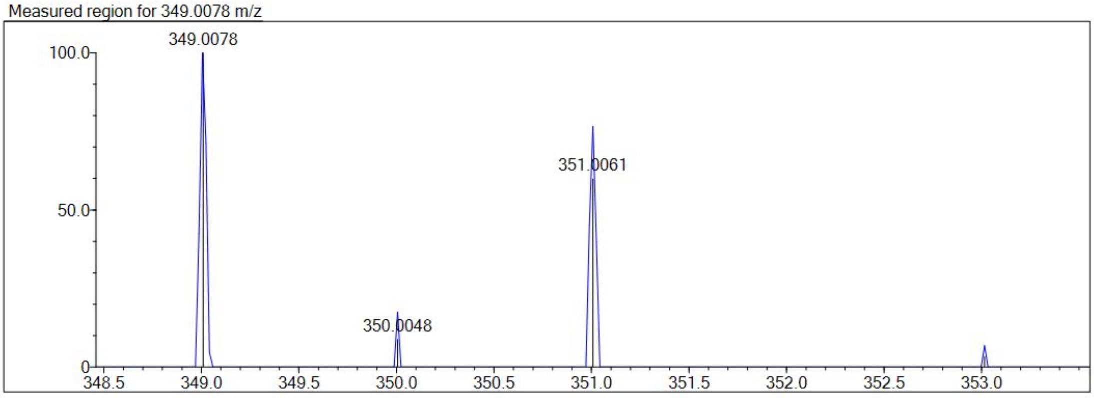

C15 H10 N4 S Cl2 [M+H]+ : Predicted region for 349.0076 m/z

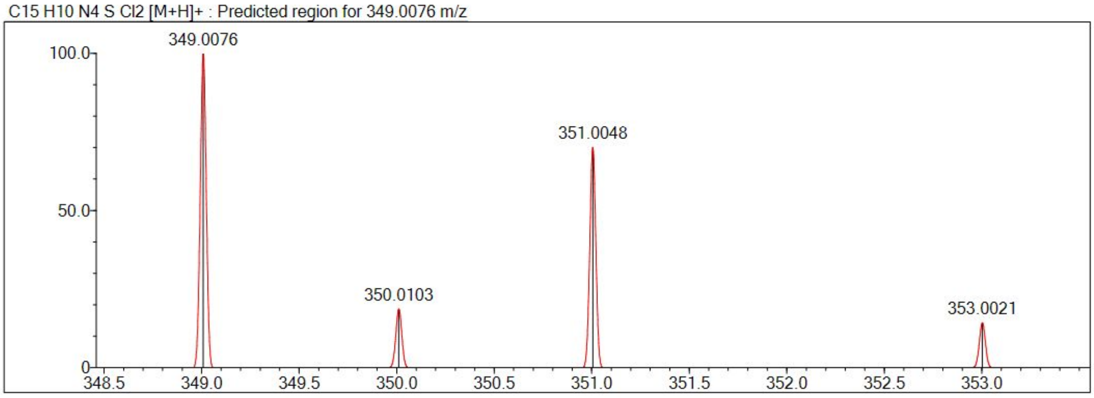

Rank	Score	Formula (M)	Ion	Meas. m/z	Pred. m/z	Df. (mDa)	Df. (ppm)	Iso	DBE
1	30.91	C15 H10 N4 S Cl2	[M+H]+	349.0078	349.0076	0.2	0.57	30.91	12.0

Figure S 36. HRMS spectrum of **2e**.

Data File: C:\LabSolutions\Data\Analiz\A.Çağrı\3KCO-4_25.lcd

Elmt	Val.	Min	Max	Elmt	Val.	Min	Max	Elmt	Val.	Min	Max	Elmt	Val.	Min	Max	Use Adduct
H	1	0	40	O	2	0	7	S	2	1	3	Ru	2	0	0	H
C	4	0	40	F	1	0	0	Cl	1	0	2	Pd	2	0	0	
N	3	4	7	P	3	0	0	Br	1	0	0	I	3	0	0	

Error Margin (ppm):	5	DBE Range:	10.0 - 25.0	Electron Ions:	both
HC Ratio:	unlimited	Apply N Rule:	yes	Use MSn Info:	yes
Max Isotopes:	3	Isotope RI (%):	1.00	Isotope Res:	9000
MSn Iso RI (%):	10.00	MSn Logic Mode:	AND	Max Results:	150

Event#: 1 MS(E+) Ret. Time : 4.240 -> 4.547 Scan# : 637 -> 683

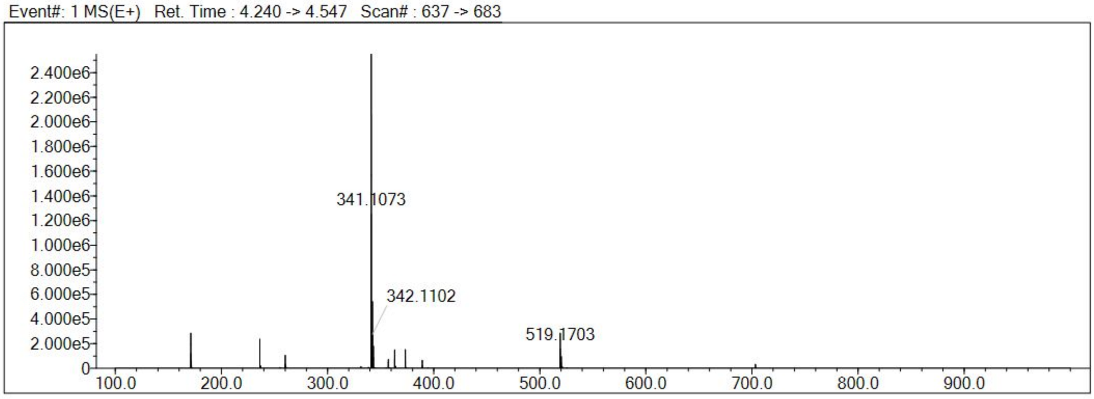

Measured region for 341.1073 m/z

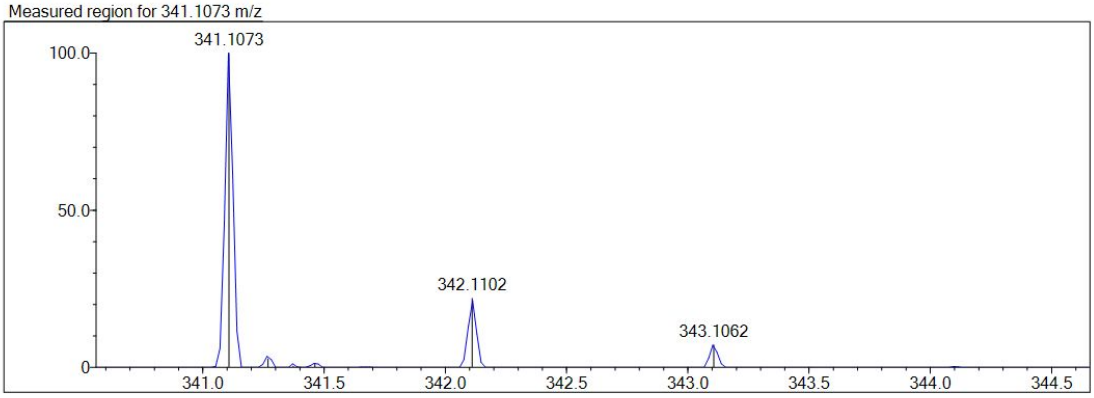

C17 H16 N4 O2 S [M+H]+ : Predicted region for 341.1067 m/z

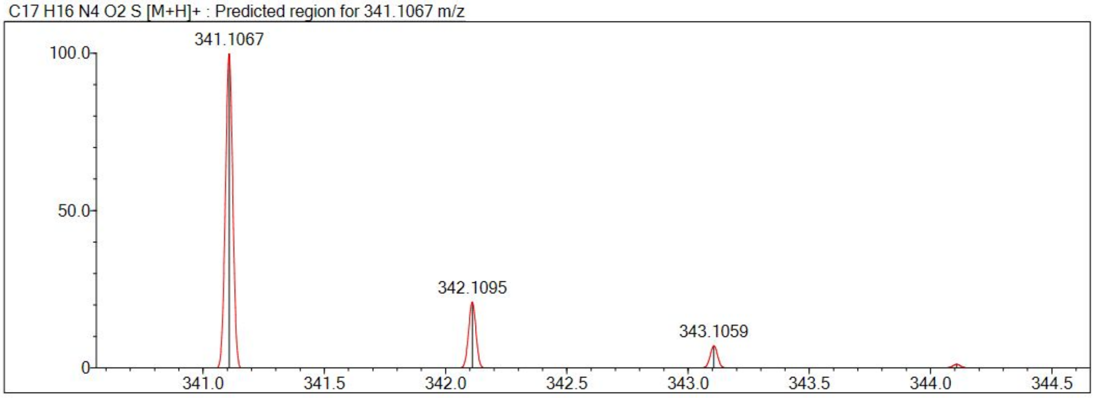

Rank	Score	Formula (M)	Ion	Meas. m/z	Pred. m/z	Df. (mDa)	Df. (ppm)	Iso	DBE
1	81.10	C17 H16 N4 O2 S	[M+H]+	341.1073	341.1067	0.6	1.76	82.67	12.0

Figure S 37. HRMS spectrum of **2f**.

Formula Predictor Report - 3KCO-5_26.lcd Page 1 of 1

Data File: C:\LabSolutions\Data\Analiz\A.Çağrı\3KCO-5_26.lcd

Elmt	Val.	Min	Max	Elmt	Val.	Min	Max	Elmt	Val.	Min	Max	Elmt	Val.	Min	Max	Use Adduct
H	1	0	40	O	2	0	7	S	2	1	3	Ru	2	0	0	H
C	4	0	40	F	1	0	0	Cl	1	0	2	Pd	2	0	0	
N	3	4	7	P	3	0	0	Br	1	0	0	I	3	0	0	

Error Margin (ppm):	5	DBE Range:	10.0 - 25.0	Electron Ions:	both
HC Ratio:	unlimited	Apply N Rule:	yes	Use MSn Info:	yes
Max Isotopes:	3	Isotope RI (%):	1.00	Isotope Res:	9000
MSn Iso RI (%):	10.00	MSn Logic Mode:	AND	Max Results:	150

Event#: 1 MS(E+) Ret. Time : 3.187 -> 3.453 Scan# : 479 -> 519

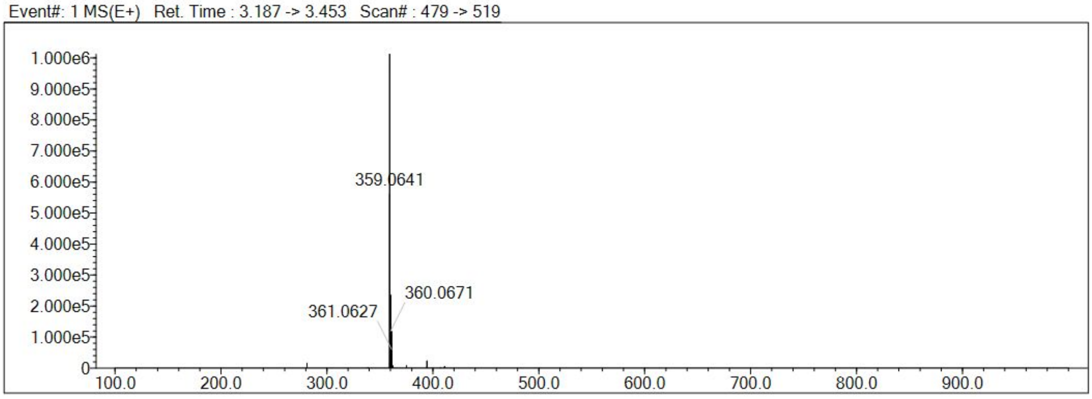

Measured region for 359.0641 m/z

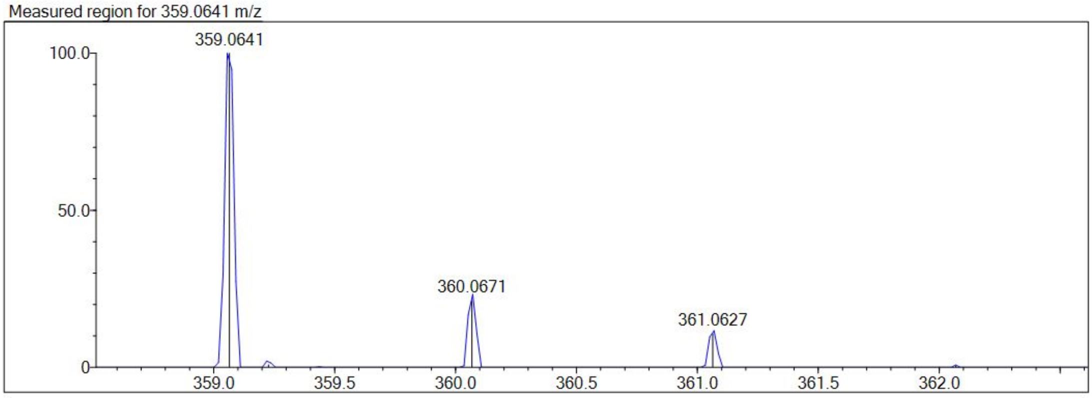

C16 H14 N4 O2 S2 [M+H]+ : Predicted region for 359.0631 m/z

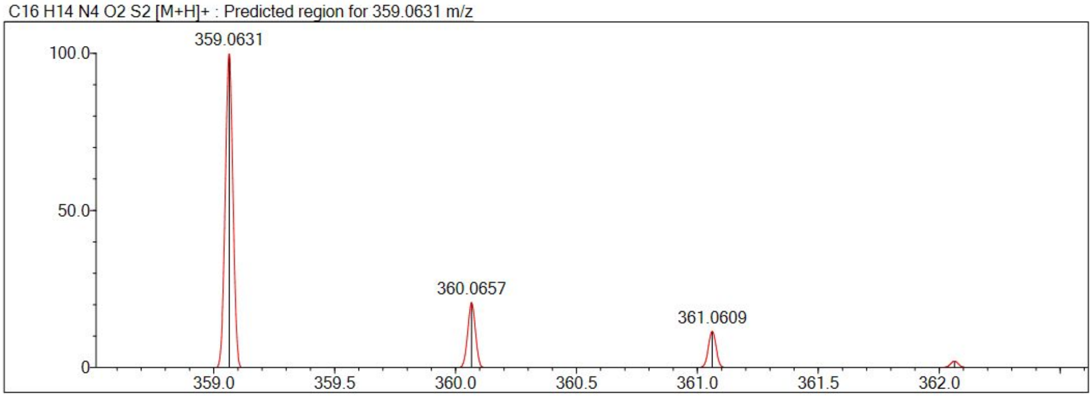

Rank	Score	Formula (M)	Ion	Meas. m/z	Pred. m/z	Df. (mDa)	Df. (ppm)	Iso	DBE
1	83.68	C16 H14 N4 O2 S2	[M+H]+	359.0641	359.0631	1.0	2.79	87.60	12.0

Figure S 38. HRMS spectrum of **2g**.

Data File: C:\LabSolutions\Data\Analiz\A.Çağrı\3KCO-6_27.lcd

Elmt	Val.	Min	Max	Elmt	Val.	Min	Max	Elmt	Val.	Min	Max	Elmt	Val.	Min	Max	Use Adduct
H	1	0	40	O	2	0	7	S	2	1	3	Ru	2	0	0	H
C	4	0	40	F	1	0	0	Cl	1	0	2	Pd	2	0	0	
N	3	4	7	P	3	0	0	Br	1	0	0	I	3	0	0	

Error Margin (ppm):	5	DBE Range:	10.0 - 25.0	Electron Ions:	both
HC Ratio:	unlimited	Apply N Rule:	yes	Use MSn Info:	yes
Max Isotopes:	3	Isotope RI (%):	1.00	Isotope Res:	9000
MSn Iso RI (%):	10.00	MSn Logic Mode:	AND	Max Results:	150

Event#: 1 MS(E+) Ret. Time : 7.373 -> 7.373 - 7.707 -> 9.625 Scan# : 1107 -> 1107 - 1157 -> 1445

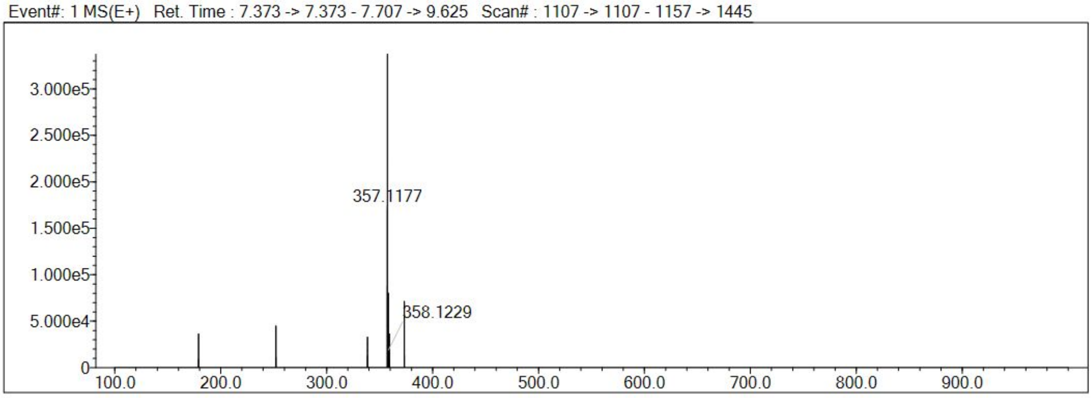

Measured region for 357.1177 m/z

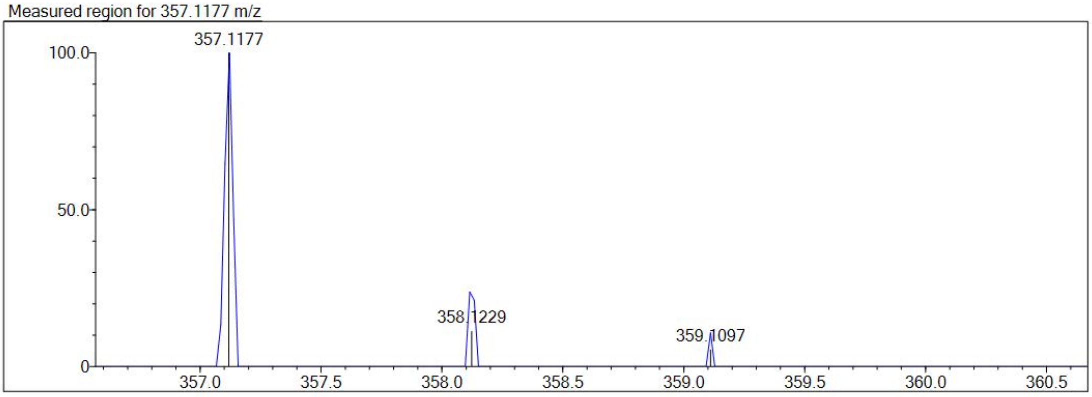

C21 H16 N4 S [M+H]+ : Predicted region for 357.1168 m/z

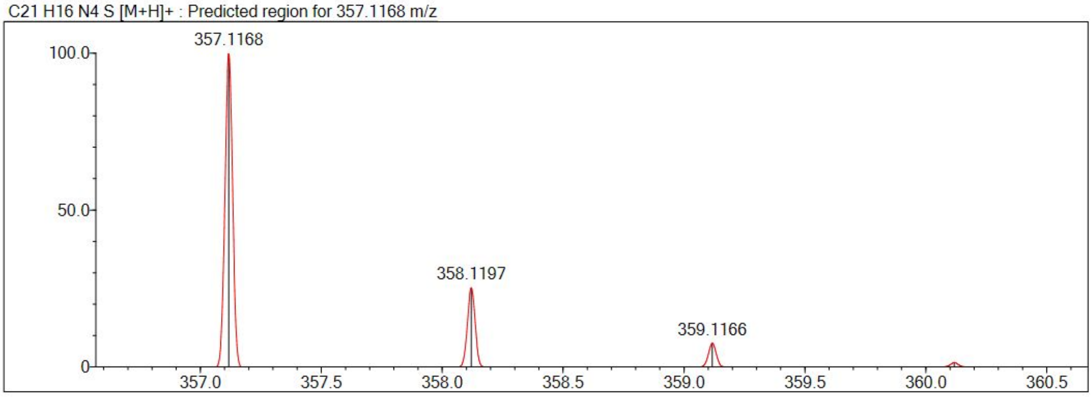

Rank	Score	Formula (M)	Ion	Meas. m/z	Pred. m/z	Df. (mDa)	Df. (ppm)	Iso	DBE
1	56.78	C21 H16 N4 S	[M+H]+	357.1177	357.1168	0.9	2.52	59.02	16.0

Figure S 39. HRMS spectrum of **2h**.

Data File: C:\LabSolutions\Data\Analiz\A.Çağrı\3KCO-8_29.lcd

Elmt	Val.	Min	Max	Elmt	Val.	Min	Max	Elmt	Val.	Min	Max	Elmt	Val.	Min	Max	Use Adduct
H	1	0	40	O	2	0	7	S	2	1	3	Ru	2	0	0	H
C	4	0	40	F	1	0	0	Cl	1	0	2	Pd	2	0	0	
N	3	4	7	P	3	0	0	Br	1	0	0	I	3	0	0	

Error Margin (ppm): 5
HC Ratio: unlimited
Max Isotopes: 3
MSn Iso RI (%): 10.00

DBE Range: 10.0 - 25.0
Apply N Rule: yes
Isotope RI (%): 1.00
MSn Logic Mode: AND

Electron Ions: both
Use MSn Info: yes
Isotope Res: 9000
Max Results: 150

Event#: 1 MS(E+) Ret. Time : 7.387 -> 7.520 - 5.560 -> 7.227 Scan# : 1109 -> 1129 - 835 -> 1085

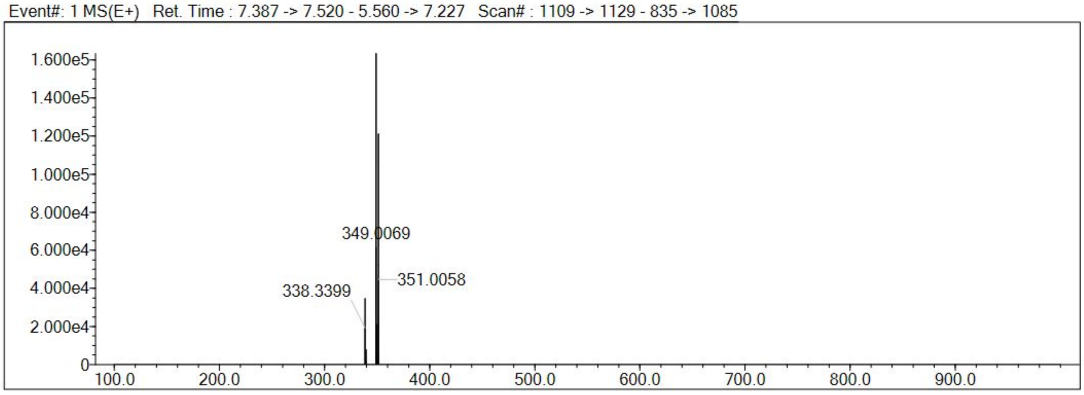

Measured region for 349.0069 m/z

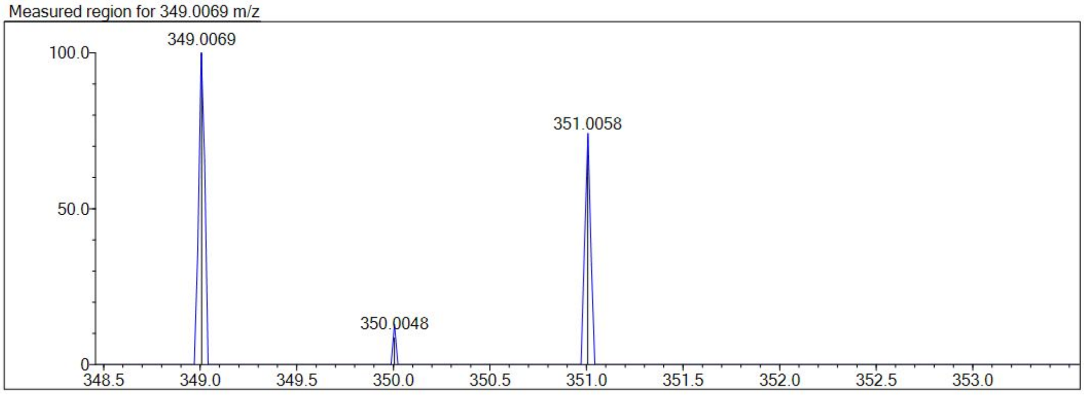

C15 H10 N4 S Cl2 [M+H]+ : Predicted region for 349.0076 m/z

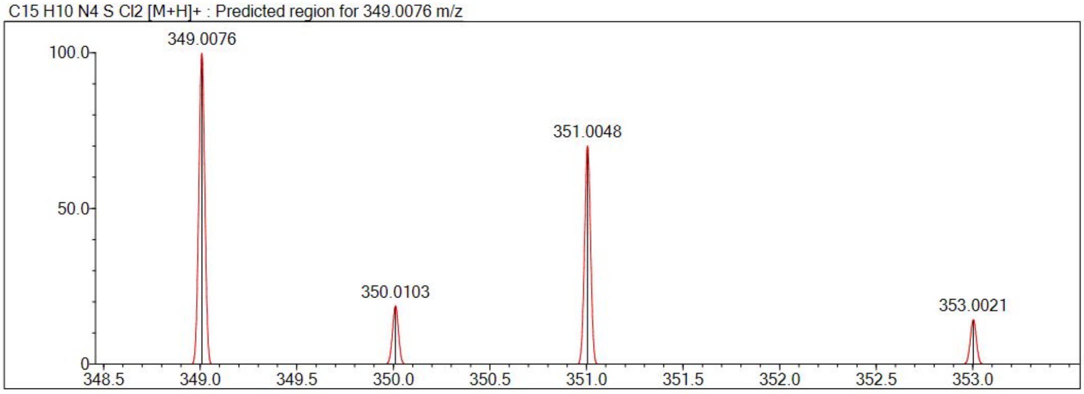

Rank	Score	Formula (M)	Ion	Meas. m/z	Pred. m/z	Df. (mDa)	Df. (ppm)	Iso	DBE
1	53.17	C15 H10 N4 S Cl2	[M+H]+	349.0069	349.0076	-0.7	-2.01	54.54	12.0

Figure S 40. HRMS spectrum of **2j**.

Data File: C:\LabSolutions\Data\Analiz\A.Çağrı\4KCO-4_30.lcd

Elmt	Val.	Min	Max	Elmt	Val.	Min	Max	Elmt	Val.	Min	Max	Elmt	Val.	Min	Max	Use Adduct
H	1	0	40	O	2	0	7	S	2	1	3	Ru	2	0	0	H
C	4	0	40	F	1	0	0	Cl	1	0	2	Pd	2	0	0	
N	3	4	7	P	3	0	0	Br	1	0	0	I	3	0	0	

Error Margin (ppm): 5
HC Ratio: unlimited
Max Isotopes: 3
MSn Iso RI (%): 10.00

DBE Range: 10.0 - 25.0
Apply N Rule: yes
Isotope RI (%): 1.00
MSn Logic Mode: AND

Electron Ions: both
Use MSn Info: yes
Isotope Res: 9000
Max Results: 150

Event#: 1 MS(E+) Ret. Time : 3.640 -> 3.733 Scan# : 547 -> 561

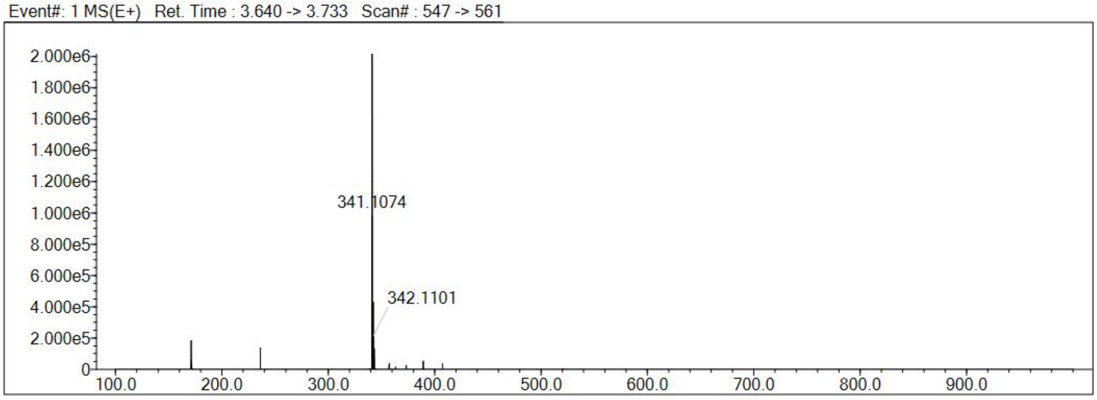

Measured region for 341.1074 m/z

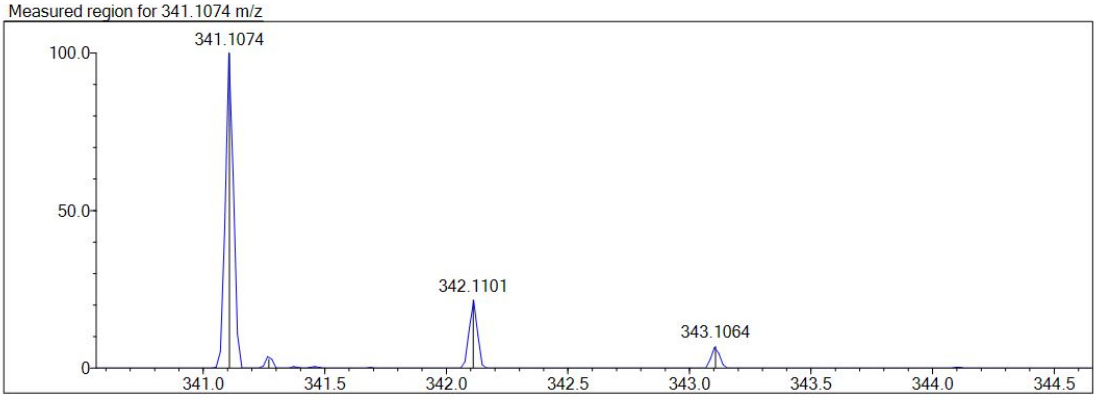

C17 H16 N4 O2 S [M+H]+ : Predicted region for 341.1067 m/z

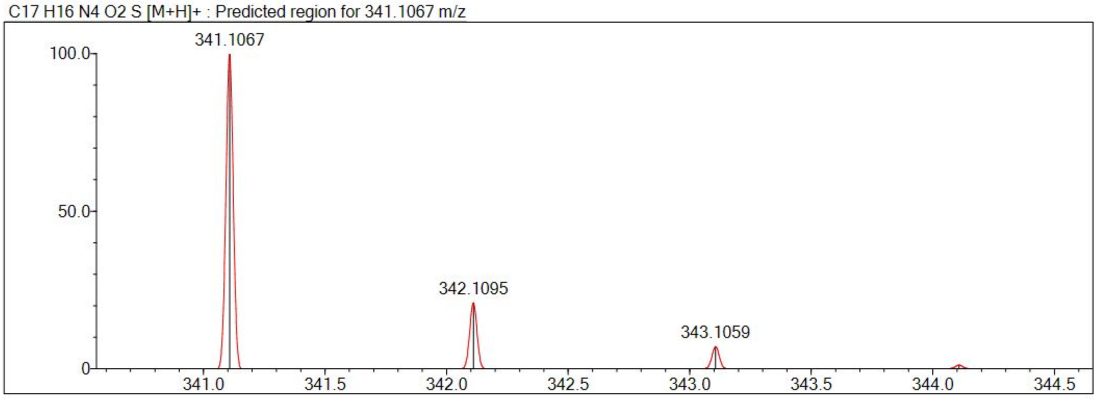

Rank	Score	Formula (M)	Ion	Meas. m/z	Pred. m/z	Df. (mDa)	Df. (ppm)	Iso	DBE
1	97.18	C17 H16 N4 O2 S	[M+H]+	341.1074	341.1067	0.7	2.05	99.80	12.0

Figure S 41. HRMS spectrum of **2k**.

Data File: C:\LabSolutions\Data\Analiz\A.Çağrı\4KCO-5_31.lcd

Elmt	Val.	Min	Max	Elmt	Val.	Min	Max	Elmt	Val.	Min	Max	Elmt	Val.	Min	Max	Use Adduct
H	1	0	40	O	2	0	7	S	2	1	3	Ru	2	0	0	H
C	4	0	40	F	1	0	0	Cl	1	0	2	Pd	2	0	0	
N	3	4	7	P	3	0	0	Br	1	0	0	I	3	0	0	

Error Margin (ppm):	5	DBE Range:	10.0 - 25.0	Electron Ions:	both
HC Ratio:	unlimited	Apply N Rule:	yes	Use MSn Info:	yes
Max Isotopes:	3	Isotope RI (%):	1.00	Isotope Res:	9000
MSn Iso RI (%):	10.00	MSn Logic Mode:	AND	Max Results:	150

Event#: 1 MS(E+) Ret. Time : 2.947 -> 3.160 Scan# : 443 -> 475

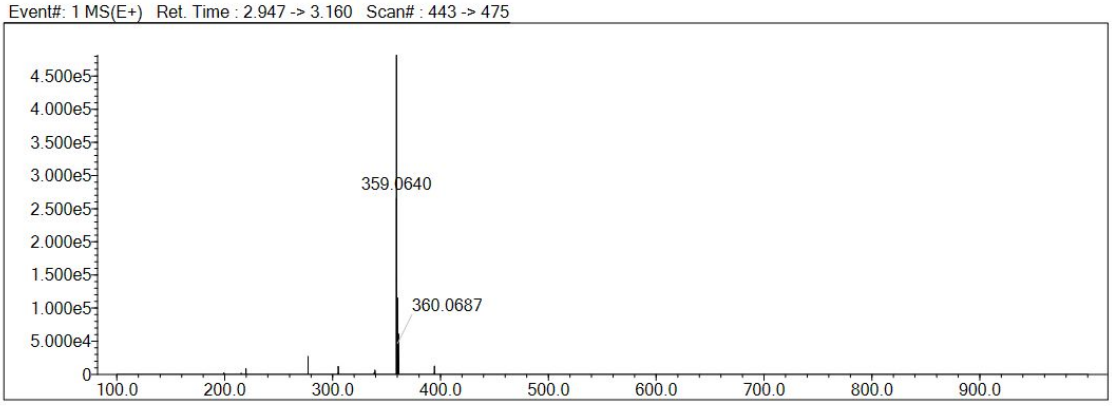

Measured region for 359.0640 m/z

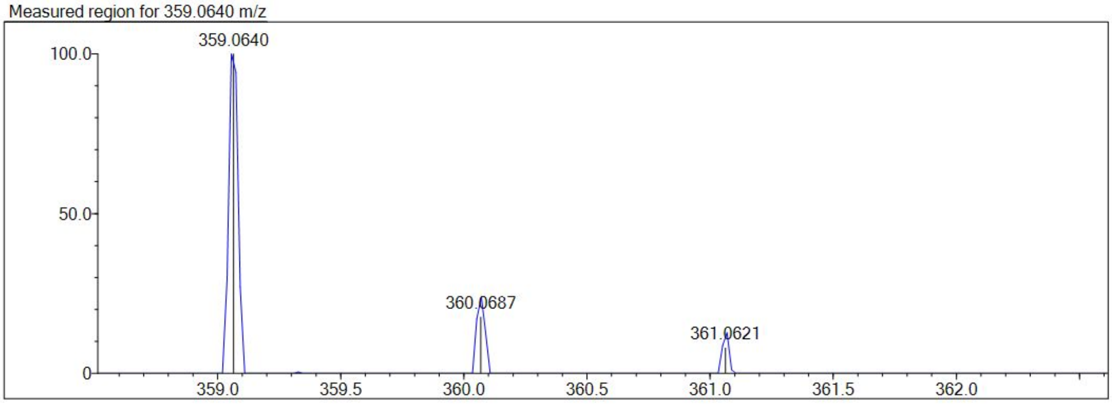

C16 H14 N4 O2 S2 [M+H]+ : Predicted region for 359.0631 m/z

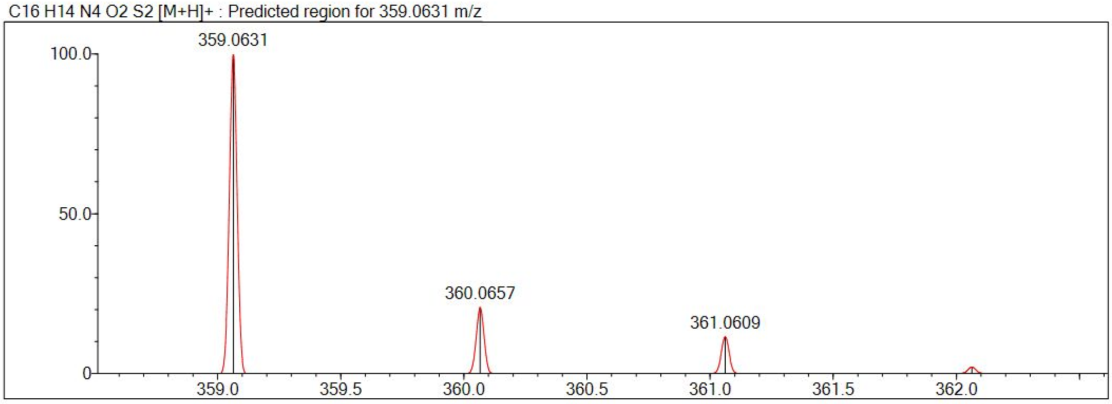

Rank	Score	Formula (M)	Ion	Meas. m/z	Pred. m/z	Df. (mDa)	Df. (ppm)	Iso	DBE
1	86.99	C16 H14 N4 O2 S2	[M+H]+	359.0640	359.0631	0.9	2.51	90.40	12.0

Figure S 42. HRMS spectrum of **2l**.

Data File: C:\LabSolutions\Data\Analiz\A.Çağrı\4KCO-6_32.lcd

Elmt	Val.	Min	Max	Elmt	Val.	Min	Max	Elmt	Val.	Min	Max	Elmt	Val.	Min	Max	Use Adduct
H	1	0	40	O	2	0	7	S	2	1	3	Ru	2	0	0	H
C	4	0	40	F	1	0	0	Cl	1	0	2	Pd	2	0	0	
N	3	4	7	P	3	0	0	Br	1	0	0	I	3	0	0	

Error Margin (ppm): 5
HC Ratio: unlimited
Max Isotopes: 3
MSn Iso RI (%): 10.00

DBE Range: 10.0 - 25.0
Apply N Rule: yes
Isotope RI (%): 1.00
MSn Logic Mode: AND

Electron Ions: both
Use MSn Info: yes
Isotope Res: 9000
Max Results: 150

Event#: 1 MS(E+) Ret. Time : 5.187 -> 5.547 Scan# : 779 -> 833

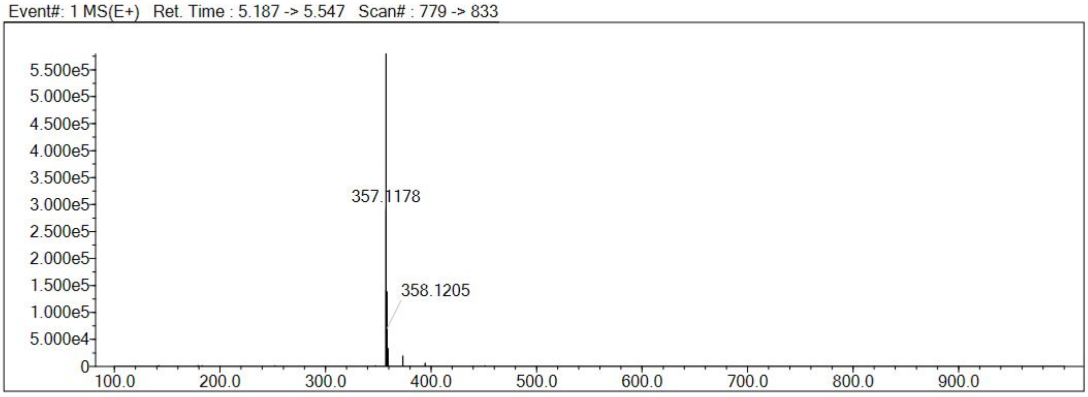

Measured region for 357.1178 m/z

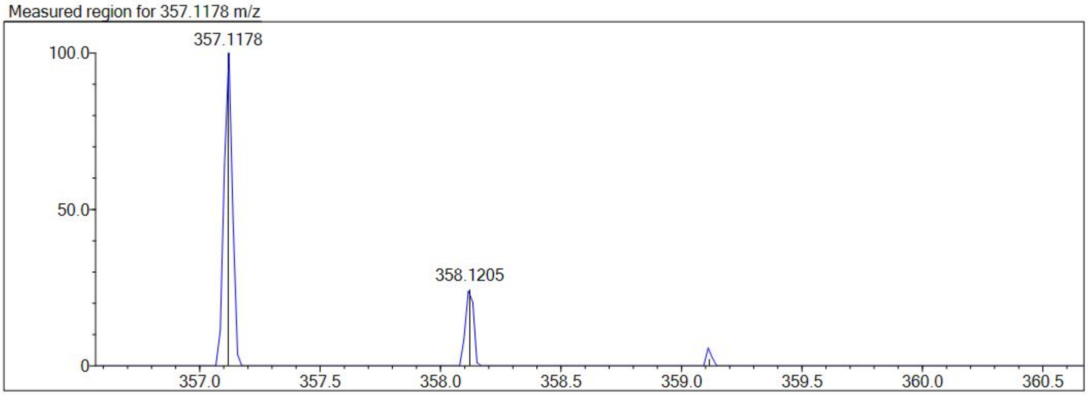

C21 H16 N4 S [M+H]+ : Predicted region for 357.1168 m/z

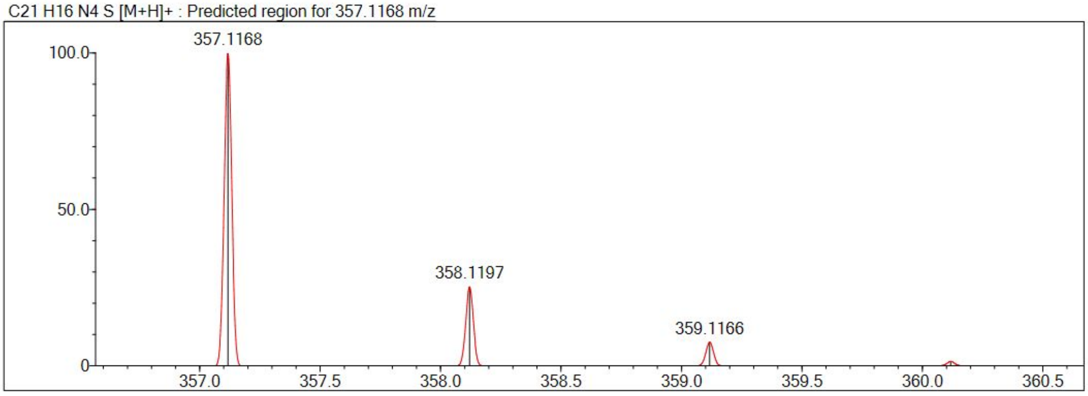

Rank	Score	Formula (M)	Ion	Meas. m/z	Pred. m/z	Df. (mDa)	Df. (ppm)	Iso	DBE
1	64.25	C21 H16 N4 S	[M+H]+	357.1178	357.1168	1.0	2.80	67.28	16.0

Figure S 43. HRMS spectrum of **2m**.

Data File: C:\LabSolutions\Data\Analiz\A.Çağrı\4KCO-8_34.lcd

Elmt	Val.	Min	Max	Elmt	Val.	Min	Max	Elmt	Val.	Min	Max	Elmt	Val.	Min	Max	Use Adduct
H	1	0	40	O	2	0	7	S	2	1	3	Ru	2	0	0	H
C	4	0	40	F	1	0	0	Cl	1	0	2	Pd	2	0	0	
N	3	4	7	P	3	0	0	Br	1	0	0	I	3	0	0	

Error Margin (ppm):	5	DBE Range:	10.0 - 25.0	Electron Ions:	both
HC Ratio:	unlimited	Apply N Rule:	yes	Use MSn Info:	yes
Max Isotopes:	3	Isotope RI (%):	1.00	Isotope Res:	9000
MSn Iso RI (%):	10.00	MSn Logic Mode:	AND	Max Results:	150

Event#: 1 MS(E+) Ret. Time : 5.240 -> 5.387 Scan# : 787 -> 809

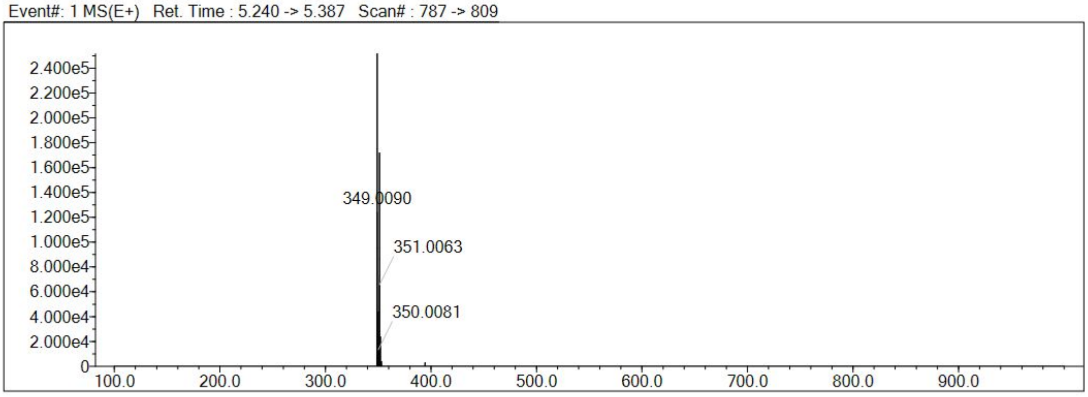

Measured region for 349.0090 m/z

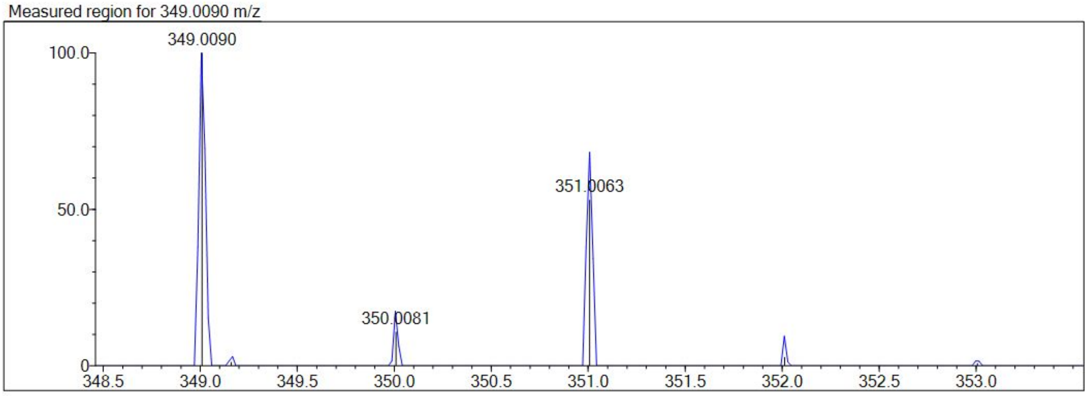

C15 H10 N4 S Cl2 [M+H]+ : Predicted region for 349.0076 m/z

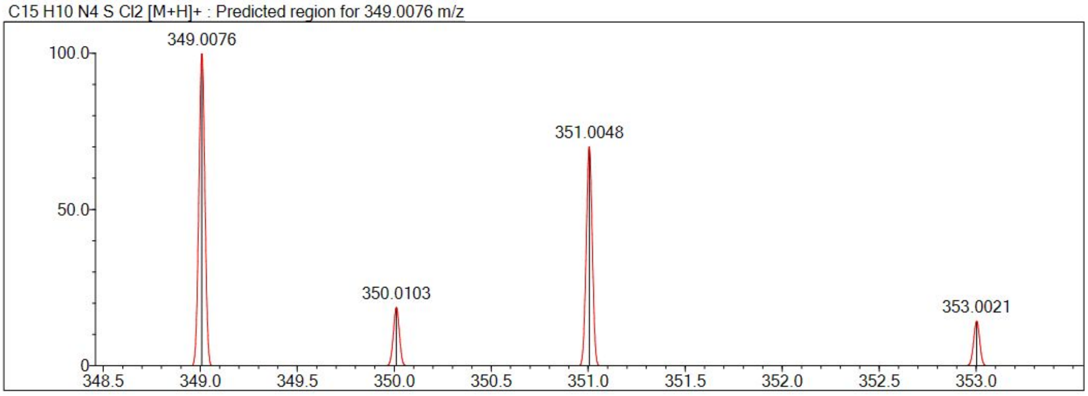

Rank	Score	Formula (M)	Ion	Meas. m/z	Pred. m/z	Df. (mDa)	Df. (ppm)	Iso	DBE
2	46.24	C15 H10 N4 S Cl2	[M+H]+	349.0090	349.0076	1.4	4.01	50.01	12.0

Figure S 44. HRMS spectrum of **2o**.

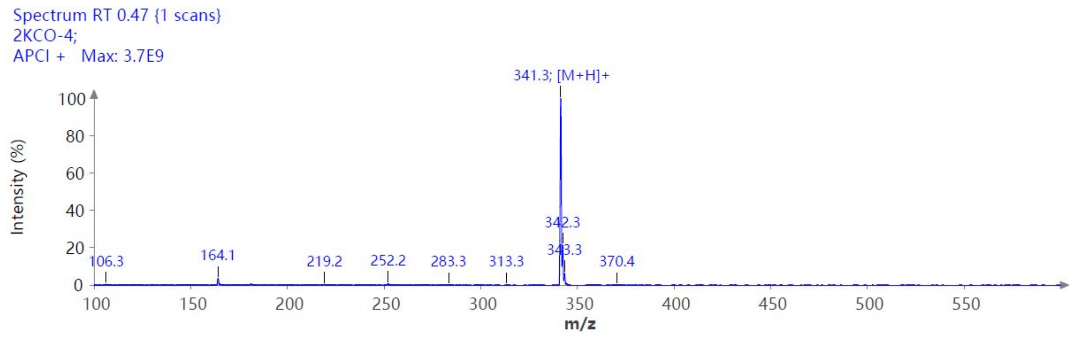

Figure S 45. APCI spectrum of **2a**.

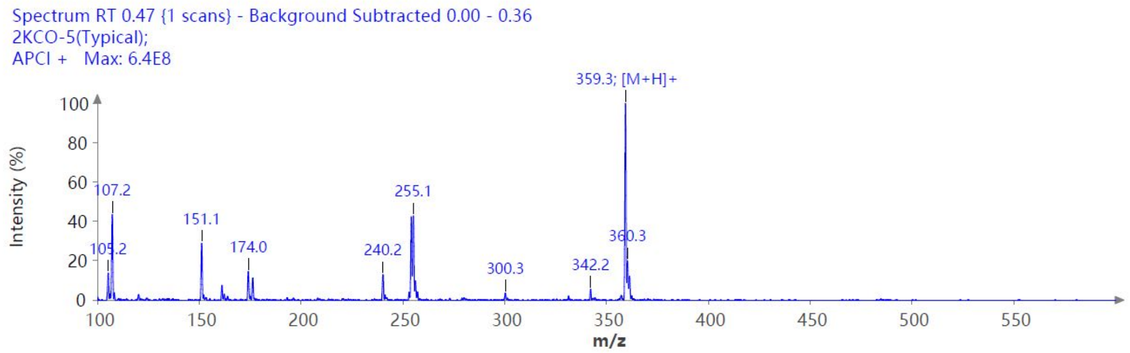

Figure S 46. APCI spectrum of **2b**.

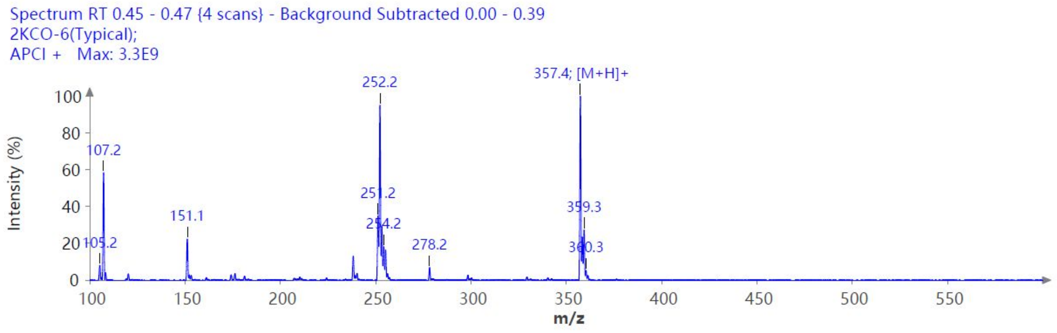

Figure S 47. APCI spectrum of **2c**.

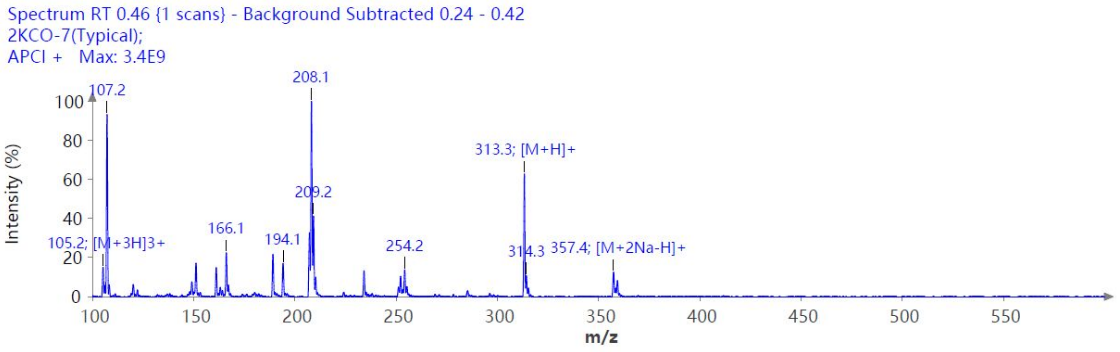

Figure S 48. APCI spectrum of **2d**.

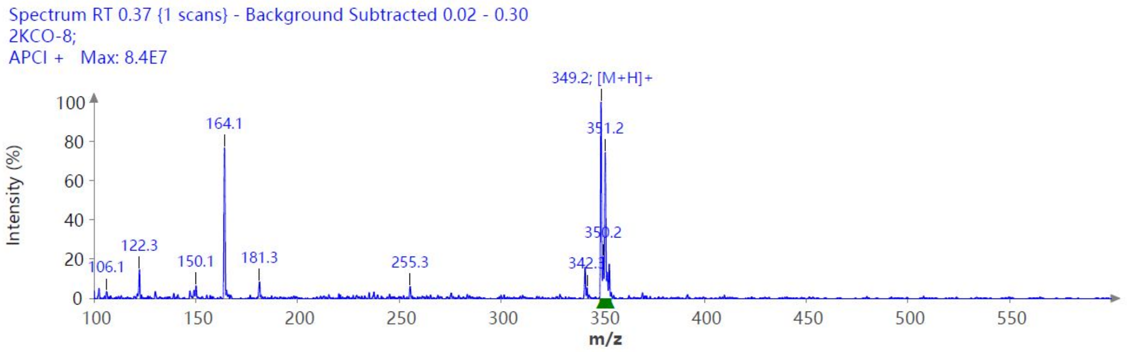

Figure S 49. APCI spectrum of **2e**.

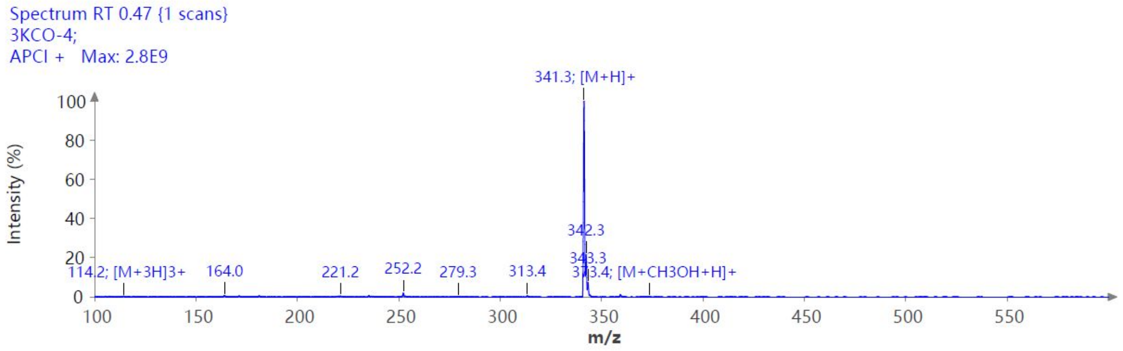

Figure S 50. APCI spectrum of **2f**.

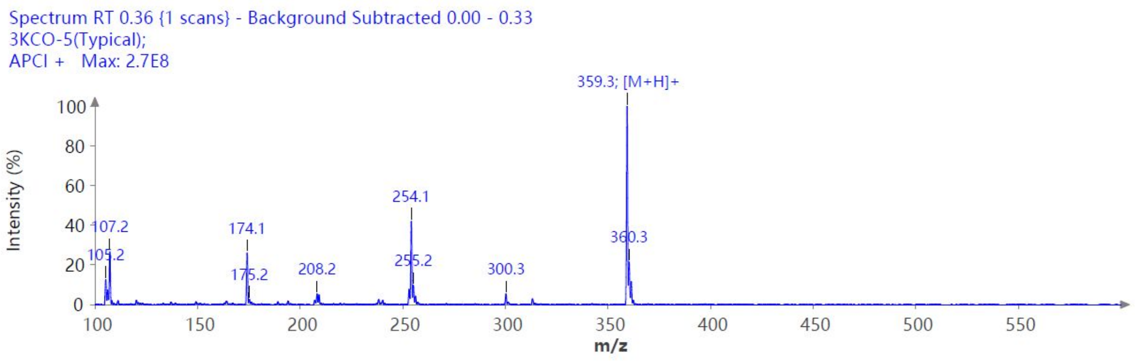

Figure S 51. APCI spectrum of **2g**.

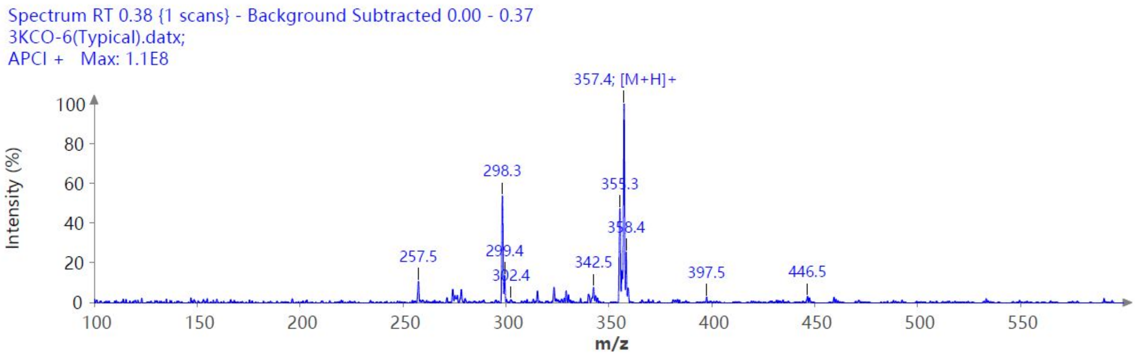

Figure S 52. APCI spectrum of **2h**.

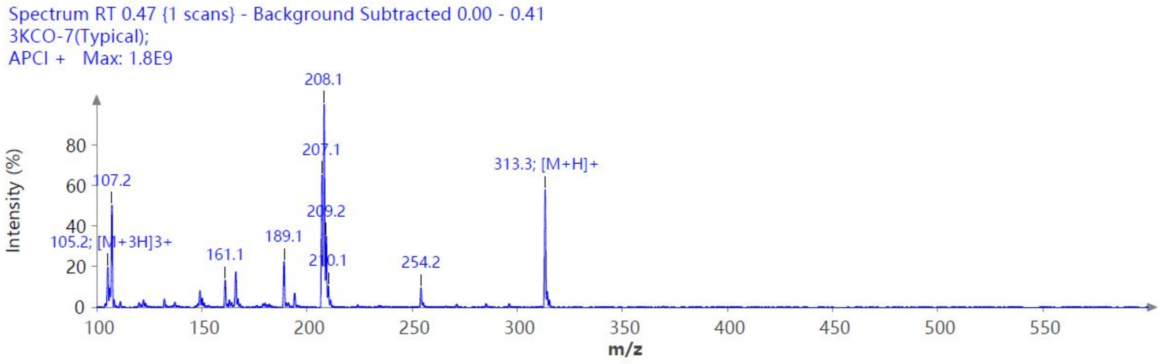

Figure S 53. APCI spectrum of **2i**.

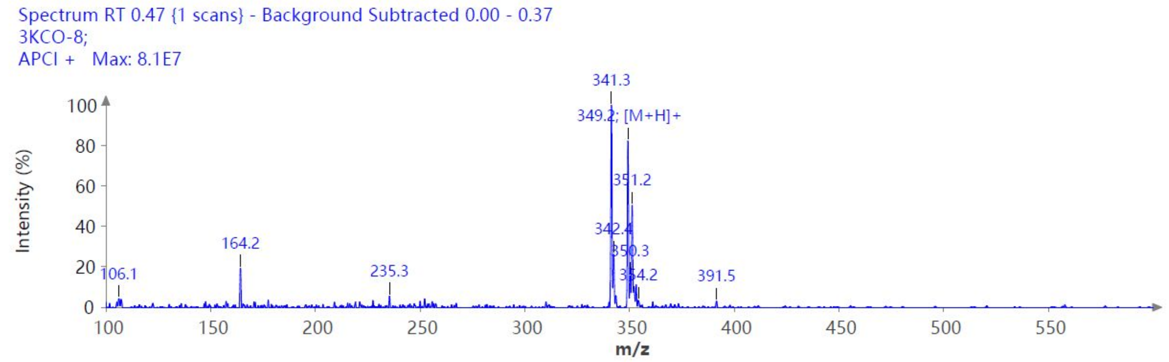

Figure S 54. APCI spectrum of **2j**.

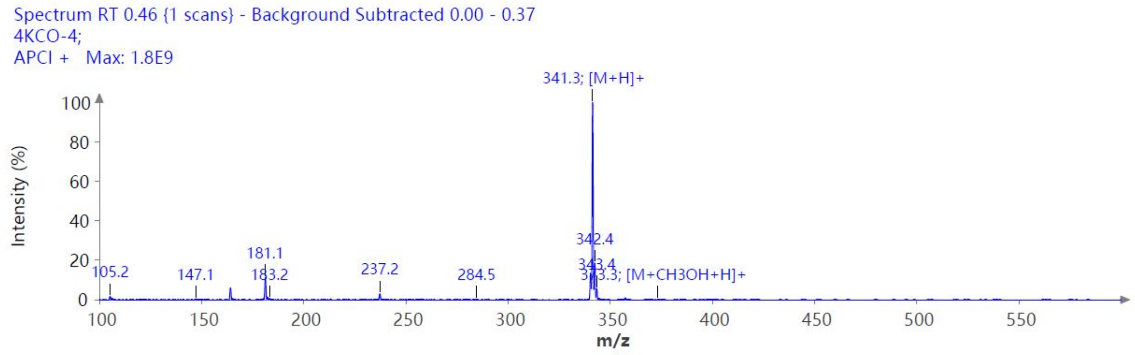

Figure S 55. APCI spectrum of **2k**.

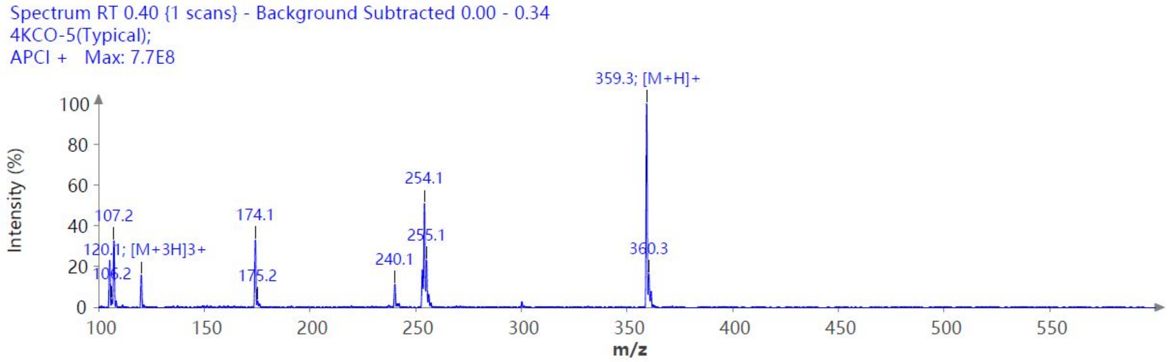

Figure S 56. APCI spectrum of **2l**.

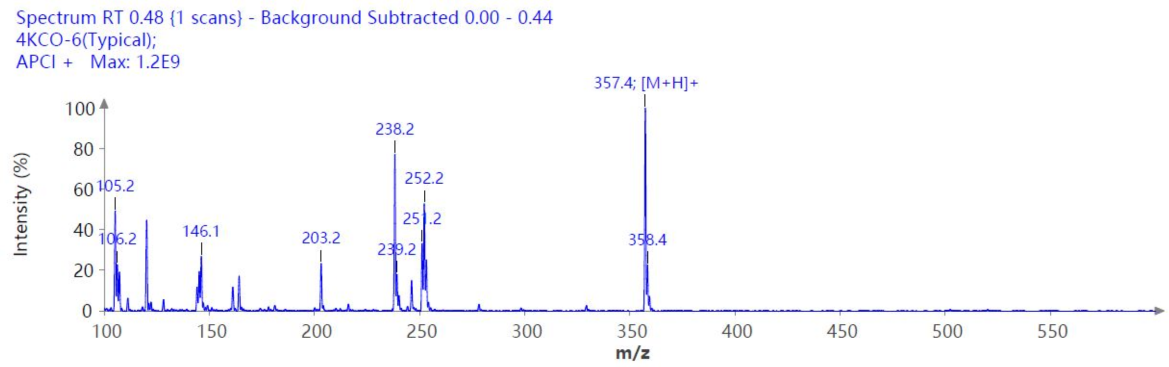

Figure S 57. APCI spectrum of **2m**.

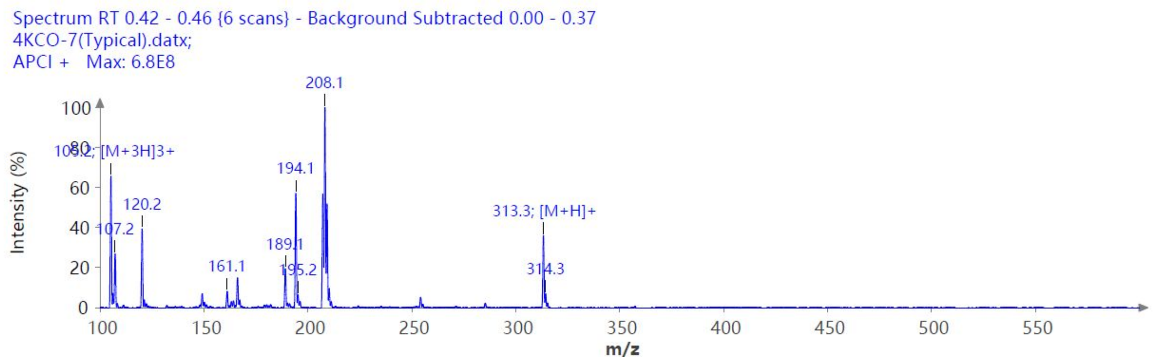

Figure S 58. APCI spectrum of **2n**.

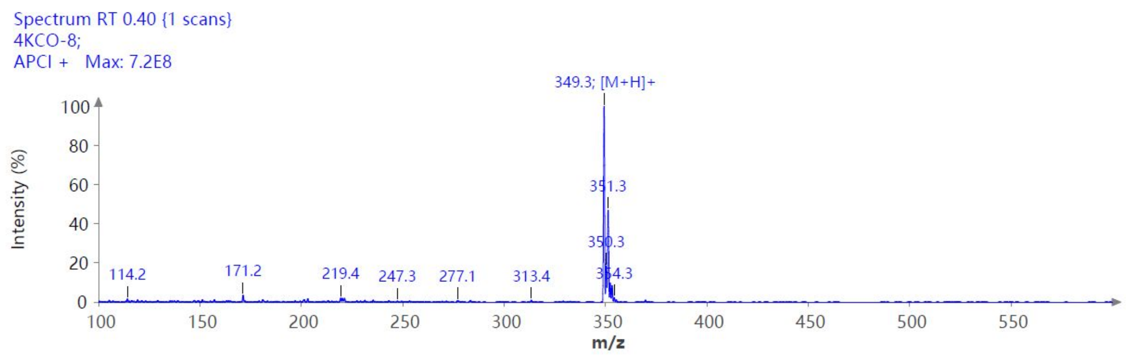

Figure S 59. APCI spectrum of **2o**.
